# TMT-based quantitative proteomics revealed follicle-stimulating hormone (FSH)-related molecular characterizations for potentially prognostic assessment and personalized treatment of FSH-positive non-functional pituitary adenomas

**DOI:** 10.1007/s13167-019-00187-w

**Published:** 2019-08-29

**Authors:** Ya Wang, Tingting Cheng, Miaolong Lu, Yun Mu, Biao Li, Xuejun Li, Xianquan Zhan

**Affiliations:** 1 Key Laboratory of Cancer Proteomics of Chinese Ministry of Health, Xiangya Hospital, Central South University, 87 Xiangya Road, Changsha, Hunan 410008 People’s Republic of China; 2 Hunan Engineering Laboratory for Structural Biology and Drug Design, Xiangya Hospital, Central South University, 87 Xiangya Road, Changsha, Hunan 410008 People’s Republic of China; 3 State Local Joint Engineering Laboratory for Anticancer Drugs, Xiangya Hospital, Central South University, 87 Xiangya Road, Changsha, Hunan 410008 People’s Republic of China; 4 Department of Neurosurgery, Xiangya Hospital, Central South University, 87 Xiangya Road, Changsha, Hunan 410008 People’s Republic of China; 5 National Clinical Research Center for Geriatric Disorders, Xiangya Hospital, Central South University, 88 Xiangya Road, Changsha, Hunan 410008 People’s Republic of China

**Keywords:** Nonfunctional pituitary adenomas, Follicle-stimulating hormone (FSH), Invasiveness, Tandem mass tag (TMT), Quantitative proteomics, Molecular network, Prognostic assessment, Personalized treatment, Predictive preventive personalized medicine, Evidence-based prognosis, Patient stratification, Targeted treatment

## Abstract

**Background:**

Non-functional pituitary adenoma (NFPA) is highly heterogeneous with different hormone expression subtypes. Of them, follicle-stimulating hormone (FSH)-positive expression is an important subtype of NFPAs. It is well-known that FSH exerted its functions through binding its receptor. However, the expression rate of FSH receptor was significantly higher in aggressive pituitary adenomas. This study aimed to investigate the molecular characteristics of FSH-positive NFPAs for effective stratification of patient, target treatment, prognostic assessment, and personalized treatment of FSH-positive NFPAs.

**Methods:**

Tandem mass tag (TMT)-based quantitative proteomics was used to investigate differentially expressed proteins (DEPs) between FSH-positive and negative NFPAs. Gene ontology and KEGG pathway enrichment analyses were used to analyze the DEPs. Differentially expressed genes (DEGs) between invasive and non-invasive NFPAs from GEO database were analyzed with pathway enrichment analysis. Protein-protein interaction (PPI) networks were constructed based on DEPs in excetral cellular matrix (ECM)-receptor interaction, focal adhesion, and PI3K-Akt pathways. Cytoscape was used to obtain most significant modules. Western blot was used to validate the expressions of upregulated proteins (ITGA1, ITGA6, and ITGB4), the expression and phosphorylated status of Akt in PI3K-Akt pathway, and the expression of FSH receptors in FSH-positive relative to negative NFPAs.

**Results:**

A total of 594 DEPs (374 upregulated and 220 downregulated) were identified between FSH-positive and negative NFPAs. Nineteen KEGG pathway networks were identified to involve DEPs, and reveal molecular differences between FSH-positive and negative NFPAs, including three important pathways that were significantly associated with tumor invasiveness and aggressiveness: ECM-receptor interaction, focal adhesion, and PI3K-Akt signaling pathways. Further, focal adhesion pathway was also confirmed with invasiveness-related NFPA DEG data that were derived from GEO database. Moreover, the significantly upregulated DEPs (ITGA1, ITGA6, and ITGB4) that were associated with tumor invasiveness and aggressiveness were confirmed by immunoaffinity analysis in FSH-positive vs. negative NFPAs. Also, the phosphorylation level but not its expression level of AKT in PI3K-AKT signaling was significantly increased, and the expression level of FSH receptor was significantly increased in FSH-positive relative to negative NFPAs. Also, overlapping analysis of 594 DEPs and 898 DEGs revealed 45 invasiveness-related DEPs, including 11 upregulated DEPs (ITGA6, FARP1, PALLD, PPBP, LIMA1, SCD, UACA, BAG3, CLU, PLEC, and GATM) that were also upregulated genes in invasive NFPAs, and 8 downregulated DEPs (ALCAM, HP, FSTL4, IL13RA2, NPTX2, DPP6, CRABP2, and SLC27A2) that were also downregulated genes in invasive NFPAs.

**Conclusions:**

FSH-positive expression was an important NFPA subtype. It was the first time for this study to reveal FSH-related proteomic variations and the corresponding molecular network alterations in FSH-positive relative to negative NFPAs. Also, three signaling pathways (ECM-receptor interaction, focal adhesion, and PI3K-Akt signaling pathways) and involved upregulated proteins (ITGA1, ITGA6, ITGB4, pAKT, and FSHR) were significantly associated with tumor invasiveness and aggressiveness, and a set of invasiveness-related DEPs were identified with overlapping analysis of 594 DEPs in FSH-positive vs. negative NFPAs and 898 DEGs in invasive vs. non-invasive NFPAs. These findings offered the scientific evidence to in-depth understand molecular characteristics of FSH-positive NFPAs, and effectively stratify the post-surgery patients for personalized prognostic assessment and targeted treatment of FSH-positive NFPAs.

**Electronic supplementary material:**

The online version of this article (10.1007/s13167-019-00187-w) contains supplementary material, which is available to authorized users.

## Introduction

Pituitary adenomas (PAs) account for approximately 10% of intracranial tumors [1], and are clinically divided into functional PAs (FPAs) and non-functional PAs (NFPAs) [2]. Compared to FPAs that generally secrete a significant amount of hormone and cause severe life-threatening clinical syndromes such as acromegaly or Cushing’s disease, NFPAs do not clinically elevate the level of hormones, which cause damage commonly through compression of regional structures. Thus, NFPA patients are not easy to be diagnosed at the early-stage, but often diagnosed at the middle/late stage when the intracranial compression symptoms appear, which results in losing the opportunity for early-stage treatment. Moreover, NFPAs are commonly benign, and neurosurgery is an efficient therapeutic approach to remove tumor. However, NFPAs are highly heterogeneous with different hormone expression subtypes. Some NFPAs have invasive and/or aggressive characteristics to injury or damage tumor-surrounding structures, thus these NFPA patients remain at risk for recurrence for several years after neurosurgery [3]. Therefore, invasiveness and aggressiveness are the very challenging clinical problems in treatment of NFPAs. Currently, the clinical diagnosis of invasiveness and aggressiveness of pituitary adenoma mainly depends on the image changes with nuclear magnetic resonance (NMR) and observation of tumor morphological changes in the process of neurosurgery [30–32]. Once this patient is stratified into invasive or aggressive NFPAs, then this patient will receive different treatment after neurosurgery. Actually, the use of image and morphological changes to determine invasive or aggressive characteristics are not fully correct; especially, it is very difficult to determine the invasiveness when this tumor is at its relative small size. It is urgently needed to use of molecular pattern changes for determination of the invasive or aggressive characteristics of NFPAs for patient stratification, prognostic assessment, and targeted treatment.

PA proteome has been extensively studied so far to reveal PA molecular changes, including protein expression profiles of pituitary tissues [4–7], identification of protein biomarkers in the sera for diagnosis of PAs [8, 9], protein post-translational modifications that include tyrosine nitration [10–13], ubiquitination [71], and phosphorylation [14, 15], growth hormone proteoforms [16], and prolactin proteoforms [72]. Our long-term goal focuses on elucidation of molecular mechanisms and discovery of tumor-related NFPA biomarkers with proteomics. Some studies on NFPA proteomes have been carried out, including identification of serum-specific protein biomarkers to aid in the early diagnosis of NFPAs [17], identification of molecular signaling [18], identification of the proteomic variations of invasive relative to non-invasive NFPAs [19], and identification of differentially expressed proteins (DEPs) among different NFPAs subtypes [20]. Our previous two studies [19, 20] were derived from two-dimensional gel electrophoresis (2DGE)-based comparative proteomics. One previous study [19] only considered the invasive vs. non-invasive NFPAs, but did not considered the different hormone-expression subtypes of NFPAs; actually, both invasive and non-invasive NFPAs contained different hormone-expression subtypes of NFPAs. Another previous study [20] considered different hormone-expression subtypes (NF^−^, FSH^+^, LH^+^, and FSH^+^/LH^+^; NF^−^ means no any pituitary hormone expressions; FSH^+^ means only expression of follicle stimulating hormone (FSH); LH^+^ means only expression of luteinizing hormone (LH); and FSH^+^/LH^+^ means only expressions of both FSH and LH in pituitary adenoma tissue) of NFPAs compared to control pituitaries (Con), respectively, and compared those 4 sets of DEPs (59 DEPs in NF compared to Con; 63 DEPs in FSH compared to Con; 65 DEPs in LH compared to Con; and 55 DEPs in LH/FSH compared to Con) to obtain 44 overlapped DEPs, which preliminary revealed that different hormone-expression subtypes of NFPAs had different molecular behaviors. However, these previous 2DGE-based comparative proteomics only obtained dozens of proteins, whose low-throughput was obviously an obstacle to fully understand the molecular mechanisms of different hormone-expression subtypes of NFPAs, and to identify more reliable and effective biomarkers for precise management of different hormone-expression subtypes of NFPAs. Therefore, it is necessary to use a high-throughput approach, such as peptide tandem mass tag (TMT)-based quantitative proteomics, for the analysis of a specific hormone-expression subtype (NF^−^, FSH^+^, LH^+^, or FSH^+^/LH^+^) of NFPAs to achieve the feature molecular profile changes of each NFPA subtype.

FSH belongs to anterior pituitary glycoprotein hormones, which is a disulphide-rich heterodimer consisting of non-covalently associated α and β subunits [21]. In females, FSH binds to its receptor (FSHR) and induces the maturation of ovarian follicles. In males, FSH plays an important role in establishment of the population of Sertoli cells and maintenance of the number and quality of sperms [22, 23]. FSHR is expressed in vascular endothelial cells in a wide range of tumors that are located in the prostate, breast, colon, pancreas, urinary bladder, kidney, lung, liver, stomach, testis, and ovary [24]. FSHR is also expressed in the majority of PAs in the endothelia of intra- and peri-tumoral blood vessels and/or tumor cells, and is positively correlated with PAs with higher Ki-67 index [25]. Moreover, analysis of FSHR expressions among different subtypes of PAs found that the incidence of FSHR expression was significantly higher in aggressive PAs (68%) than in non-aggressive PAs (12%). Thus, FSHR is considered as a marker of aggressiveness of PAs [26]. It is well-known that that FSH exerts its biological roles through binding to FSHR [27–29]. Currently, we do not know how FSH plays roles in NFPAs; further, whether FSH-positive expression NFPAs has the invasive molecular characteristics, which implies important scientific merits for in-depth investigation.

This study selected FSH-positive vs. negative NFPAs as a start point to investigate molecular characteristics of each NFPA subtype. TMT-based quantitative proteomics was used to identify DEP profile between FSH-positive vs. negative NFPAs without expressions of other pituitary hormones between two groups. Gene ontology (GO) and Kyoto Encyclopedia of Genes and Genomes (KEGG) pathway enrichment analyses of DEP data were used to reveal the FSH-related molecular characteristics in NFPAs, which were validated with Western blot experiments. Moreover, FSH-related DEPs were integrated with transcriptomic data—differentially expressed genes (DEGs) that were derived from comparison of invasive and non-invasive NFPAs from Gene Expression Omnibus (GEO) database, which was used to further explore the relationship of FSH and invasiveness in NFPAs. These findings offer the in-depth insight into the molecular mechanisms of progression of FSH-positive NFPAs, and provide important biomarker resource to effectively stratify patients for precisely personalized prognostic assessment and effectively targeted treatment of FSH-positive NFPAs.

## Methods

### NFPAs and protein extraction

Nine NFPA tissue samples (FSH^+^: *n* = 4; FSH^−^: *n* = 5; and all other pituitary hormones were negatively expressed in NFPA tissues) (Table 1) were obtained from the Department of Neurosurgery, Xiangya Hospital, Central South University, and approved by Xiangya Hospital Medical Ethics Committee of Central South University (Approval number: 2013030181). Each sample was grinded with liquid nitrogen, and then were transferred to 5-mL centrifuge tube and sonicated three times on ice through a high-intensity ultrasonic processor (Scientz) in lysis buffer [8 M urea, 2 mM ethylene diamine tetraacetic acid (EDTA), 10 mM dithiothreitol (DTT), and 1% protease inhibitor cocktail III]. The remained debris was removed by centrifugation (20,000 g, 4 °C, and 10 min). Finally, the proteins were precipitated with cold 15% trichloroacetic acid (TCA; 2 h, and − 20 °C). After centrifugation (4 °C, 10 min), the supernatant was discarded. The remained precipitate was washed with cold acetone for three times. Proteins were redissolved in the buffer [8 M urea, 100 mM tetraethyl ammonium bromide (TEAB), pH 8.0], and the protein concentration was determined with Bio-Rad 2-D Quant kit according to the manufacturer’s instructions.

**Table 1:** Clinical characteristics of NFPA tissue samples

Group	Sex	Age	Clinical characteristics	Immunohistochemistry	Experiments
FSH^+^	Male	40	NFPA in sellar region. Recurrent tumor, old blooding in tumor, and tumor size 2 × 2 × 1.8 cm^3^.	ACTH(−), hGH(−), PRL(−), FSH(+), LH(−), TSH(−)	Proteomics; Western blot
	Male	59	NFPA in sellar region. Sellar floor bone thinning, and enriched blood supply in tumor, and tumor size 2.1 × 1.8 × 2 cm^3^.	ACTH(−), hGH(−), PRL(−), FSH(+), LH(−), TSH(−)	Proteomics; Western blot
	Female	43	NFPA in sellar region. Compression of surrounding tissue, damage and adhesion of surrounding tissues, and tumor size 4.5 × 4 × 6 cm^3^.	ACTH(−), hGH(−), PRL(−), FSH(+), LH(−), TSH(−)	Proteomics; Western blot
	Female	44	NFPA in sellar region.	ACTH(−), hGH(−), PRL(−), FSH(+), LH(−), TSH(−)	Western blot
FSH^−^	Male	49	NFPA in sellar region. Sellar floor bone thinning, old blooding in tumor, and tumor size 2 × 4 × 3 cm^3^.	ACTH(−), hGH(−), PRL(−), FSH(−), LH(−), TSH(−)	Proteomics; Western blot
	Male	58	NFPA in sellar region. Sellar floor bone thinning, enriched blood supply, and tumor size 4.5 × 3 × 3 cm^3^.	ACTH(−), hGH(−), PRL(−), FSH(−), LH(−), TSH(−)	Proteomics; Western blot
	Female	53	NFPA in sellar region. Sellar floor bone thinning, enriched blood supply, and tumor size 3 × 3.5 × 2.5 cm^3^.	ACTH(−), hGH(−), PRL(−), FSH(−), LH(−), TSH(−)	Proteomics; Western blot
	Male	53	NFPA in sellar region.	ACTH(−), hGH(−), PRL(−), FSH(−), LH(−), TSH(−)	Western blot
	Female	45	NFPA in sellar region.	ACTH(−), hGH(−), PRL(−), FSH(−), LH(−), TSH(−)	Western blot

*NFPA* non-functional pituitary adenoma

### Trypsin digestion

The proteins in the solution were reduced with 10 mM DTT (1 h, 37 °C), and alkylated with 20 mM iodoacetamide (45 min, room temperature) in darkness. For trypsin digestion, the protein sample was diluted by adding 100 mM TEAB to let the solution of less than 2 M urea. Finally, the proteins were digested with trypsin in a mass ratio of trypsin to protein (1:50) for the first-round digestion overnight, and then digested in a mass ratio of trypsin to protein (1:100) for a second-round digestion for 4 h. Approximately 100 μg proteins for each sample were digested with trypsin for the following experiments.

### TMT labeling

After trypsin digestion, peptides were desalted by Strata X C18 SPE column (Phenomenex), and vacuum-dried. The dried peptides were redissolved in 0.5 M TEAB, and processed according to the manufacturer’s protocol for a 6-plex TMT kit. Briefly, one unit of TMT reagent (It was used to label the tryptic peptides of 100 μg proteins) were thawed and reconstituted in 24 μL acetonitrile (ACN). The peptide mixture was then incubated with the prepared TMT reagent (2 h, room temperature), then TMT-labeled peptide mixtures were pooled equally (1:1:1:1:1:1), desalted, and dried by vacuum centrifugation.

### HPLC fractionation and LC-MS/MS

The prepared TMT-labeled tryptic peptide mixture was fractionated into 18 simplified samples with high pH reverse-phase high-performance liquid chromatography (HPLC) with Agilent 300 Extend C18 column (5 μm particles, 4.6 mm i.d., 250 mm length). Briefly, TMT-labeled peptide mixture was first separated with a gradient of 2% to 60% ACN in 10 mM ammonium bicarbonate pH 10 over 80 min into 80 fractions. Those 80 fractions were combined into 18 fractionated simplified samples, and dried by vacuum centrifugation.

Each simplified sample was dissolved in 0.1% trifluoroacetic acid (TFA), directly loaded onto a reversed-phase pre-column (Acclaim PepMap 100, Thermo Scientific). Peptide separation was performed with a reversed-phase analytical column (Acclaim PepMap RSLC, Thermo Scientific). The gradient was comprised of an increase from 5 to 25% solvent B (0.1% TFA in 98% ACN) over 60 min, 25% to 35% solvent B in 12 min and climbing to 80% solvent B in 4 min, and then holding at 80% solvent B for the last 4 min, with a constant flow rate of 320 nL/min on an EASY-nLC 1000 UPLC system. The separated peptides were online subjected to Orbitrap Fusion™ mass spectrometer (ThermoFisher Scientific) to obtain tandem mass spectrometry (MS/MS) data of each peptide. Briefly, the HPLC-separated peptides were online subjected to neutral spray ionization (NSI), followed by acquirement of MS/MS spectra. The tryptic peptide ions (precursor ions) were detected in the MS spectrum at a resolution of 70,000. The precursor ions with intensity at least 5E4 in MS spectrum were selected for high energy collision dissociation (HCD) at a collision-energy 38 to obtain MSMS spectra; and the product ions were detected at a resolution of 15,000. The electrospray voltage was set as 2.0 kV. Automatic gain control (AGC) was used to prevent overfilling of the OrbiTrap. The primary MS scan range (MS spectrum) was within *m*/*z* 400 to 1600. The start point of the secondary MS scan (MS/MS spectrum) was set as *m*/z 100.

### Database search of MS/MS data and determination of DEPs

MS/MS data were processed with Mascot search engine (v.2.3.0) against *Swiss*-*Prot Human* database for protein identification. Trypsin/P was specified as cleavage enzyme allowing up to two missing cleavages. Mass error was set to 10 ppm for precursor ions and 0.02 Da for product ions. Carbamidomethyl on Cys was specified as fixed modification, and oxidation on Met was specified as variable modification. TMT-6-plex was set as variable modification. For protein identification, false discovery rate (FDR) was adjusted to < 1% and peptide ion score was set ≥ 20. For determination of DEPs, the reproducibility was analyzed for this TMT-based quantitative proteomics to determine the cutoff value of change-fold between FSH+ and FSH-NFPAs; Student *t* test was used to calculate the *p* value of each DEP between FSH-positive and negative NFPAs, and *p* < 0.05 was considered as statistical significance.

### Bioinformatics analysis of DEPs

**GO** annotation of DEPs was derived from the UniProt-GOA database (http://www.ebi.ac.uk/GOA/). DEPs were classified by GO annotation based on three categories, including biological processes (BPs), cellular compartments (CCs), and molecular functions (MFs), and the *p* < 0.05 was considered as statistical significance. KEGG pathway analysis of DEPs was performed with KOBAS online analysis database (http://kobas.cbi.pku.edu.cn/), and a Benjamini-Hochberg corrected *p* < 0.05 was considered as statistical significance.

Protein-protein interaction (PPI) network of DEPs was constructed with online STRING database (https://string-db.org), and an interaction with a combined score > 0.4 was considered as statistical significance. Cytoscape, an open source bioinformatic software platform, was used to visualize molecular interaction networks. The plug-in Molecular Complex Detection (MCODE) in Cytoscape software was used to cluster a given network based on topology to find densely connected regions. The PPI networks were drawn with Cytoscape, and the most significant modules in the PPI networks were identified with MCODE method with the default criteria, including MCODE score > 5, degree cutoff value = 2, node score cutoff value = 0.2, Max depth = 100, and *k*-score = 2.

### GEO gene data analysis between invasive and non-invasive NFPAs

Three keywords “NFPA,” “non-functional pituitary adenomas,” and “non-functioning pituitary adenomas” were used to search **GEO** database (https://www.ncbi.nlm.nih.gov/geo/) to obtain the corresponding search results of 13 items, 4 items, and 51 items, followed by comprehensive analysis of those 68 (13 + 4 + 51) items including removal of the repeated items. Only one dataset GSE51618 met our requirements, which included three invasive NFPAs (*n* = 3), four non-invasive NFPAs (*n* = 4), and three normal controls (*n* = 3), and was downloaded from GEO database for subsequent analysis. The R software package was used to process the downloaded files and to convert and reject the unqualified data. DEGs between invasive and non-invasive NFPAs were determined according to the threshold: FDR < 0.05, and fold-changes with a log2 absolute of fold-change of ≥ 2. KEGG pathway enrichment of DEGs was carried out with KOBAS online analysis database, and a Benjamini-Hochberg corrected *p* < 0.05 was considered as statistical significance.

### Validation of DEPs between FSH-positive and negative NFPAs with Western blot

Proteins were extracted from NFPA samples (Table 1) with the tissue total protein lysis buffer (Sangon Biotech, China) plus protease inhibitors and phosphatase inhibitors. Protein concentration was measured with bicin-choninic acid (BCA) protein assay kit. The equal amounts of proteins from FSH-positive and negative NFPAs were separated with 6% or 10% sodium dodecyl sulfonate-polyacrylamide gel electrophoresis (SDS-PAGE). The separated proteins were transferred to polyvinylidene difluoride (PVDF) membranes (0.45 μm; GE Healthcare), and incubated with primary antibodies against human ITGA1 (Cusabio), ITGA6 (RnD), ITGB4 (Cusabio), FSHR (Novus), AKT1/AKT2/AKT3 (Cusabio), phospho-AKT1/AKT2/AKT3 (S473) (Cusabio), and β-actin (Santa Cruz Biotechnology) (1:1000 dilution for each one) at 4 °C overnight, and followed by incubation for 2 h with horseradish peroxidase-conjugated secondary antibody (1:5000 dilution) at room temperature. Protein bands were detected with an enhanced chemiluminescence (ECL) system. Data with normal distribution were presented as mean ± SD. Student’s *t* test was used for between-group comparison with a statistical significance of *p* < 0.05.

## Results

### Reproducibility of TMT-based quantitative proteomics in analyses of FSH-positive vs. negative NFPA samples

**Fig. 1:**
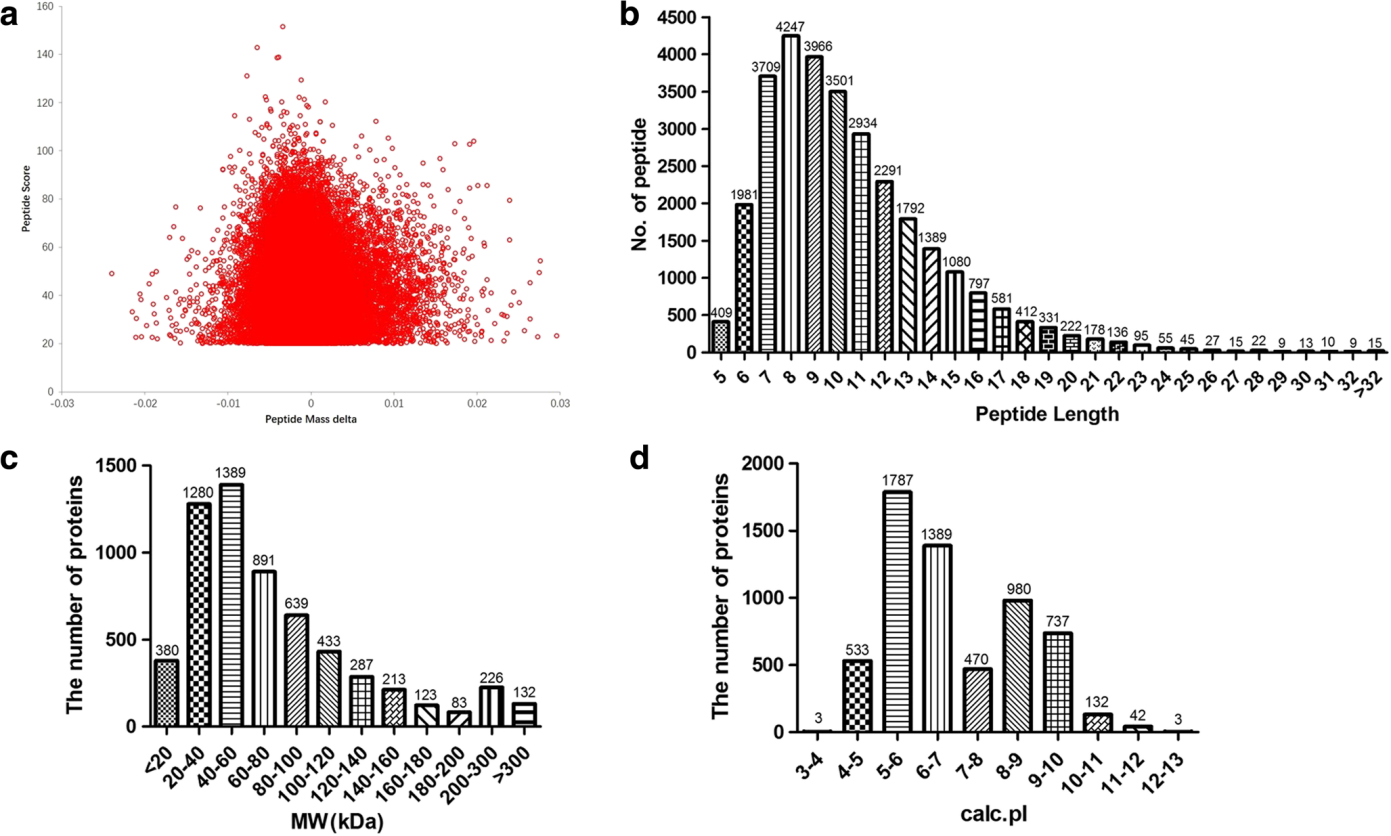
The information of the identified tryptic peptides and proteins. **a** Distribution of mass error of identified tryptic peptides. **b** Distribution of amino acid length of identified tryptic peptides. **c** Distribution of molecular weight of identified proteins. **d** Distribution of pI of identified proteins

The reliability of identified proteins was mainly derived from mass spectrometry (MS) analysis. For this study, a total of 479,505 MS/MS spectra were obtained, of them 60,244 MS/MS spectra were matched to 30,265 tryptic peptides, which contained 28,707 unique peptides. The mass error of all qualified tryptic peptides was normally distributed in the central axis of 0 with the range of − 0.02 to 0.02 Da (Fig. 1a), which means the mass error of the tryptic peptides met the requirement, and mass spectrometer ran in good condition. The length of tryptic peptides was mainly among 6–20 amino acid residues (Fig. 1b), which was in accordance with the rule of trypsin digestion of proteins. Under the good MS condition, a total of 6076 proteins were identified from those qualified tryptic peptides, and most of those proteins were distributed within a range of molecular weight 7–200 Da (Fig. 1c) and isoelectric point (p*I*) within a range of pH 4–12 (Fig. 1d).

The reproducibility of TMT-based quantitative proteomics was the prerequisite to determine a reliable DEP. For this study, each group (FSH^+^ or FSH^−^) was analyzed three times by TMT-based quantitative proteomics, respectively. A total of 4666 proteins were quantified between FSH^+^ and FSH^−^ NFPAs. For each quantified protein, its CV, correlation coefficient (*r*), and fold-change were calculated in each group to evaluate the reproducibility of TMT-based quantitative proteomics. The CV was under 10% for more than 95% proteins, and under 20% for more than 99% proteins in each group (Table 2). The averaged *r* of quantified proteins was 0.92 in each group (Table 2), which suggested that three repeated experiments in each group were highly correlated, and there was in accordant trend between two groups. Moreover, the fold-change was under 1.3-fold for more than 99% proteins, and under 1.6-fold for 100% proteins in each group (Table 2), which means that 1.6-fold cutoff value was able to eliminate the experimental error, and that the fold-change ≥ 1.6-fold or ≤ − 1.6-fold reflected the true biological difference between FSH^+^ and FSH^−^ NFPAs. Therefore, the cutoff value 1.6-fold plus a statistically significant level of *p* < 0.05 was used to determine each DEP.

**Table 2:** Coefficient of variation (CV), correlation coefficient (*r*), and fold-change of quantified proteins in FSH^+^ and FSH^−^ NFPA group

		FSH^+^	FSH^−^
CV	< 10%	4511 (96.68%)	4459 (95.56%)
	< 20%	4650 (99.66%)	4648 (99.61%)
	< 30%	4663 (99.94%)	4658 (99.83%)
	< 40%	4666 (100%)	4666 (100%)
*r*	1 vs. 2	0.91	0.90
	1 vs. 3	0.94	0.93
	2 vs. 3	0.92	0.93
	Average	0.92	0.92
Fold-change	< 1.2	4612 (98.84%)	4590 (98.37%)
	< 1.3	4654 (99.74%)	4647 (99.59%)
	< 1.4	4662 (99.91%)	4656 (99.79%)
	< 1.5	4665 (99.98%)	4658 (99.83%)
	< 1.6	4666 (100.00%)	4666 (100.00%)

*FSH* follicle-stimulating hormone, *NFPA* non-functional pituitary adenoma

### DEP profiling between FSH-positive vs. negative NFPAs

According to the determined cutoff value, fold-change ≥ 1.6-fold (ratio ≥ 1.6) or ≤ − 1.6-fold (ratio ≤ 0.625) plus *p* value < 0.05 was used to determine a DEP in FSH-positive relative to negative NFPAs, which identified a total of 594 DEPs, including 374 upregulated and 220 downregulated DEPs (Supplemental Table 1).

**Fig. 2:**
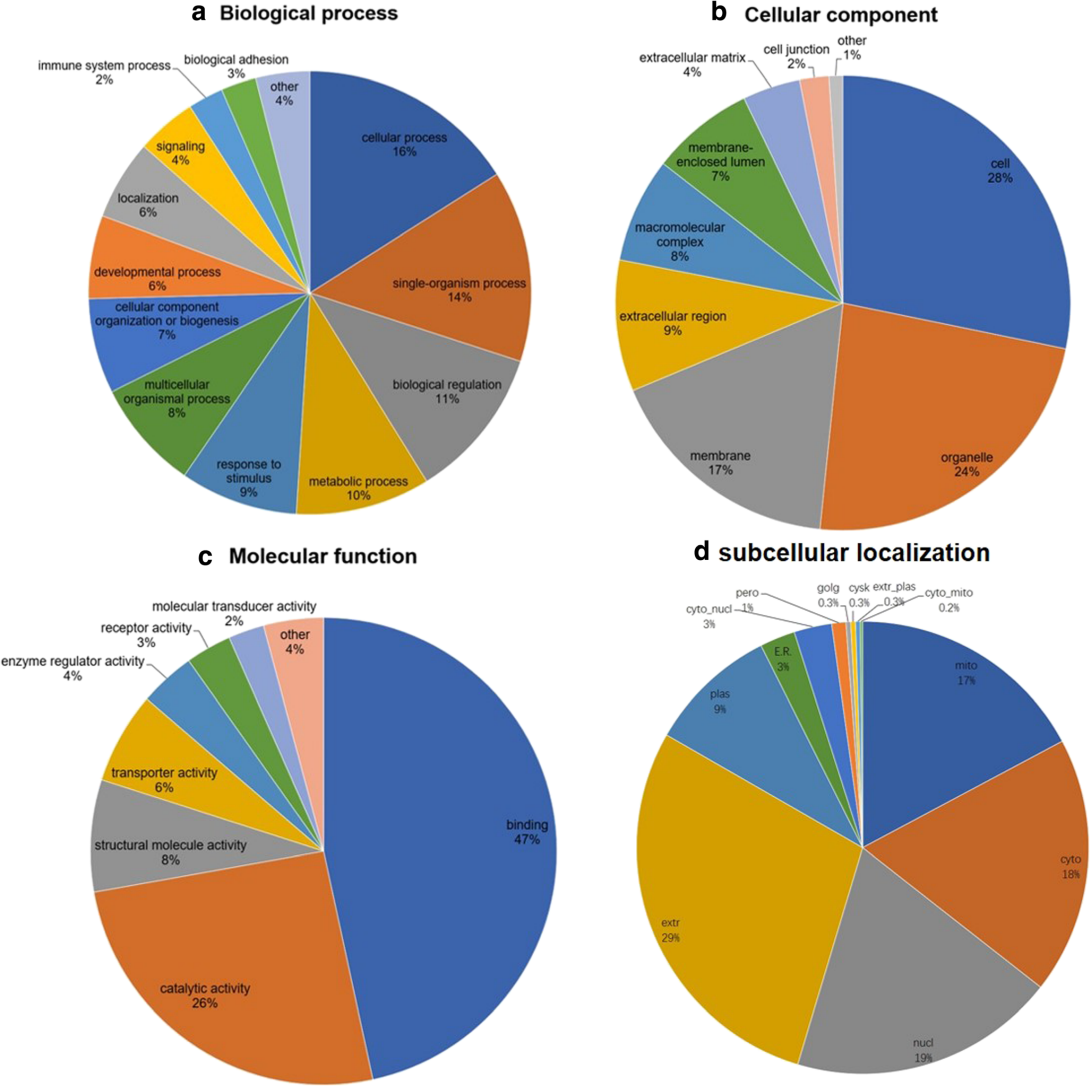
GO enrichments and subcellular location of DEPs. **a** GO enrichments in biological process (BP). **b** GO enrichments in cellular components (CC). **c** GO enrichments in molecular functions (MF). **d** The subcellular location of DEPs

The overall functional characteristics of DEPs were annotated by GO enrichment analysis according to BP, CC, and MF. For BP enrichment, DEPs were mainly involved in cellular process (16%), single-organism process (14%), biological regulation (11%), metabolic process (10%), response to stimulus (9%), multicellular organismal process (8%), cellular component organization or biogenesis (7%), and developmental process (6%) (Fig. 2a). For CC enrichment, DEPs were mainly involved in cell (28%), organelle (24%), membrane (17%), extracellular region (9%), macromolecular complex (8%), and membrane-enclosed lumen (7%) (Fig. 2b). For MF enrichment, DEPs were primarily related to binding (47%), catalytic activity (26%), structural molecule activity (8%), and transporter activity (6%) (Fig. 2c).

A subcellular localization predication soft Wolfpsort was also used to predict subcellular localization of DEPs. DEPs were mainly localized to extracellular matrix (29%), nuclear (19%), cytosol (18%), mitochondria (17%), plasma membrane (9%), cytosol and nuclear (3%), endoplasmic reticulum (3%), peroxisome (1%), Golgi apparatus (0.3%), cytoskeleton (0.3%), extracellular and plasma membrane (0.3%), and cytosol and mitochondria (0.2%) (Fig. 2d).

### FSH-related signal pathway alterations in FSH-positive NFPAs

**Fig. 3:**
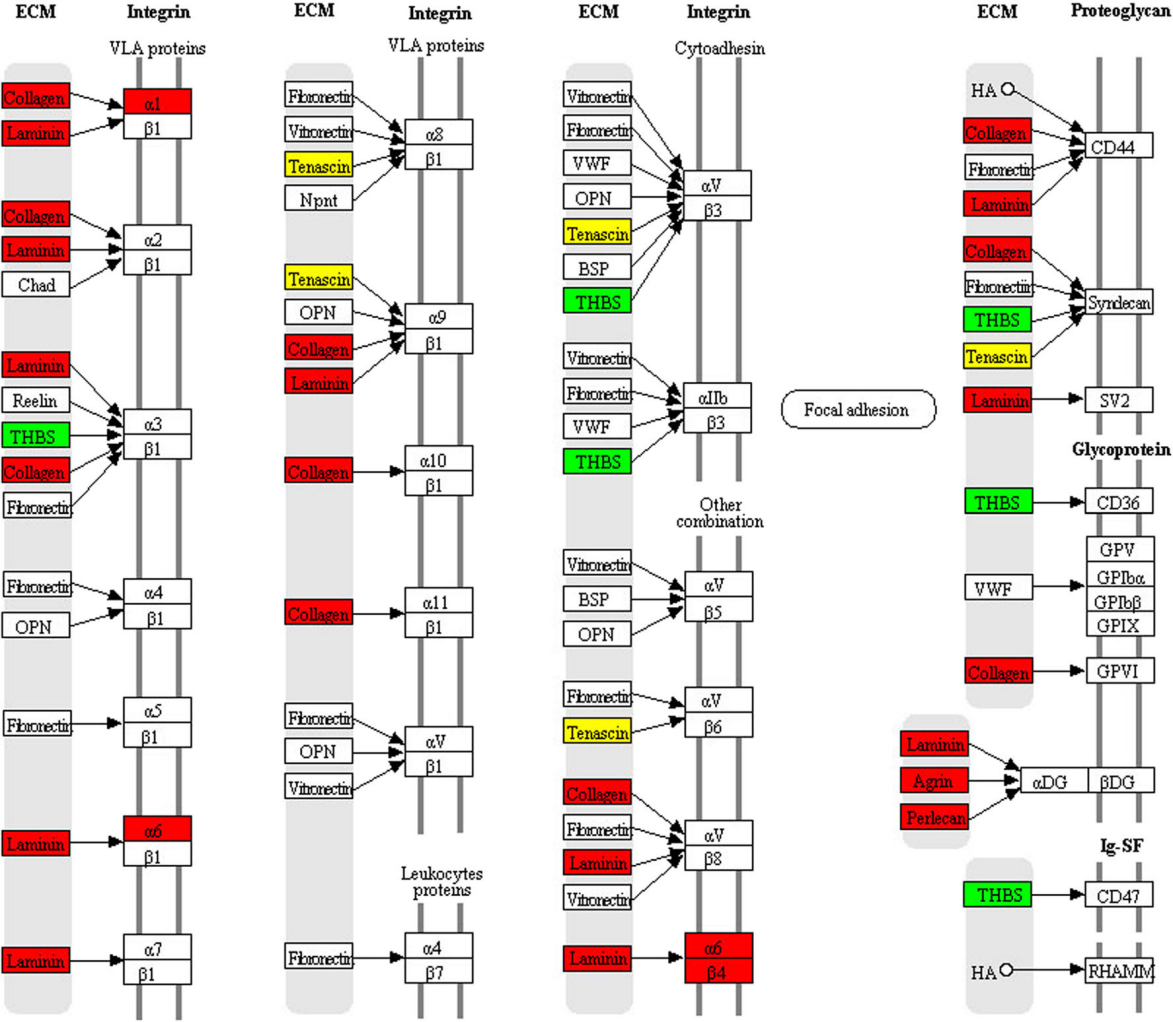
ECM-receptor interaction pathway changed in FSH-positive relative to negative NFPAs**.** Red: upregulated DEP. Blue: downregulated DEP. Yellow: some family members of that molecule were upregulated, and some family members of that molecule were downregulated

**Fig. 4:**
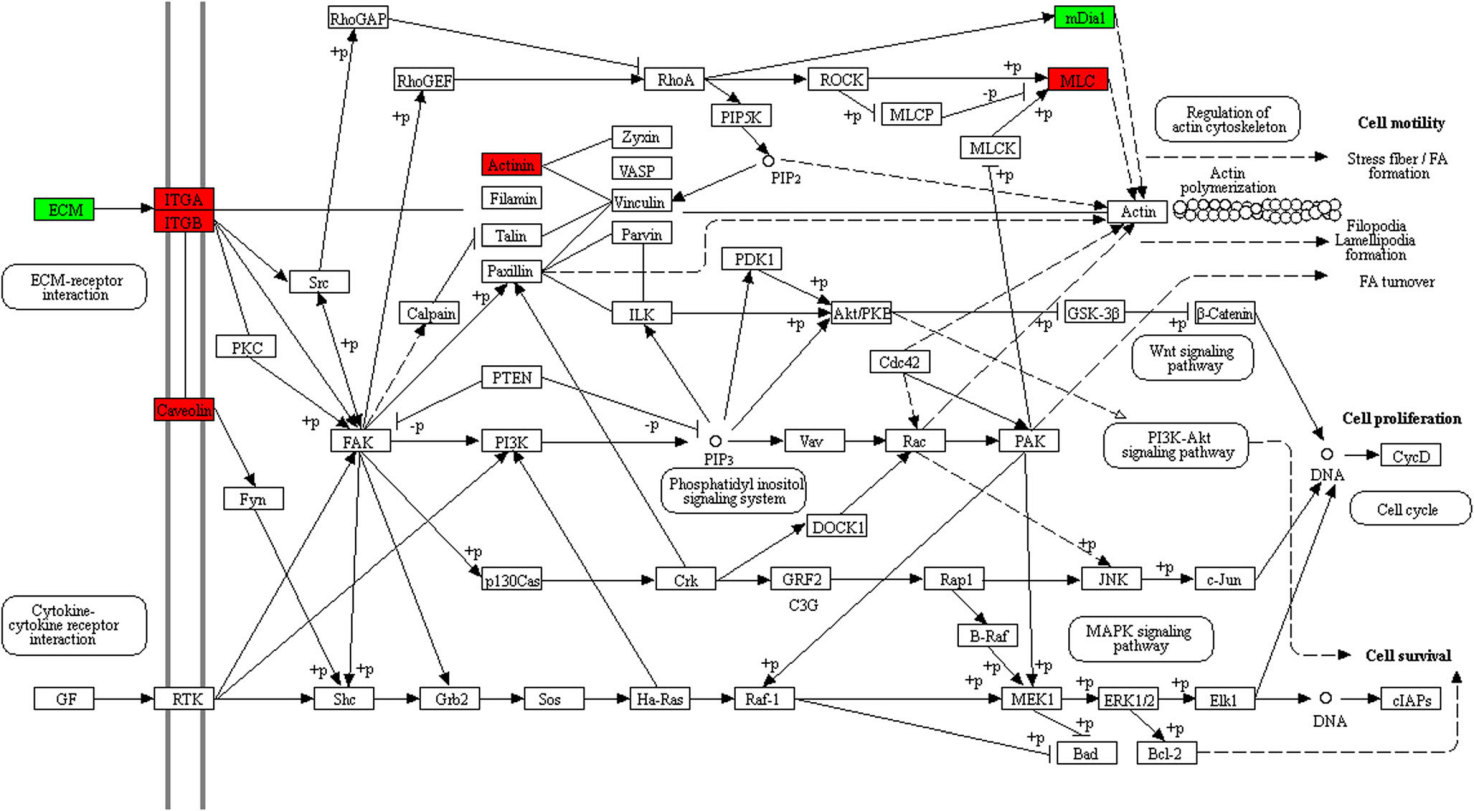
Focal adhesion pathway changed in FSH-positive relative to negative NFPAs. Red: upregulated DEP. Blue: downregulated DEP

**Fig. 5:**
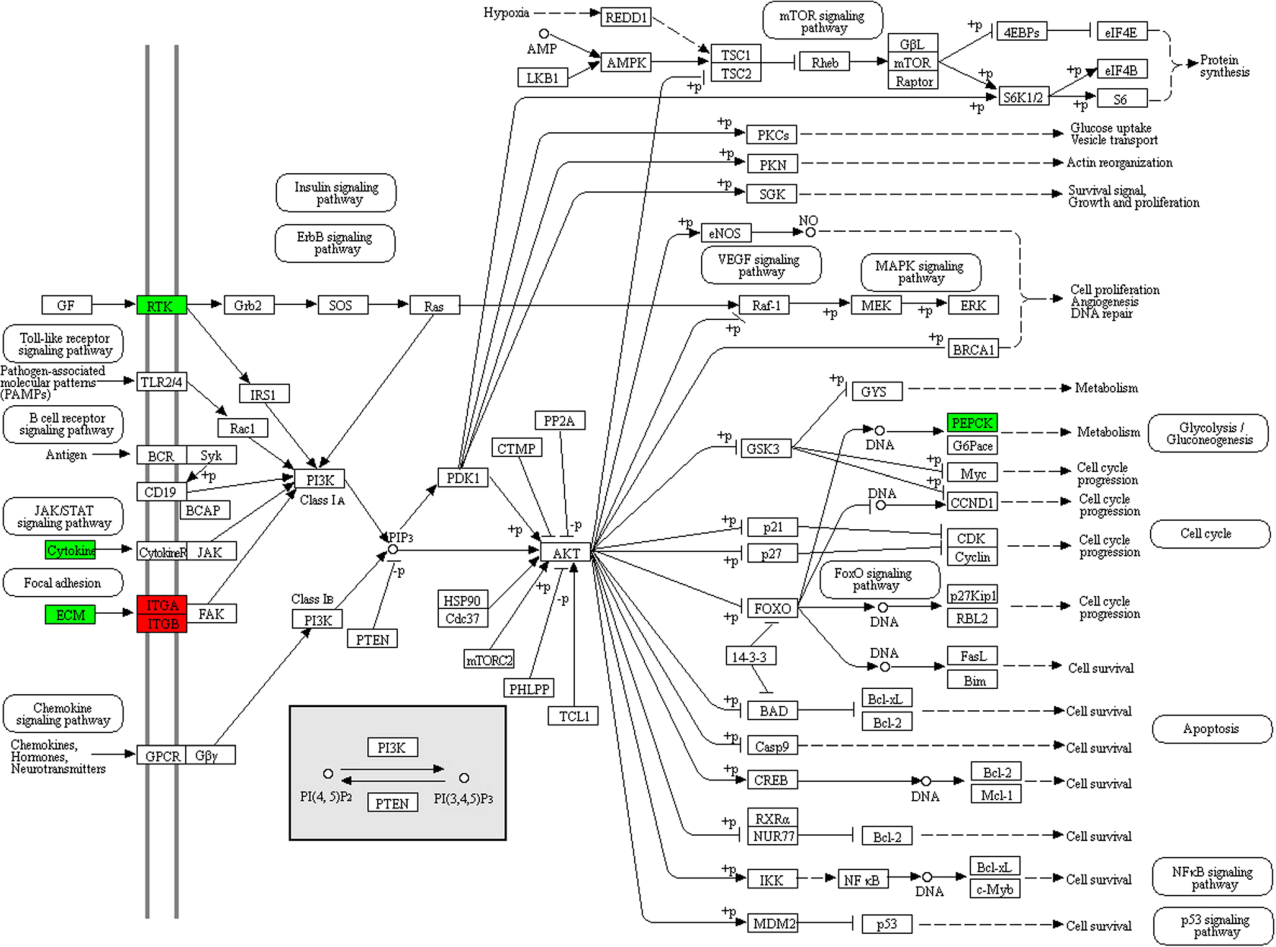
PI3K-Akt signaling pathway changed in FSH-positive relative to negative NFPAs. Red: upregulated DEP. Blue: downregulated DEP

KEGG pathway enrichment was used to analyze those 594 DEPs between FSH**-**positive vs. negative NFPAs. Nineteen statistically significant pathways were identified (Table 3, Supplemental Fig. 1), including excellular matrix (ECM)-receptor interaction, facal adhesion, PI3K-Akt signaling pathway, protein digestion and absorption, amoebiasis, PPAR signaling pathway, fatty acid metabolism, immune network for IgA production, **co**mplement and coagulation cascades, cell adhesion molecules, and hypertrophic tubule bicarbonate reclamation. Of them, three pathways were obviously associated with tumorigenesis, invasiveness, or aggressiveness, including ECM-receptor interaction (Fig. 3, Table 4), focal adhesion (Fig. 4, Table 5), and PI3K-Akt signaling pathways (Fig. 5, Table 6).

**Table 3:** Statistically significant signaling pathways identified by KEGG pathway enrichment analysis

KEGG pathway	Mapping	Fold enrichment	Fisher’ exact test *p* value
hsa04512: ECM-receptor interaction	32	5.72	1.52E-17
hsa04510: Focal adhesion	35	3.05	1.39E-9
hsa04151: PI3K-Akt signaling pathway	34	2.68	9.13E-8
hsa04514: Cell adhesion molecules (CAMs)	11	2.00	4.19E-2
hsa03320: PPAR signaling pathway	11	2.70	5.44E-3
hsa01212: Fatty acid metabolism	10	2.71	8.68E-3
hsa04672: Intestinal immune network for IgA production	5	5.28	1.02E-2
hsa04610: Complement and coagulation cascades	11	2.42	1.21E-2
hsa04974: Protein digestion and absorption	18	5.28	3.73E-9
hsa05146: Amoebiasis	22	3.74	7.11E-8
hsa05222: Small cell lung cancer	16	3.93	3.77E-6
hsa05310: Asthma	6	7.04	6.61E-4
hsa05145: Toxoplasmosis	14	2.69	1.34E-3
hsa05412: Arrhythmogenic right ventricular cardiomyopathy (ARVC)	12	2.94	1.58E-3
hsa04640: Hematopoietic cell lineage	10	3.4	1.61E-3
hsa05322: Systemic lupus erythematosus	11	2.64	6.46E-3
hsa05410: Hypertrophic cardiomyopathy (HCM)	9	2.21	4.44E-2
hsa04964: Proximal tubule bicarbonate reclamation	5	3.52	4.59E-2
hsa05416: Viral myocarditis	7	2.55	4.95E-2

*ECM* extracellular matrix

**Table 4:** DEPs involved in ECM-receptor interaction pathway

Protein accession	Protein description	Gene name	MW (kDa)	Calc. pI	Changed fold (FSH^+^/FSH^−^)
O00468	Agrin	AGRN	236.02	6.01	3.18
P02452	Collagen alpha-1(I) chain	COL1A1	153.17	5.6	2.11
P08123	Collagen alpha-2(I) chain	COL1A2	141.44	9.08	2.07
P02458	Collagen alpha-1(II) chain	COL2A1	158.37	6.58	4.00
P02461	Collagen alpha-1(III) chain	COL3A1	154.17	6.21	1.71
P02462	Collagen alpha-1(IV) chain	COL4A1	183.2	8.55	2.35
P08572	Collagen alpha-2(IV) chain	COL4A2	187.44	8.89	2.01
Q01955	Collagen alpha-3(IV) chain	COL4A3	183.7	9.28	2.58
P53420	Collagen alpha-4(IV) chain	COL4A4	185.01	8.9	4.48
Q14031	Collagen alpha-6(IV) chain	COL4A6	187.13	9.31	3.77
P05997	Collagen alpha-2(V) chain	COL5A2	160	6.07	2.06
P12109	Collagen alpha-1(I) chain	COL6A1	122.21	5.26	1.88
P12110	Collagen alpha-2(VI) chain	COL6A2	122.08	5.85	1.82
P12111	collagen alpha-3(VI) chain	COL6A3	381.6	6.26	1.96
P98160	Basement membrane-specific heparan sulfate proteoglycan core protein	HSPG2	495.06	6.06	1.94
P56199	Integrin alpha-1	ITGA1	149.03	5.91	1.74
P23229	Integrin alpha-6	ITGA6	144.45	6.21	2.31
P16144	Integrin beta-4	ITGB4	220.64	5.74	2.60
P25391	Laminin subunit alpha-1	LAMA1	379.1	5.93	2.73
P24043	Laminin subunit alpha-2	LAMA2	395.83	6.01	4.62
Q16787	Laminin subunit alpha-3	LAMA3	410.94	7.03	1.98
Q16363	Laminin subunit alpha-4	LAMA4	228.85	5.89	1.75
O15230	Laminin subunit alpha-5	LAMA5	426.69	6.66	2.35
P07942	Laminin subunit beta-1	LAMB1	224.86	4.83	1.88
P55268	Laminin subunit beta-2	LAMB2	210.77	6.07	2.95
P11047	Laminin subunit gamma-1	LAMC1	201.98	5.01	2.72
Q13753	Laminin subunit gamma-2	LAMC2	147.14	5.83	2.35
P35442	Thrombospondin-2	THBS2	144.1	4.62	− 5.88
P24821	Tenascin	TNC	265.37	4.79	2.30
Q92752	Tenascin-R	TNR	159.6	4.71	− 2.00
Q9UQP3	Tenascin-N	TNN	162.27	5.41	2.16
P22105	Tenascin-X	TNXB	492.67	5.05	2.07

*DEP* differentially expressed protein, *ECM* extracellular matrix, *MW* molecular weight, *FSH* follicle stimulating hormone

**Table 5:** DEPs involved in focal adhesion pathway

Protein accession	Protein description	Gene name	MW (kDa)	calc. pI	Changed fold (FSH^+^/FSH^−^)
P12814	Alpha-actinin-1	ACTN1	116.17	5.25	1.60
P51636	Caveolin-2	CAV2	20.63	5.06	1.61
P02452	Collagen alpha-1(I) chain	COL1A1	153.17	5.6	2.11
P08123	Collagen alpha-2(I) chain	COL1A2	141.44	9.08	2.07
P02458	Collagen alpha-1(II) chain	COL2A1	158.37	6.58	4.00
P02461	Collagen alpha-1(III) chain	COL3A1	154.17	6.21	1.71
P02462	Collagen alpha-1(IV) chain	COL4A1	183.2	8.55	2.35
P08572	Collagen alpha-2(IV) chain	COL4A2	187.44	8.89	2.00
Q01955	Collagen alpha-3(IV) chain	COL4A3	183.7	9.28	2.58
P53420	Collagen alpha-4(IV) chain	COL4A4	185.01	8.9	4.48
Q14031	Collagen alpha-6(IV) chain	COL4A6	187.13	9.31	3.77
P05997	Collagen alpha-2(V) chain	COL5A2	160	6.07	2.06
P12109	Collagen alpha-1(VI) chain	COL6A1	122.21	5.26	1.88
P12110	Collagen alpha-2(VI) chain	COL6A2	122.08	5.85	1.82
P12111	Collagen alpha-3(VI) chain	COL6A3	381.6	6.26	1.96
O60610	Protein diaphanous homolog 1	DIAPH1	166.23	5.31	−1.69
P56199	Integrin alpha-1	ITGA1	149.03	5.91	1.74
P23229	Integrin alpha-6	ITGA6	144.45	6.21	2.31
P16144	Integrin beta-4	ITGB4	220.64	5.74	2.60
P25391	Laminin subunit alpha-1	LAMA1	379.1	5.93	2.73
P24043	Laminin subunit alpha-2	LAMA2	395.83	6.01	4.62
Q16787	Laminin subunit alpha-3	LAMA3	410.94	7.03	1.98
Q16363	Laminin subunit alpha-4	LAMA4	228.85	5.89	1.75
O15230	Laminin subunit alpha-5	LAMA5	426.69	6.66	2.35
P07942	Laminin subunit beta-1	LAMB1	224.86	4.83	1.88
P55268	Laminin subunit beta-2	LAMB2	210.77	6.07	2.95
P11047	Laminin subunit gamma-1	LAMC1	201.98	5.01	2.72
Q13753	Laminin subunit gamma-2	LAMC2	147.14	5.83	2.35
O14950	Myosin regulatory light chain 12B	MYL12B	23.49	4.71	1.89
P24844	Myosin regulatory light polypeptide 9	MYL9	23.31	4.8	1.79
P35442	Thrombospondin-2	THBS2	144.1	4.62	− 5.88
P24821	Tenascin	TNC	265.37	4.79	2.30
Q9UQP3	Tenascin-N	TNN	162.27	5.41	2.16
Q92752	Tenascin-R	TNR	159.6	4.71	− 2.00
P22105	Tenascin-X	TNXB	492.67	5.05	2.07

*DEP* differentially expressed protein, *MW* molecular weight, *FSH* follicle stimulating hormone

**Table 6:** DEPs involved in PI3K-Akt signaling pathway

Protein accession	Protein description	Gene name	MW (kDa)	calc. pI	Changed fold (FSH^+^/FSH^−^)
Q16363	Laminin subunit alpha-4	LAMA4	228.85	5.89	1.75
P02452	Collagen alpha-1(I) chain	COL1A1	153.17	5.6	2.11
P08123	Collagen alpha-2(I) chain	COL1A2	141.44	9.08	2.07
P02458	Collagen alpha-1(II) chain	COL2A1	158.37	6.58	4.00
P02461	Collagen alpha-1(III) chain	COL3A1	154.17	6.21	1.71
P02462	Collagen alpha-1(IV) chain	COL4A1	183.2	8.55	2.35
P08572	Collagen alpha-2(IV) chain	COL4A2	187.44	8.89	2.01
Q01955	Collagen alpha-3(IV) chain	COL4A3	183.7	9.28	2.58
P53420	Collagen alpha-4(IV) chain	COL4A4	185.01	8.9	4.48
Q14031	Collagen alpha-6(IV) chain	COL4A6	187.13	9.31	3.77
P05997	Collagen alpha-2(V) chain	COL5A2	160	6.07	2.06
P12109	Collagen alpha-1(VI) chain	COL6A1	122.21	5.26	1.88
P12110	Collagen alpha-2(VI) chain	COL6A2	122.08	5.85	1.82
P12111	Collagen alpha-3(VI) chain	COL6A3	381.6	6.26	1.96
P01241	Somatotropin	GH1	27.41	5.29	− 4.55
P06213	Insulin receptor	INSR	174.5	5.83	− 3.03
P56199	Integrin alpha-1	ITGA1	149.03	5.91	1.74
P23229	Integrin alpha-6	ITGA6	144.45	6.21	2.31
P16144	Integrin beta-4	ITGB4	220.64	5.74	2.60
P25391	Laminin subunit alpha-1	LAMA1	379.1	5.93	2.73
P24043	Laminin subunit alpha-2	LAMA2	395.83	6.01	4.62
Q16787	Laminin subunit alpha-3	LAMA3	410.94	7.03	1.98
O15230	Laminin subunit alpha-5	LAMA5	426.69	6.66	2.35
P07942	Laminin subunit beta-1	LAMB1	224.86	4.83	1.88
P55268	Laminin subunit beta-2	LAMB2	210.77	6.07	2.95
P11047	Laminin subunit gamma-1	LAMC1	201.98	5.01	2.72
Q13753	Laminin subunit gamma-2	LAMC2	147.14	5.83	2.35
P35558	Phosphoenolpyruvate carboxykinase, cytosolic [GTP]	PCK1	79.11	5.8	− 4.55
P01236	Prolactin	PRL	29.24	6.5	− 10.00
P35442	Thrombospondin-2	THBS2	144.1	4.62	− 5.88
P24821	Tenascin	TNC	265.37	4.79	2.30
Q9UQP3	Tenascin-N	TNN	162.27	5.41	2.16
Q92752	Tenascin-R	TNR	159.6	4.71	− 2.00
P22105	Tenascin-X	TNXB	492.67	5.05	2.07

*DEP* differentially expressed protein, *MW* molecular weight, *FSH* follicle stimulating hormone

#### ECM-receptor interaction pathway

ECM was a three-dimensional network of proteins, glycosaminoglycans, and other macromolecules, which was constantly undergoing a remodeling process. Specific interactions between cells and ECM were mediated by transmembrane molecules that were mainly integrins, and perhaps proteoglycans, CD36, or other cell surface components. These interactions lead to a direct or indirect control of cellular activities such as adhesion, migration, differentiation, proliferation, and apoptosis. This study found that integrins (ITGA1, ITGA6, and ITGB4), laminins (LAMA1, LAMA2, LAMA3, LAMA4, LAMA5 LAMB1, LAMB2, LAMC1, and LAMC2), and collagens (COL1A1, COL1A2, COL2A1, COL3A1, COL4A1, COL4A2, COL4A3, COL4A4, COL4A6, COL5A2, COL6A1, COL6A2, and COL6A3) were upregulated in the FSH-positive relative to negative NFPAs, and integrins were the key molecules in this pathway (Fig. 3, Table 4).

#### Focal adhesion pathway

Cell-matrix adhesions played significant roles in multiple biological processes, including cell motility, cell proliferation, cell differentiation, regulation of gene expression, and cell survival. Focal adhesions were the specialized structures to contact cell and ECM, where bundles of actin filaments were anchored to transmembrane receptors of the integrin family through a multi-molecule complex of junctional plaque proteins. Integrin signaling was dependent upon activities of non-receptor tyrosine kinase (FAK and Src proteins) and of the adaptor proteins (Src and Shc) of FAK to initiate downstream signaling events (Fig. 4). These signaling events were culminated in reorganization of actin cytoskeleton. In this pathway, integrins (ITGA1, ITGA6, and ITGB4), laminins (LAMA1, LAMA2, LAMA3, LAMA4, LAMA5, LAMB1, LAMB2, LAMC1, and LAMC2), and collagens (COL1A1, COL1A2, COL2A1, COL3A1, COL4A1, COL4A2, COL4A3, COL4A4, COL4A6, COL5A2, COL6A1, COL6A2, and COL6A3) were upregulated in FSH-positive NFPAs (Table 5), and FAK played key roles in this pathway. Also, FAK was associated with several signaling molecules, such as Src, Shc, p130Cas, Grb2, PI3k, Grb2, and paxillin, which enabled FAK to link both integrin receptors and non-integrin stimuli to intracellular signaling pathway.

#### PI3K-Akt signaling pathway

The phosphatidylinositol 3-kinase (PI3K)-Akt signaling pathway was activated by multiple cellular stimuli or toxic insults to regulate fundamental cellular functions, including transcription, translation, proliferation, growth, and survival. The bindings of growth factors to receptor tyrosine kinase (RTK), and cytokines/hormones to G protein-coupled receptors (GPCR) stimulated class IA PI3K isoforms, or the bindings of ECM components to integrins stimulated class IB PI3K isoforms (Fig. 5). PI3K phosphorylated phosphatidylinositol 4,5-bisphosphate (PIP2) to produce phosphatidylinositol 3,4,5-trisphosphate (PIP3) at the cell membrane. PIP3 in turn served as a second messenger to activate AKT. The activated AKT phosphorylated multiple substrates to control various cellular processes, including apoptosis, protein synthesis, metabolism, and cell cycle. PI3K and AKT played important roles in PI3K-Akt signaling pathway. In this pathway, integrins (ITGA1, ITGA4, and ITGB4), laminins (LAMA1, LAMA2, LAMA3, LAMA4, and LAMA5), and collagens (COL3A1, COL4A1, COL1A2, COL2A1, COL4A3, COL6A1, COL6A2, COL6A3, COL4A6, COL1A1, COL5A2, COL4A2, and COL4A4) were upregulated (Table 6).

### DEG profiling and pathway networks between invasive and non-invasive NFPAs

A total of 898 DEGs were obtained between invasive and non-invasive NFPAs with transcriptomic dataset GSE 51618 of NFPAs from GEO database (Supplemental Table 2). KEGG pathway enrichment analysis of those 898 DEGs revealed 16 statistically significant pathways (Supplemental Table 3; Supplemental Fig. 2), including focal adhesion pathway, VEGF signaling pathway, MAPK signaling pathway, Rap1 signaling pathway, Gap junction, phagosome, GnRH signaling pathway, T cell receptor signaling pathway, and ErbB signaling pathway. These signaling pathways were obviously associated with pituitary invasiveness and aggressiveness. Interestedly, focal adhesion pathway was both identified with KEGG pathway analyses of 898 DEG data between invasive vs. non-invasive NFPAs, and of 594 DEP data between FSH-positive vs. negative NFPAs. This result further supported FSH-related invasive molecular characteristics in FSH-positive NFPAs compared to FSH-negative NFPAs. Furthermore, overlapping analysis of 898 invasiveness-related DEGs and 594 FSH-related DEPs found 45 overlapped molecules that were changed in both mRNA and protein levels in NFPAs (Table 7), which were molecular profiling to link FSH-positive expression and invasiveness in NFPAs.

**Table 7:** Overlapped molecules between 594 DEPs in FSH-positive vs. negative NFPAs and 898 DEGs in invasive vs. non-invasive NFPAs from GEO database

Gene	DEPs (FSH^+^ vs. FSH^−^)		DEGs (invasive vs. non-invasive)	
	FC	adj.P.Val	FC	adj.P.Val
FSTL4	0.32	2.07E-05	0.22	2.49E-02
SLC27A2	0.38	2.97E-06	0.09	6.74E-03
HP	0.41	6.48E-06	0.06	4.60E-02
IL13RA2	0.42	2.73E-06	0.01	7.67E-03
DPP6	0.44	4.26E-06	0.02	7.63E-03
CRABP2	0.49	7.70E-06	0.08	2.24E-02
NPTX2	0.53	1.11E-04	0.08	1.99E-02
ALCAM	0.61	9.00E-05	0.08	3.65E-02
GATM	1.60	1.11E-05	6.85	2.35E-02
FARP1	1.64	4.28E-05	2.71	4.90E-02
PALLD	1.65	7.62E-07	6.03	2.16E-02
CLU	1.65	1.36E-05	2.84	3.46E-02
LIMA1	1.66	7.57E-05	2.98	5.30E-03
UACA	1.73	6.42E-05	4.79	3.84E-02
SCD	2.08	1.84E-04	8.49	3.55E-02
BAG3	2.10	1.87E-04	4.13	4.91E-02
PLEC	2.19	4.63E-07	2.03	4.61E-02
PPBP	2.24	5.97E-06	6.73	1.39E-02
ITGA6	2.31	2.43E-07	3.81	3.77E-02
NDNF	0.25	9.52E-07	62.59	7.13E-04
CALB1	0.37	1.68E-05	491.00	3.14E-05
HARS	0.43	2.52E-07	2.05	2.77E-02
METTL7A	0.49	9.67E-05	12.76	9.11E-03
NEFL	0.54	5.71E-06	126.46	9.62E-04
EZR	0.54	2.95E-06	2.52	2.98E-02
ANKRD24	0.55	1.81E-05	19.85	8.68E-04
ACSL1	0.55	2.69E-05	3.89	3.41E-02
NSF	0.58	1.28E-06	2.32	3.88E-02
NR3C1	0.60	1.28E-05	4.09	1.65E-02
ACTN1	1.60	1.09E-05	0.29	4.35E-02
TP53I11	1.61	4.34E-03	0.40	4.35E-02
SRCIN1	1.66	6.13E-06	0.15	6.06E-03
PHYH	1.74	1.20E-05	0.46	2.58E-02
IQGAP2	1.75	9.79E-05	0.12	1.10E-02
SPATA20	1.77	2.78E-06	0.36	3.87E-02
EPB41L1	1.83	2.74E-04	0.42	1.35E-02
KIAA1671	2.01	1.27E-05	0.48	2.91E-02
MAOB	2.17	3.26E-06	0.18	3.20E-03
GAL3ST3	2.17	8.19E-05	0.22	5.46E-03
CYP11A1	2.23	4.15E-05	0.20	3.06E-02
COL22A1	2.54	8.36E-08	0.02	9.13E-03
NECAB1	2.66	1.62E-05	0.37	4.26E-02
LAMA1	2.73	3.25E-05	0.09	2.67E-02
GPC4	3.68	3.77E-06	0.07	2.70E-02
LAMA2	4.62	6.34E-06	0.09	3.18E-02

### PPI network construction and hub-molecule selection

**Fig. 6:**
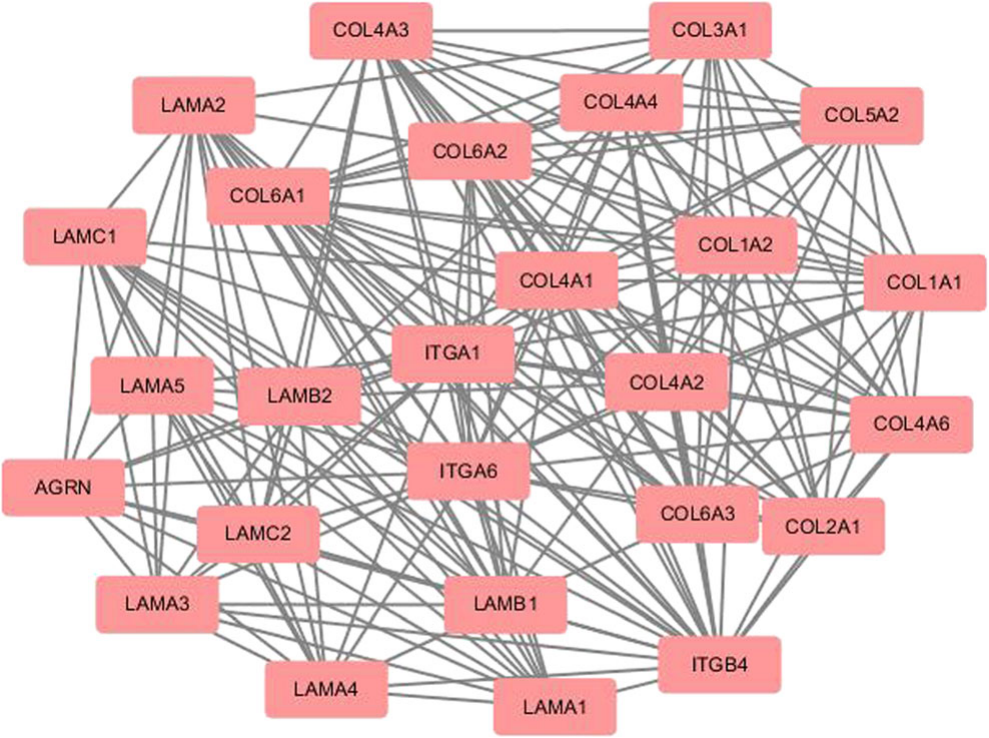
The most significant module of PPI network of DEPs involved in ECM-receptor interaction, focal adhesion, and PI3K-Akt signaling pathway. The most significant module was obtained from PPI network with 26 nodes, and 204 edges. Red: upregulated DEPs

The PPI network of DEPs that were involved in ECM-receptor pathway, focal adhesion, and PI3K-Akt pathway was constructed, and the most significant module was identified with Cytoscape (Fig. 6). A total of 26 molecules were identified as hub-molecules with degrees ≥ 10, including AGRN, COL1A1, COL1A2, COL2A1, COL3A1, COL4A1, COL4A2, COL4A3, COL4A4, COL4A6, COL5A2, COL6A1, COL6A2, COL6A3, LAMA1, LAMA2, LAMA3, LAMA4, LAMA5, LAMB1, LAMB2, LAMC1, LAMC2, ITGA1, ITGA6, and ITGB4. Those 26 hub-molecules were all upregulated DEPs in FSH-positive relative to negative NFPAs. Most of these hub-molecules belonged to ECM components and integrins. Tumor cells invading into the surrounding ECM network system were mainly mediated by integrins and other cell adhesion molecules. Therefore, those 26 hub-molecule**s** (upregulated DEPs) were involved in tumor invasiveness and aggressiveness.

### Validation of DEPs and signaling pathways in FSH-positive vs. negative NFPAs

**Fig. 7:**
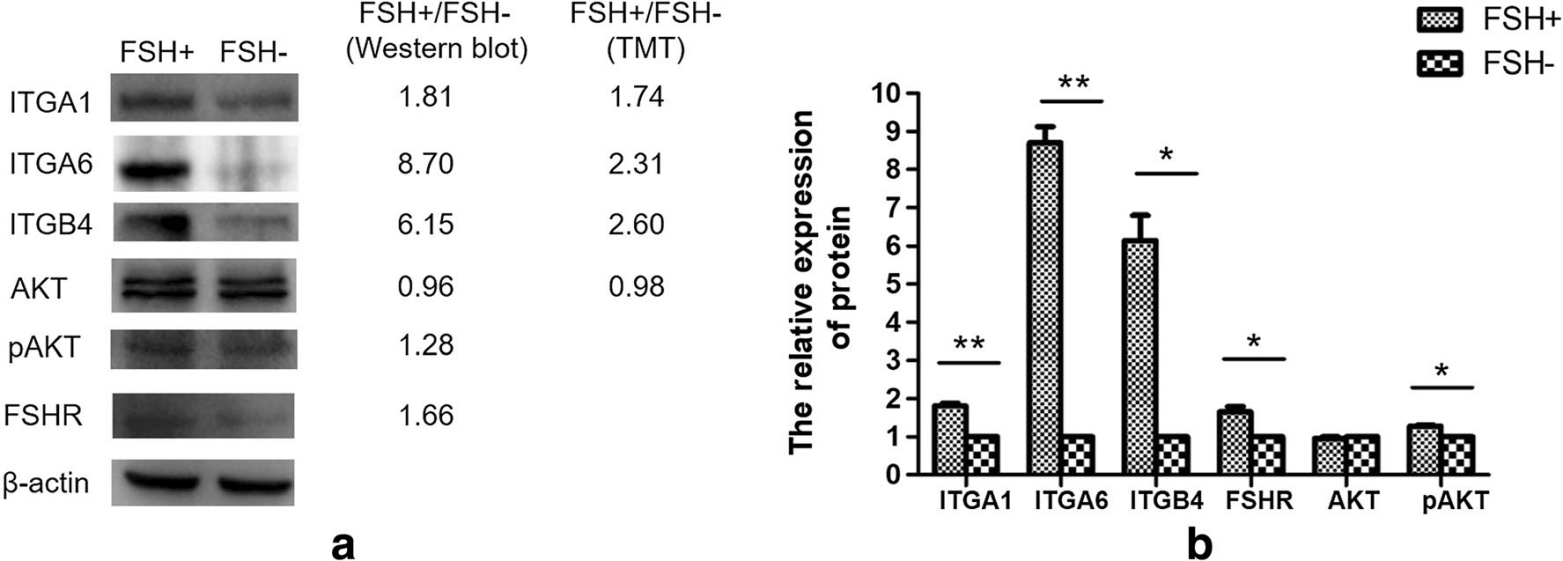
Western blot of DEPs and relatively quantitative expressions between FSH^+^ and FSH^−^ NFPAs**. a** Western blot of ITGA1, ITGA6, ITGB4, AKT, pAKT, and FSHR. **b** The relative expression of proteins in FSH^+^ and FSH^−^ NFPAs. *n* = 3. *p < 0.05; **p < 0.01

To validate DEPs from TMT-based quantitative proteomics, Western blotting analyses of three DEPs (ITGA1, ITGA6, and ITGB4) revealed that those three proteins were significantly upregulated in FSH-positive relative to negative NFPAs (Fig. 7), and the changed-fold of ITGA1, ITGA6, and ITGB4 was 1.81, 8.70, and 6.15 in FSH-positive relative to negative NFPAs, respectively. The Western blot results were consistent with the TMT-based quantitative proteomics.

Study found that FSHR in tumor cells was overexpressed in aggressive PAs compared to non-aggressive PAs [26]. In this study, Western blot analysis revealed that FSHR was significantly upregulated in FSH-positive relative to negative NFPAs (*p* < 0.05) (Fig. 7). Moreover, the expression and phosphorylation of AKT in PI3K-Akt signaling pathway were detected with Western blotting between FSH-positive vs. negative NFPAs. The Western blot result showed that no significant difference was found in the protein expression level of AKT between FSH-positive vs. negative NFPAs (ratio of FSH^+^/FSH^−^ = 0.96; *p* > 0.05), which is consistent with TMT-based quantitative proteomics (ratio of FSH^+^/FSH^−^ = 0.98; *p* > 0.05). However, phosphorylation level of AKT was significantly increased in FSH-positive vs. negative NFPAs (*p* < 0.05) (Fig. 7), which might be an important factor to activate PI3K-Akt pathway in FSH-positive NFPAs.

## Discussion

NFPAs were highly heterogeneous, including different hormone-expression subtypes; and were commonly considered as benign tumors. However, they might infiltrate into surrounding tissues including the dura mater. Local invasion and aggressiveness was observed with the naked eye in approximately 40% PAs, and microscopically confirmed local invasion and aggressiveness in 80% PAs [30, 31]. Actually, invasiveness and aggressiveness were not synonymous [32]. Aggressive PAs had two important characteristics: First, aggressiveness means invasively expanding into surrounding tissue structures, especially infiltrating the precise anatomical structure such as bone invasion. Second, aggressiveness was characterized with postoperative recurrent behaviors of such tumors, and usually exhibited resistance to conventional therapies. Aggressive PAs had greater chance to progress to malignant pituitary carcinomas that were characterized with cerebrospinal or systemic metastases. Therefore, aggressive PAs were within the range from benign PAs to malignant pituitary carcinomas. The extensive local invasion and aggressiveness made NFPA difficult to completely remove tumor tissues by neurosurgery and left patients at risk for recurrence. It is necessary to stratify such type of PA patients, reveal their invasive and aggressive mechanisms, and discover the effective biomarkers so as to provide an effective prediction, prognosis, targeted therapy, and personalized treatment strategy. It was reported that FSHR expression in PAs was a marker of aggressiveness [26], and FSHR must bind with FSH to exert their biological roles. Moreover, FSH-positive expressed NFPAs were one of NFPA subtypes [20, 33]. It raised a clinical question whether the FSH-positive NFPAs have more invasive or aggressive molecular characteristics than FSH-negative NFPAs. In other words, if the invasive or aggressive molecular characteristics can be determined in FSH-positive NFPAs, then in the future when one meet this type of FSH-positive NFPA patient and the corresponding molecular changes, whatever the tumor size and already damage to tumor-surrounding structures, then this type of NFPA patients should be considered for personalized, and extra treatment strategy after neurosurgery. This study, for the first time, analyzed DEP profiling between FSH-positive vs. negative NFPA tissues with TMT-based quantitative proteomics, pathway network analysis, and in combination with an analysis of DEG data between invasive and non-invasive NFPAs from GEO database. Comprehensive analysis of all data revealed that signaling pathways (ECM-receptor interaction pathway, focal adhesion pathway, and PI3K-Akt pathway) and key molecules (ITGA1, ITGA6, ITGB4, FSHR, and pAKT) were more active in FSH-positive relative to negative NFPAs, and that a set of invasiveness-related molecules including 11 upregulated DEPs (ITGA6, FARP1, PALLD, PPBP, LIMA1, SCD, UACA, BAG3, CLU, PLEC, and GATM) and 8 downregulated DEPs (ALCAM, HP, FSTL4, IL13RA2, NPTX2, DPP6, CRABP2, and SLC27A2) were presented in FSH-positive NFPAs.

### FSH-related signaling pathway alterations in NFPAs

ECM-receptor interaction, focal adhesion, and PI3K-Akt pathways were enriched with DEP data between FSH-positive and negative NFPAs, which were reported to associate with tumorigenesis, invasiveness, aggressiveness, and progression [34–36]. Also, focal adhesion pathway was also enriched with DEG data between invasive and non-invasive NFPAs. More interesting thing was that focal adhesion pathway functioned in the upstream of PI3K-Akt pathway and in the downstream of ECM-receptor pathway. Therefore, those three pathways were actually regulated mutually to act in FSH-positive NFPAs.

Each component of ECM such as fibronectins, laminins, and collagens played specific roles in cell proliferation, differentiation, morphogenesis, and hormone production [37–41]. Integrins were the major surface receptors of ECM, which played central roles in the ECM pathways. Many ECM components were able to bind to different integrins that included different α and β subunits [42]. Integrins and ECM took part not only in normal physiological functions but also in tumorigenesis [43]. ECM played a vital role in the process of cancer, and mediated tumor cell invasion and metastasis [44]. As one of the major components of ECM, laminins were correlated with a variety of tumor initiation and progression. For example, laminins regulated ovarian cancer cell proliferation [45], thyroid carcinoma cell growth and differentiation [46], cell differentiation in colon cancer cells [47, 48], and invasion of highly aggressive MDA-MB-231 breast cancer cells [49]. In this study, a series of laminins including LAMA1, LAMA2, LAMA3, LAMA4, LAMA5 LAMB1, LAMB2, LAMC1, and LAMC2 were upregulated in invasive NFPAs. They might be associated with a more invasive and aggressive NFPAs. Integrins were the major receptors that connected cells to the surrounding ECM and mediated cell-cell adhesions. Changes in tumor cell adhesions affected growth and progression of a tumor. Several integrin subunits showed different expression levels between normal and adenomatous cells [50], which suggested a possible role of integrins in PAs. Integrins were likely to facilitate tumor angiogenesis. Angiogenesis was required both during initial tumor invasion and growth, and during metastatic spread [51]. Angiogenic factors, such as bFGF and VEGF, enhanced the expressions and activities of endothelial integrins [52, 53]. This present study found that VEGF signaling pathway was enriched with DEG data between invasive and non-invasive NFPAs. Integrin α6 and β4 signaling promoted the onset of the invasive phase of pathological angiogenesis [51]. Integrin α6 (ITGA6) promoted cell migration during embryonic development [54]. It has been reported that Twist2 promoted kidney cancer cell proliferation and invasion by upregulating ITGA6 expression in the ECM-receptor interaction pathway [34]. Furthermore, *Clonorchis sinensis* excretory-secretory products promoted the migration and invasion of cholangiocarcinoma cells by activating the integrin β4–FAK/Src signaling pathway [55]. The engagement of integrin α1 with functional molecular scaffolds using FAK/src and p130Csa/JNK was related to colon cancer cell invasion [56]. It was clear that ECM-receptor interaction pathway obviously contributed to tumor invasiveness and aggressiveness.

Integrin receptors initiated signal transduction events that affected cell growth. However, they did not possess catalytic activities. The signals initiated by ECM-integrin interactions were transduced into cells through activating integrin-associated proteins. FAK was colocalized with integrin receptors at cell-substratum contact sites named focal adhesion [57]. Thus focal adhesion pathway played a vital role in linking integrin receptor to intracellular signaling pathways. Study found that focal adhesion pathway was related to tumor invasion and metastasis [35]. FAK was reported to be overexpressed in a variety of human tumors, and FAK overexpression might lead to an invasive potential for a variety of epithelial and mesenchymal tumor types [57]. The elevation of FAK protein levels was related to the invasive capacity in colon cancers, breast cancers, and oral cancers [58]. Immunohistochemistry results showed that FAK was expressed in 73.5% (36/49) PA cases, and their expression levels were highly correlated with tumor invasiveness [59]. Study found that microRNA-218 inhibited cell migration and invasion in renal cell carcinoma through upregulating genes in focal adhesion pathway [35]. In this study, FAK was not a DEP between FSH-positive and negative NFPAs. However, a series of proteins in focal adhesion pathway were upregulated in FSH-positive NFPAs, which might lead to more invasive and aggressive capabilities of FSH+ NFPAs.

The PI3K-Akt pathway was recognized as a key pathway involved in tumor cell migration and invasion [36, 60, 61]. The Akt kinase was activated by PI3K, and was dysregulated in a variety of tumors [62]. Akt was identified as an important regulator of cell proliferation, tumorigenesis, and apoptosis. Some studies proved that Akt activation was related to several tumor invasion [62]. Blocking PI3K-Akt pathway resulted in decreased invasive ability of cancer cells [63]. Activation of Akt was detected mostly in the invasive carcinomas [64]. Our study found the phosphorylation level of AKT was significantly increased in FSH-positive relative to negative NFPAs, which evidenced that over-activation of PI3K-Akt pathway in FSH-positive relative to negative NFPAs.

### FSH-related hub-molecules involved in NFPAs

ECM-receptor interaction, focal adhesion, and PI3K-Akt pathways were related to tumorigenesis, invasiveness, aggressiveness, and progression. In order to seek out the hub-molecules involved in progression of FSH-positive NFPAs, the PPI network of DEPs involved in those three pathways was constructed, and 26 significant hub-molecules were obtained with Cytoscape, and were all upregulated DEPs in FSH-positive relative to negative NFPAs. Most significant hub-molecules were mainly ECM components and integrins. ECM components played a role by binding to their receptors, mainly integrins. Three upregulated integrin components (ITGA1, ITGA6, and ITGB4) from TMT-based quantitative proteomics were also confirmed with Western blotting in FSH**-**positive relative to negative NFPAs. Akt was the key molecule in PI3K-Atk pathway, its expression and phosphorylation levels were detected with Western blot in FSH-positive relative to negative NFPAs. The expressions of ITGA1, ITGA6, ITGB4, and phosphorylated Akt (pAKT) were significantly higher in FSH-positive relative to negative NFPAs. All these results supported the more invasive and aggressive characteristics in FSH-positive relative to negative NFPAs. Moreover, the expression of FSHR was also significantly higher in FSH-positive relative to negative NFPAs. Study found that the incidence of FSHR expression was significantly higher in aggressive (68%) than in non-aggressive PAs (12%) [26]. Those findings clearly demonstrated that ITGA1, ITGA6, ITGB4, pAKT, and FSHR were significantly associated with the invasiveness, aggressiveness, and progression of FSH**-**positive relative to negative NFPAs.

Furthermore, overlapping analysis of 594 DEP data between FSH-positive and negative NFPAs and 898 DEG data between invasive and non-invasive NFPAs from GEO database revealed 45 overlapped molecules, including 11 upregulated DEPs (ITGA6, FARP1, PALLD, PPBP, LIMA1, SCD, UACA, BAG3, CLU, PLEC, and GATM) that were also upregulated genes in invasive NFPAs, and 8 downregulated DEPs (ALCAM, HP, FSTL4, IL13RA2, NPTX2, DPP6, CRABP2, and SLC27A2) that were also downregulated genes in invasive NFPAs. Those DEPs/DEGs formed the molecule alteration profiles of invasive characteristics in FSH-positive NFPAs.

## Conclusions and expert recommendation

TMT-based quantitative proteomics was an effective method, and identified the first large-scale DEP profile (*n* = 594 DEPs) in FSH-positive relative to negative NFPAs. GO and KEGG pathway enrichment analyses of those DEP data revealed multiple altered molecular characteristics in FSH-positive vs. negative NFPAs. Three pathways (ECM-receptor interaction pathway, focal adhesion pathway, and PI3K-Akt signaling pathway) involved in DEPs were all associated with tumorigenesis, invasiveness, aggressiveness, and progression. Most DEPs in those three pathways were upregulated in FSH-positive vs. negative NFPAs. It demonstrated that FSH-positive NFPAs had higher invasive and aggressive capabilities than FSH-negative NFPAs. Moreover, an analysis of DEG data between invasive and non-invasive NFPAs from GEO database also revealed focal adhesion pathway to significantly associate with invasive and aggressive characteristics. Focal adhesion pathway functioned in the downstream of ECM-receptor interaction pathway, and in the upstream of PI3K-Akt pathway, which clearly demonstrated that those three pathways were actually interacting mutually together in FSH-positive NFPAs. PPI analysis of DEPs from those three pathways revealed 26 significant hub-molecules (upregulated DEPs) including ITGA1, ITGA6, and ITGB4. Furthermore, Western blot analysis confirmed the upregulated expressions of ITGA1, ITGA6, ITGB4, pAKT, and FSHR in FSH**-**positive relative to negative NFPAs, which also confirmed the over-activations of FSH-related pathways (ECM-receptor interaction, focal adhesion, and PI3K-Akt signaling pathways) in FSH-positive relative to negative NFPAs. Furthermore, overlapping analysis of 594 DEP data between FSH-positive and negative NFPAs and 898 DEG data between invasive and non-invasive NFPAs revealed a set (*n* = 45) of invasiveness-relative DEPs in FSH-positive NFPAs.

These findings clearly revealed FSH-related molecular characterizations in FSH-positive NFPAs, demonstrated that FSH-positive NFPAs had more invasive and aggressive capabilities than FSH-negative NFPAs, and that ITGA1, ITGA6, ITGB4, pAKT, FSHR, and 45 overlapped DEP/DEG molecules were potential biomarkers to reveal the invasiveness-related molecular characteristics of FSH-positive NFPAs, which benefit for patient stratification, prognostic assessment, targeted therapy, and personalized treatment of NFPAs, and provided the scientific evidence for in-depth investigation of the roles of FSH in NFPAs.

These exciting findings help ones to in-depth understand molecular characteristics, especially its invasiveness-related molecular characteristics of FSH-positive NFPAs, which exactly helps one to resolve the challenging clinical problem—invasiveness and aggressiveness in NFPA patients. However, ones must also realize that this study is still in the “pilot study” stage with a small sample size in quantitative proteomics analysis between FSH-positive and negative NFPAs (*n* = 3 vs. 3), followed by validation with Western blot between FSH-positive and negative NFPAs (*n* = 4 vs. 5); and in comparative transcriptomics analysis between invasive and non-invasive NFPAs (*n* = 3 vs. 4). A significantly expanded sample size will be necessary to translate these scientific findings into clinical applications in the future.

We recommend one to emphasize insights into proteomic variations and transcriptomic variations in specific NFPA subtype research and clinical practice for patient stratification, prognostic assessment, and personalized treatment in future NFPA care [65, 66, 73]. Here, one must realize that NFPAs are highly heterogeneous, especially present in different hormone subtypes of NFPAs [19, 20]. Clarification of molecular characteristics of each hormone subtype of NFPAs will significantly benefit the understanding of its specific molecular mechanism and discovery of effective biomarkers for effective prognostic assessment and personalized treatment. This study revealed the FSH-positive related molecular characteristics—especially its invasion-related molecular characteristics—in FSH-positive NFPAs, which provided the scientific data for effectively prognostic assessment and personalized treatment of FSH-positive NFPA patients. Moreover, this study opened a new window and strategy to study the systematically molecular alterations in the level of multiomics [67–70] to effectively stratify patients for effectively predictive, preventive, and personalized management of different subtypes of NFPA patients.

## Electronic supplementary material

ESM 1 (PPT 1236 kb)

ESM 2 (PPT 1366 kb)

ESM 3 (XLS 152 kb)

ESM 4 (XLS 174 kb)

ESM 5 (XLS 33 kb)

## Abbreviations

ACTH: adrenocorticotropic hormone
AGC: automatic gain control
ARVC: arrhythmogenic right ventricular cardiomyopathy
BCA: bicin-choninic Acid
BP: biological processes
ACN: acetonitrile.
CC: cellular compartment
CV: coefficient of variation
DEG: differentially expressed genes
DEP: differentially expressed protein
DTT: dithiothreitol
ECM: excetral cellular matrix
EDTA: ethylene diamine tetraacetic acid
FAK: focal adhesion kinase
FPAs: functional pituitary adenomas
FSH: follicle stimulating hormone
FSHR: follicle-stimulating hormone receptor
GEO: gene expression omnibus
GO: gene ontology
GPCR: G protein-coupled receptors
HCD: high energy collision dissociation
hGH: human growth hormone
HPLC: high-performance liquid chromatography
KEGG: Kyoto Encyclopedia of Genes and Genomes
LH: luteinizing hormone
MF: molecular functions
MS: mass spectrometry
MS/MS: tandem mass spectrometry
MW: molecular weight
NFPAs: non-functional pituitary adenomas
NSI: neutral spray ionization
PAs: pituitary adenomas
PIP3: phosphatidylinositol 3,4,5-trisphosphate
PRL: prolactin
Pyk2: proline-rich tyrosine kinase 2
TCA: trichloroacetic acid
TEAB: tetraethyl ammonium bromide
TSH: thyroid stimulating hormone
UPLC: ultra performance liquid chromatography
