# Supplementary material for: TMT-based quantitative proteomics revealed follicle-stimulating hormone (FSH)-related molecular characterizations for potentially prognostic assessment and personalized treatment of FSH-positive non-functional pituitary adenomas
Source: EPMA J. 2019 Aug 29;10(4):395–414. doi: 10.1007/s13167-019-00187-w (PMC6882982; doi:10.1007/s13167-019-00187-w)
Supplement: Supplementary file 1 — (PPT 1236 kb) [file 13167_2019_187_MOESM1_ESM.ppt]

## Slide 1
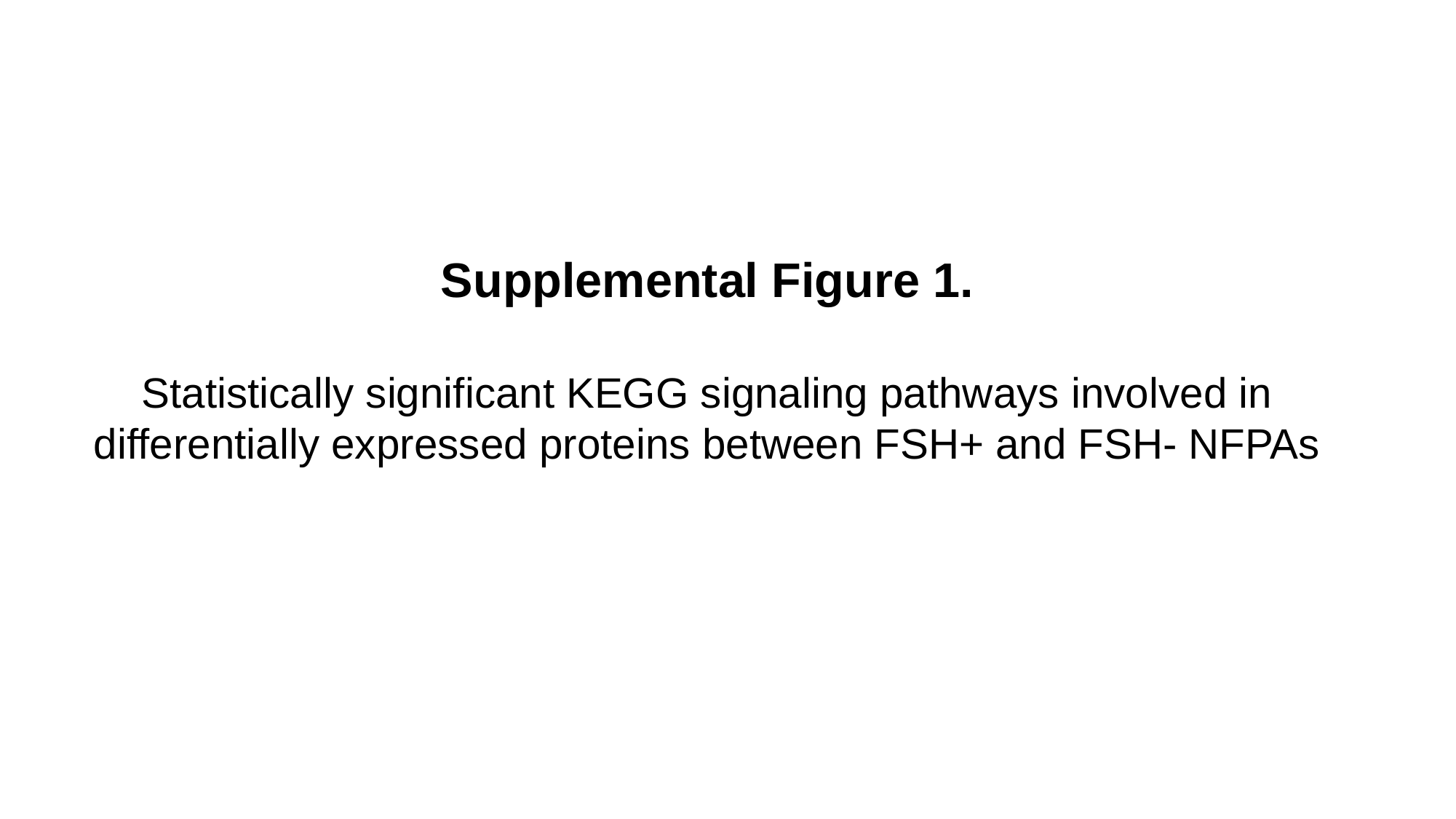

Supplemental Figure 1.
Statistically significant KEGG signaling pathways involved in differentially expressed proteins between FSH+ and FSH- NFPAs

## Slide 2
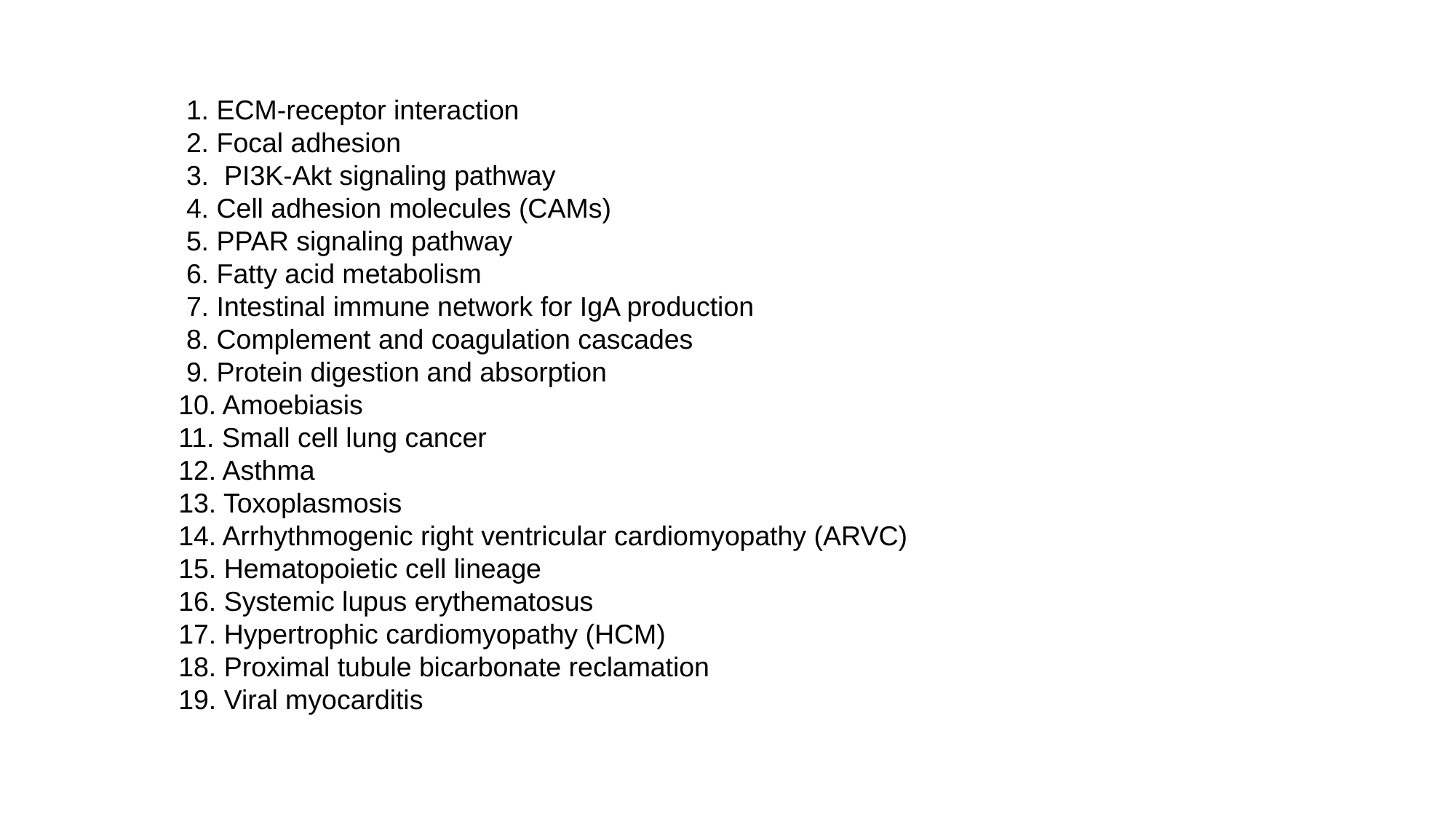

1. ECM-receptor interaction
 2. Focal adhesion
 3. PI3K-Akt signaling pathway
 4. Cell adhesion molecules (CAMs)
 5. PPAR signaling pathway
 6. Fatty acid metabolism
 7. Intestinal immune network for IgA production
 8. Complement and coagulation cascades
 9. Protein digestion and absorption
10. Amoebiasis
11. Small cell lung cancer
12. Asthma
13. Toxoplasmosis
14. Arrhythmogenic right ventricular cardiomyopathy (ARVC)
15. Hematopoietic cell lineage
16. Systemic lupus erythematosus
17. Hypertrophic cardiomyopathy (HCM)
18. Proximal tubule bicarbonate reclamation
19. Viral myocarditis

## Slide 3
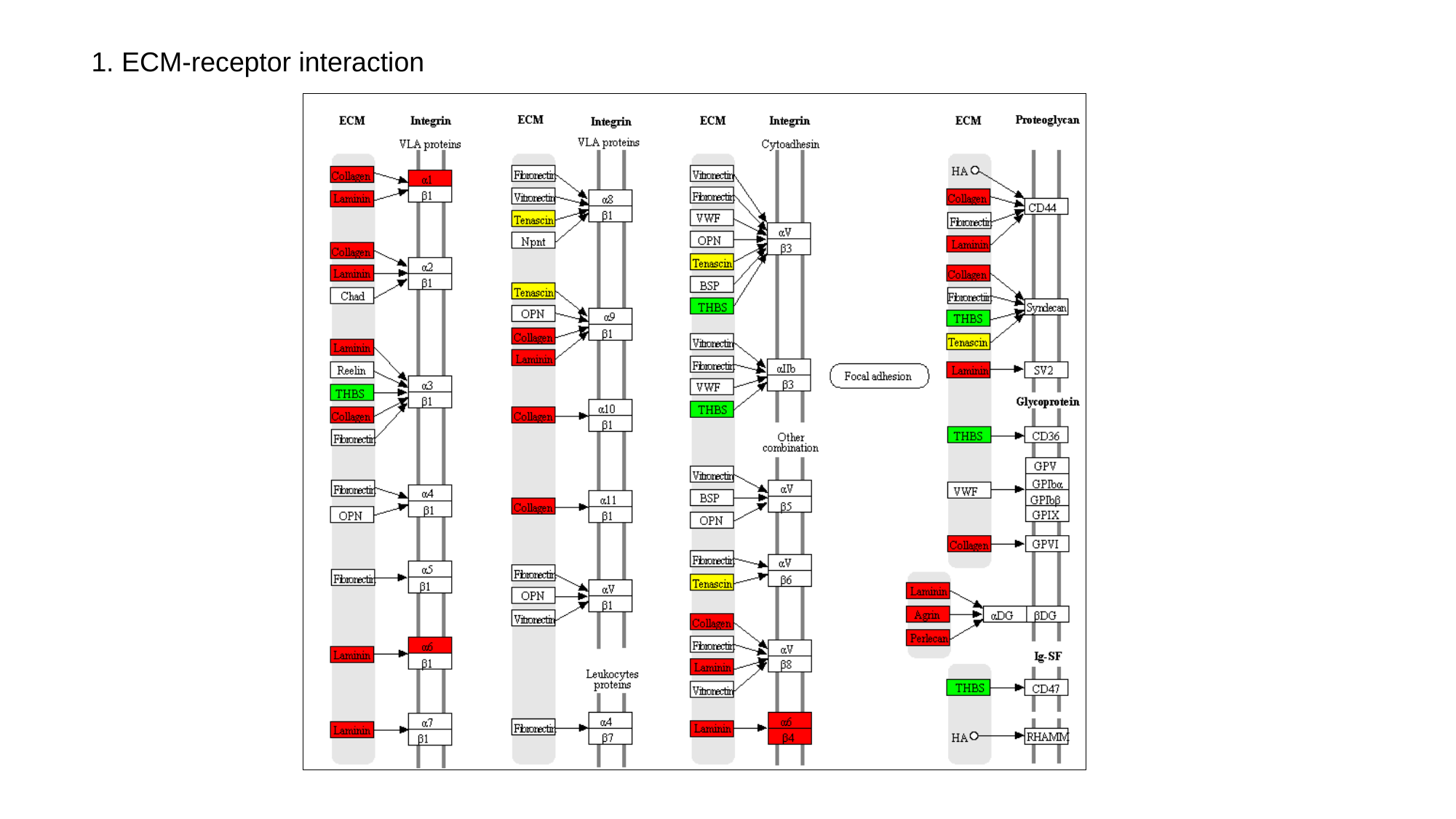

1. ECM-receptor interaction

## Slide 4
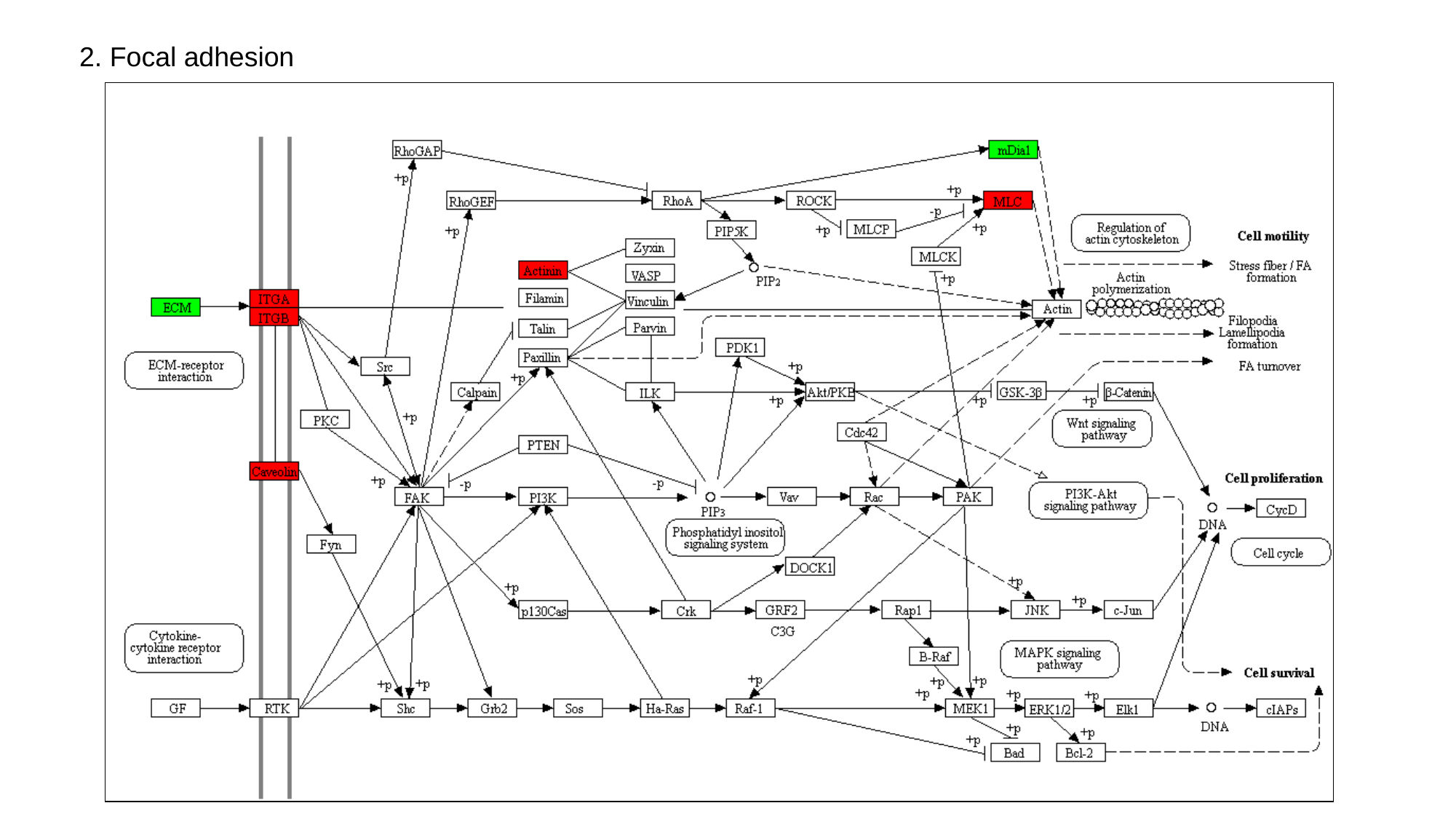

2. Focal adhesion

## Slide 5
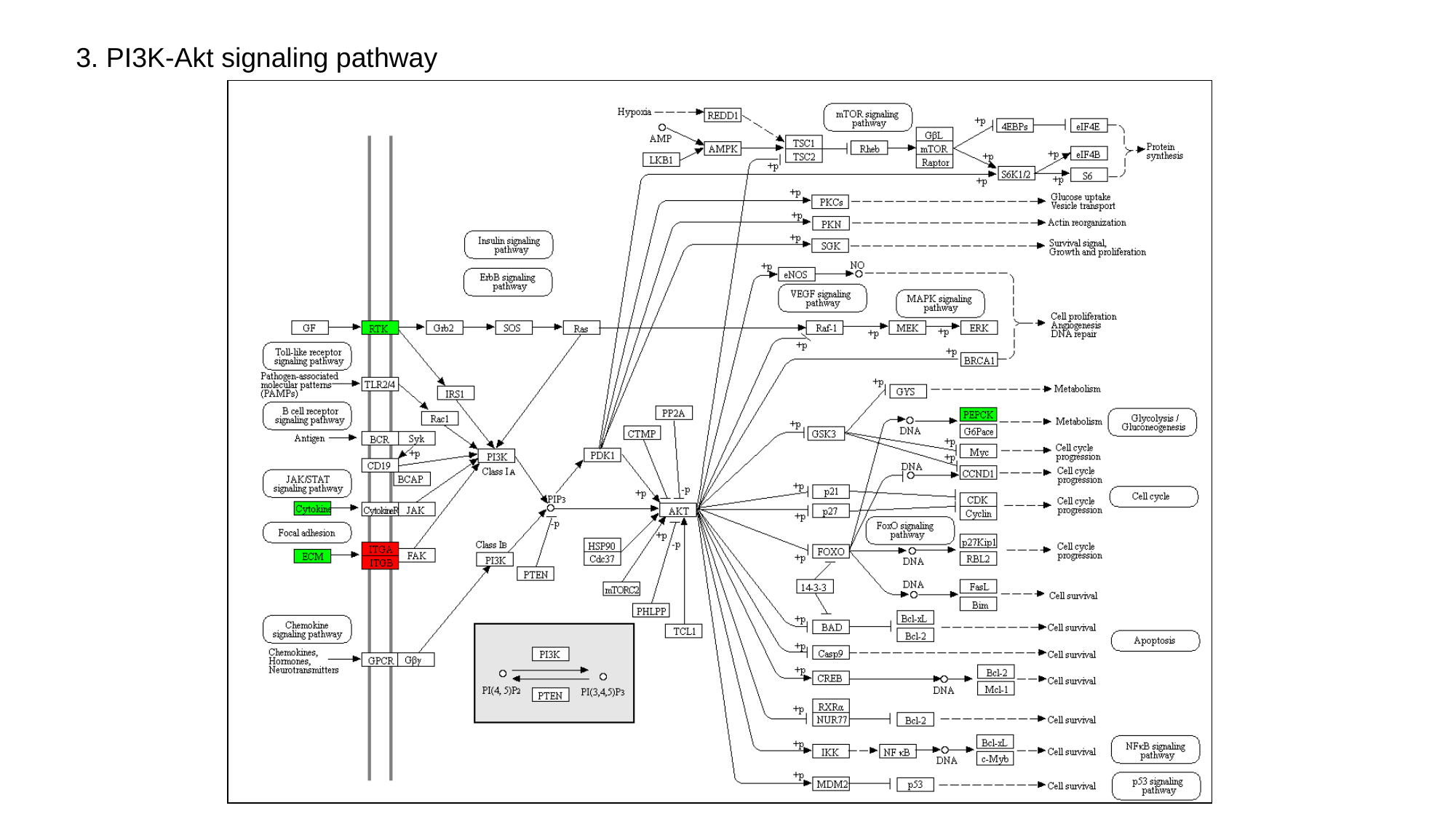

3. PI3K-Akt signaling pathway

## Slide 6
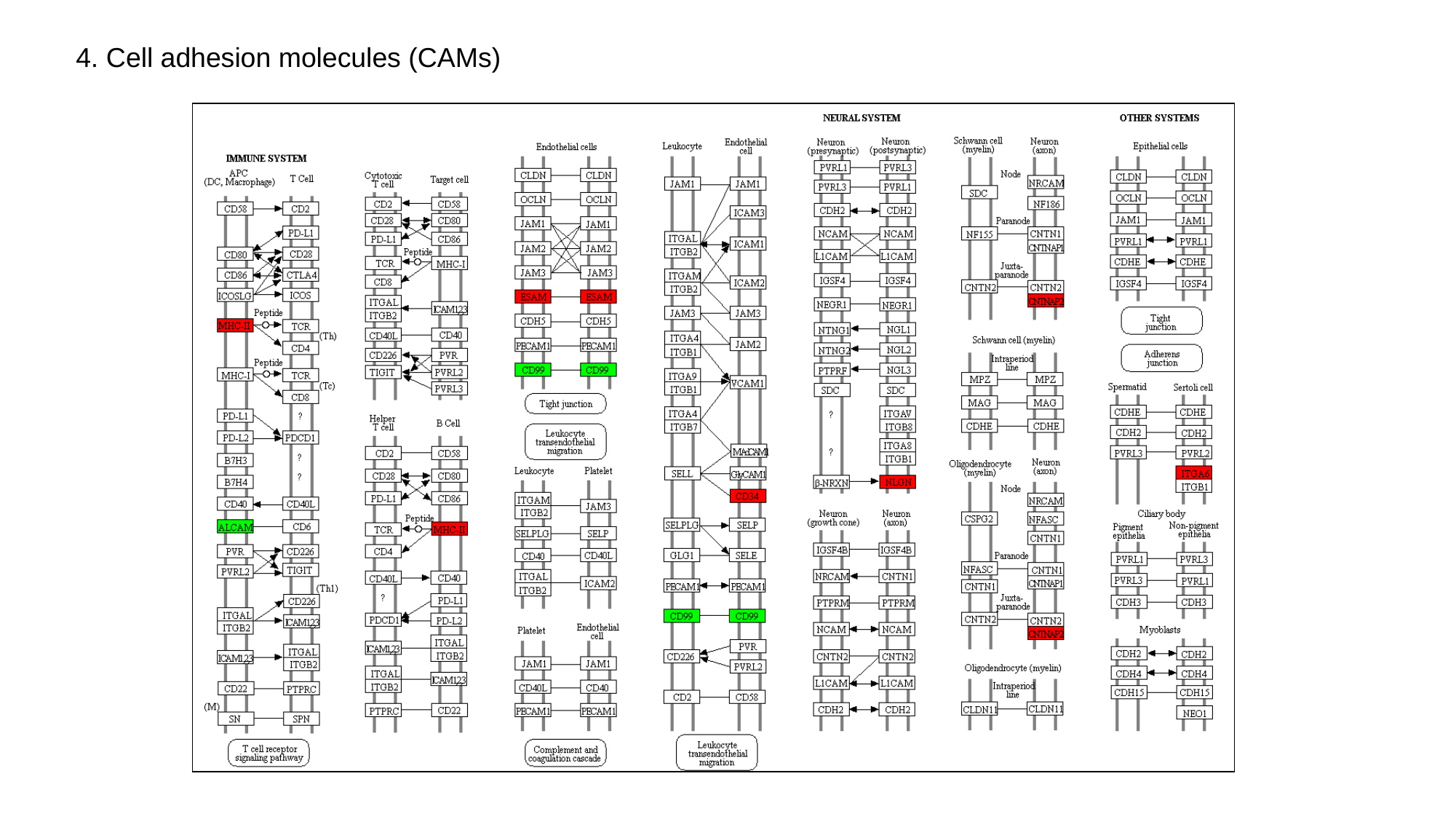

4. Cell adhesion molecules (CAMs)

## Slide 7
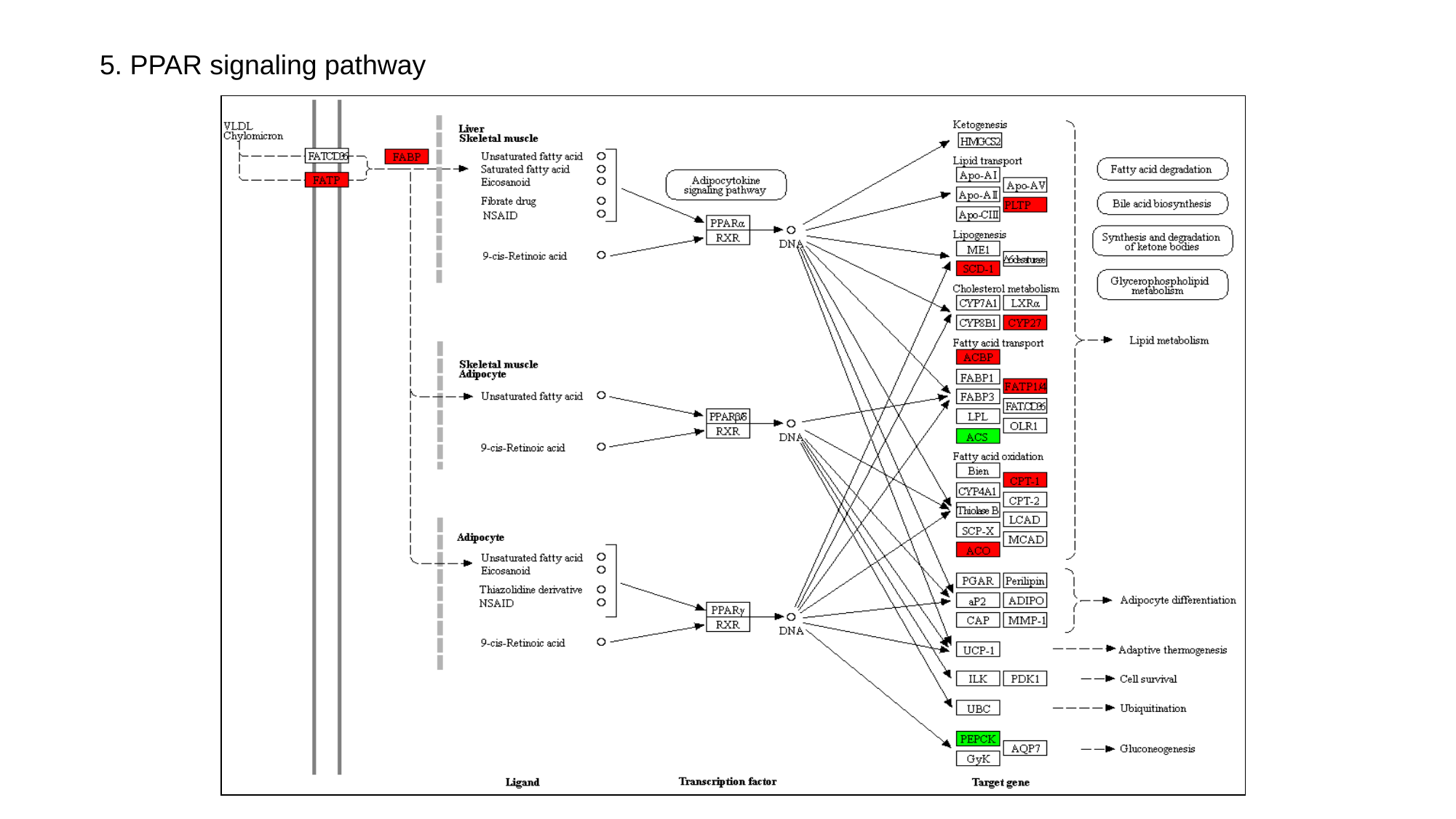

5. PPAR signaling pathway

## Slide 8
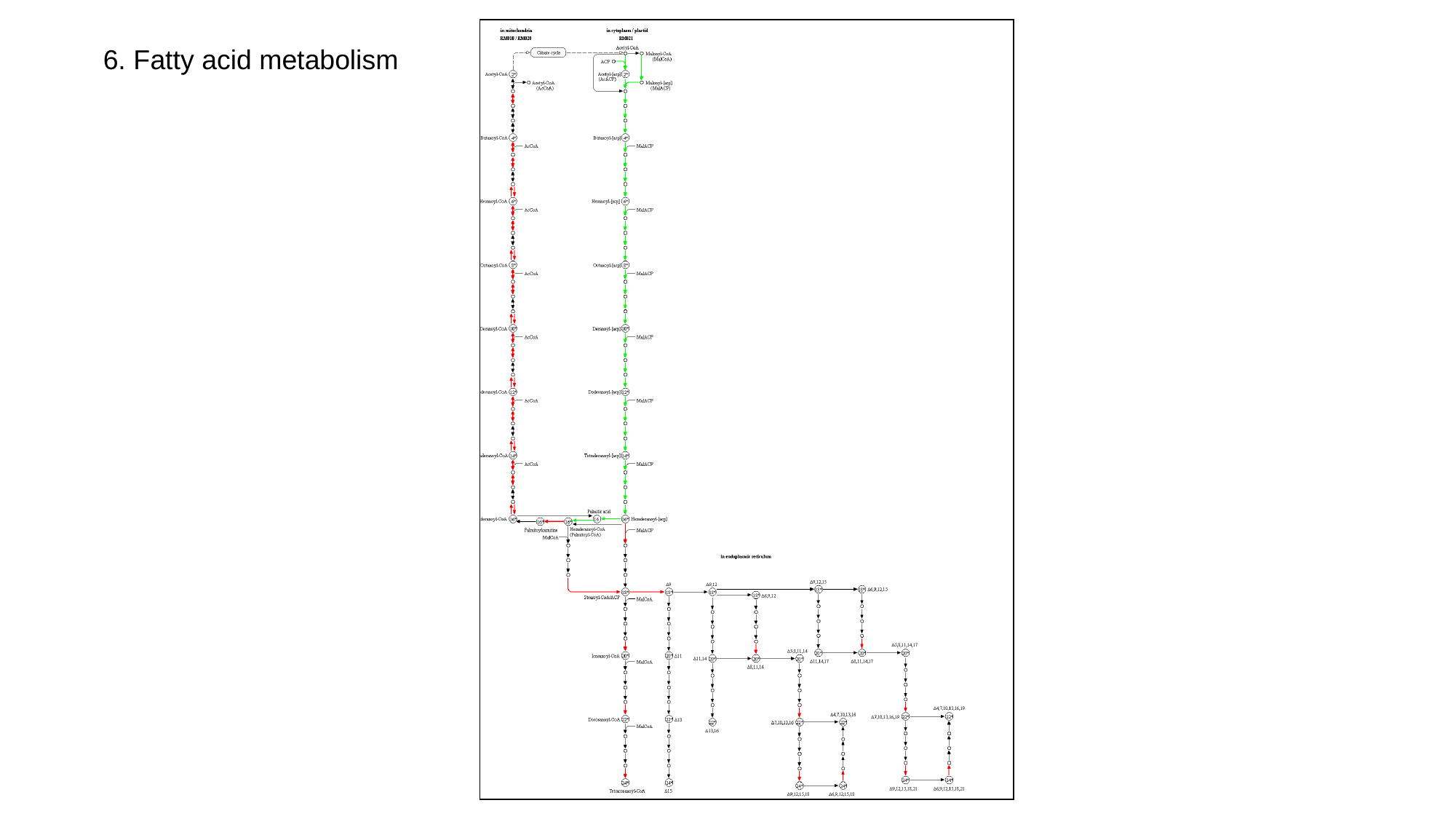

6. Fatty acid metabolism

## Slide 9
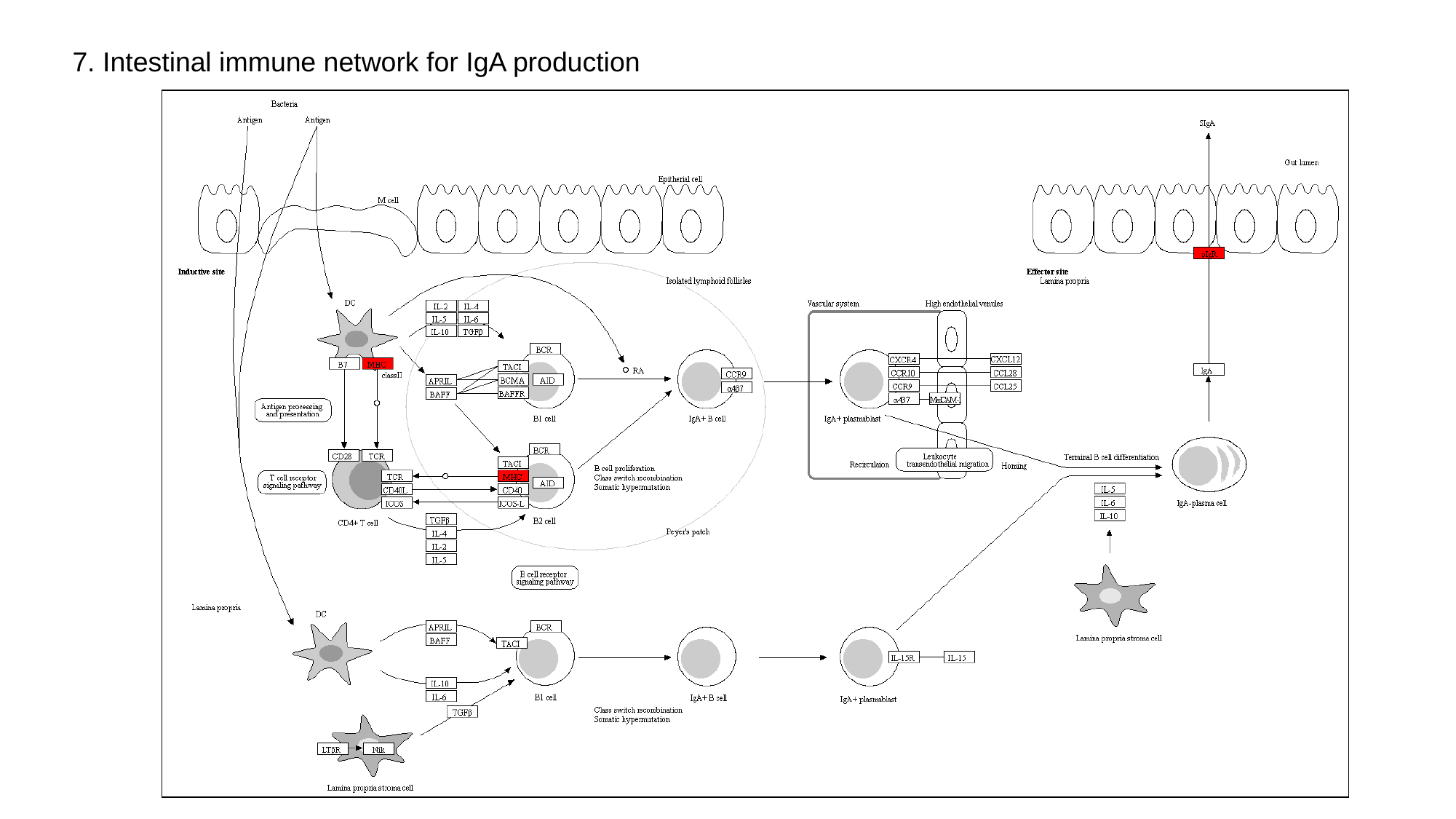

7. Intestinal immune network for IgA production

## Slide 10
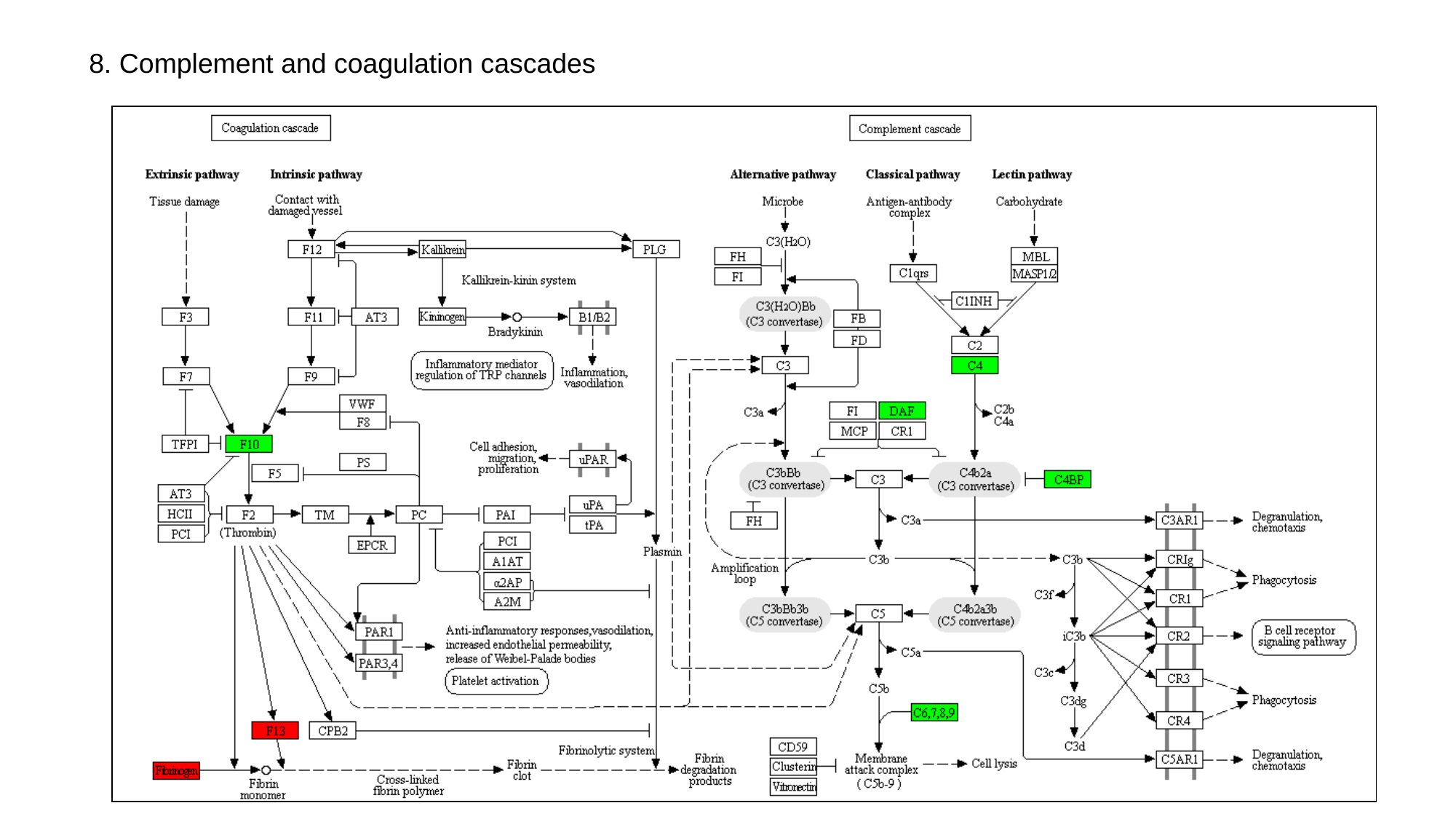

8. Complement and coagulation cascades

## Slide 11
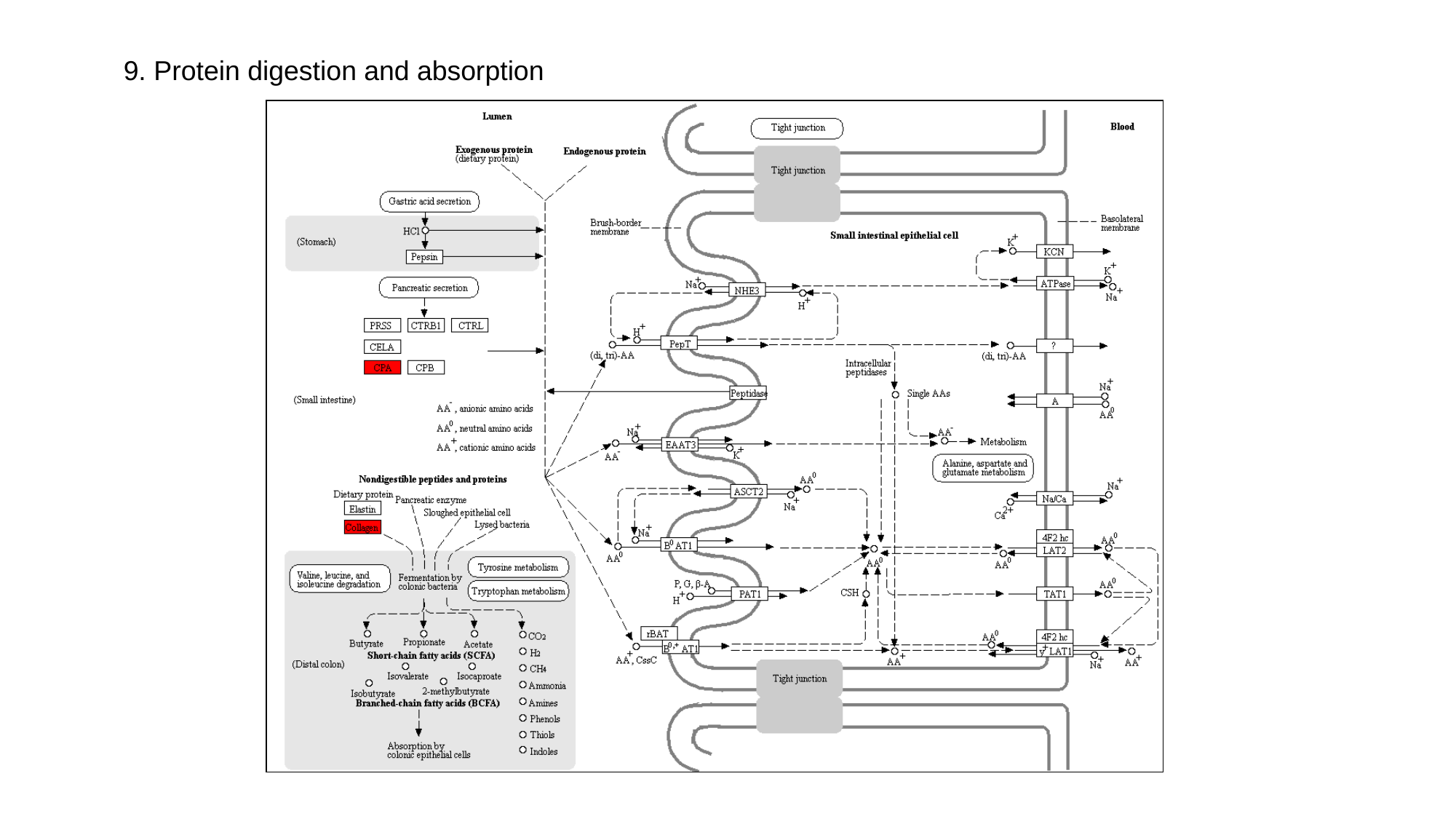

9. Protein digestion and absorption

## Slide 12
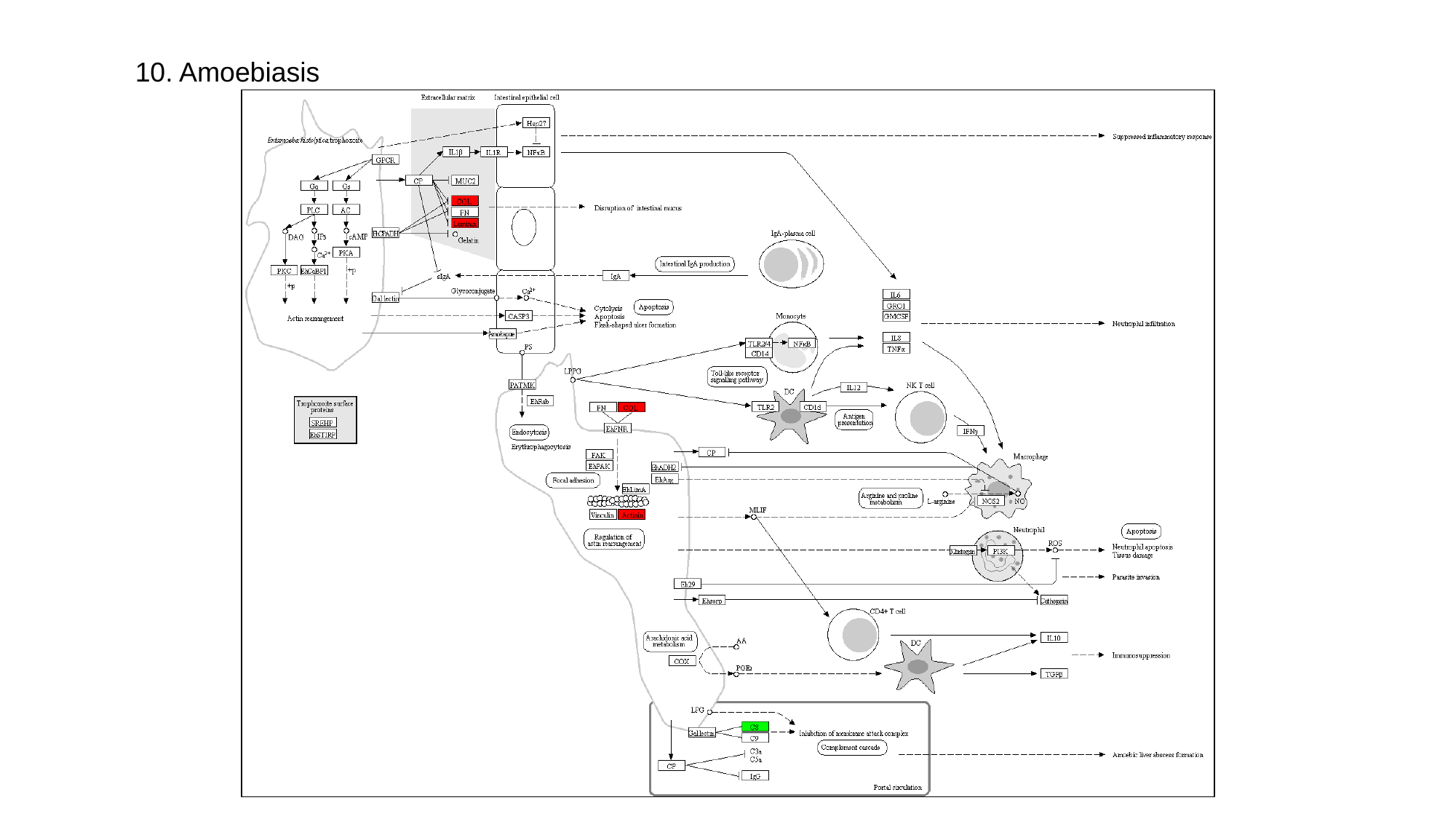

10. Amoebiasis

## Slide 13
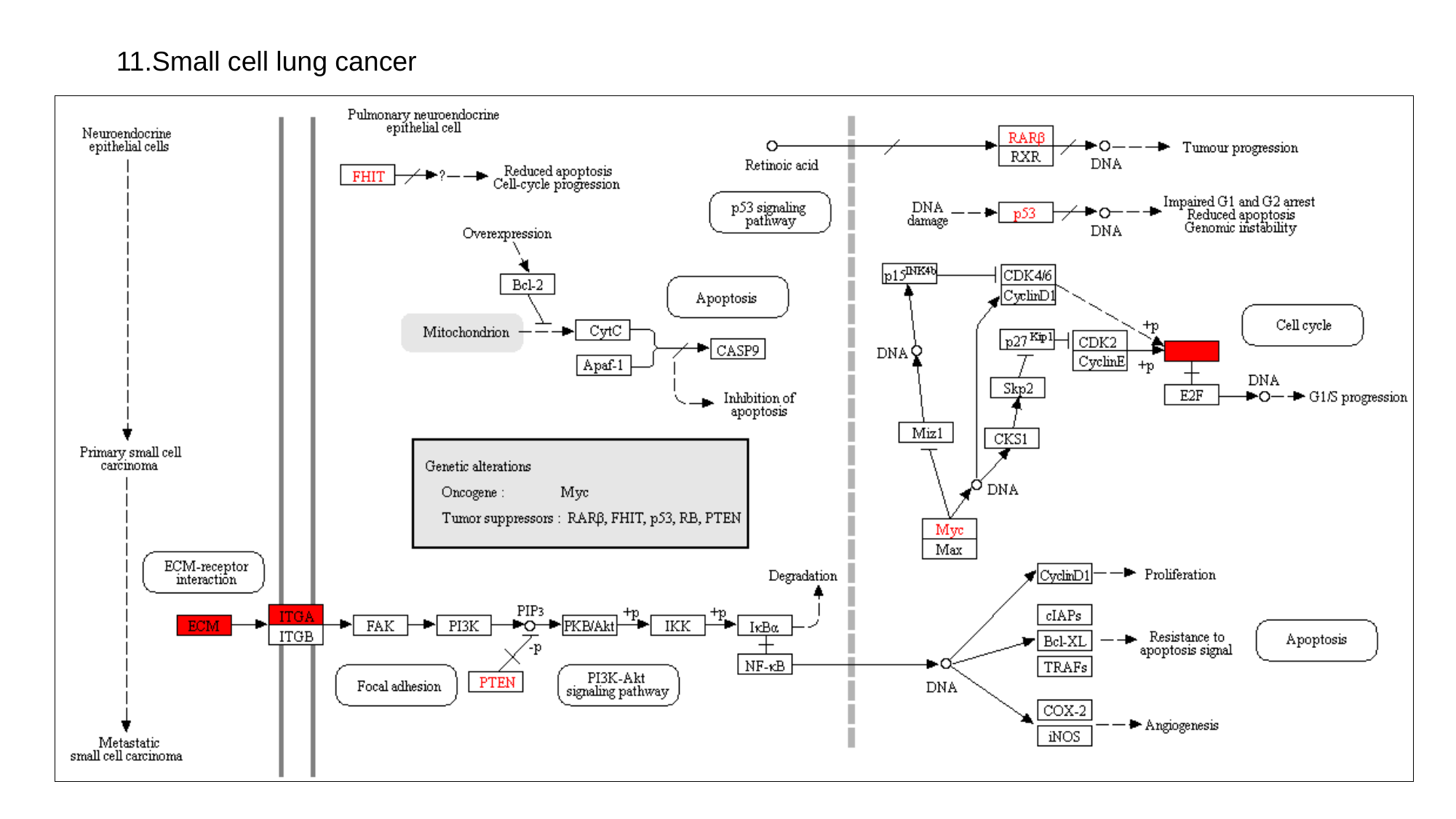

11.Small cell lung cancer

## Slide 14
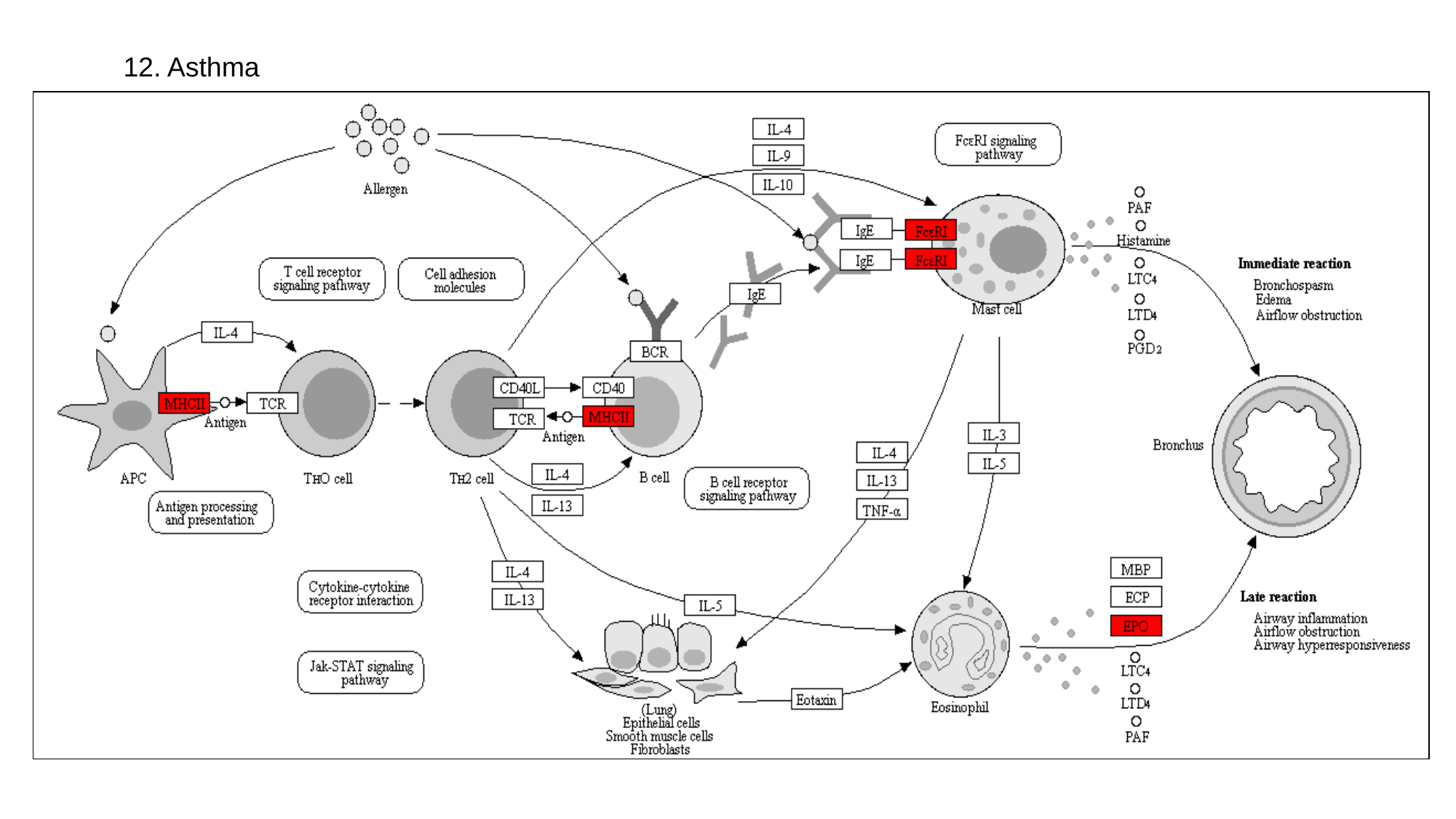

12. Asthma

## Slide 15
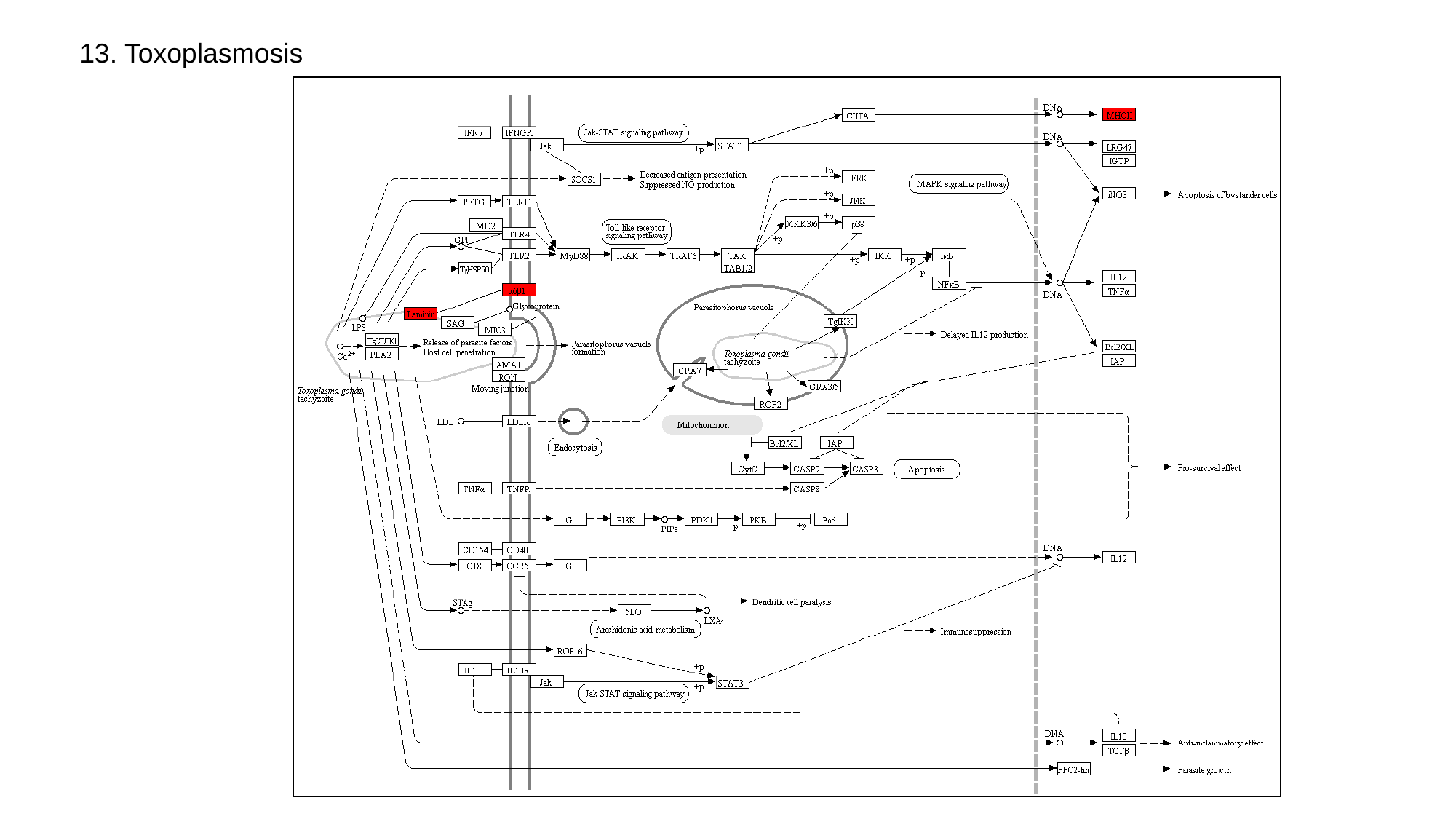

13. Toxoplasmosis

## Slide 16
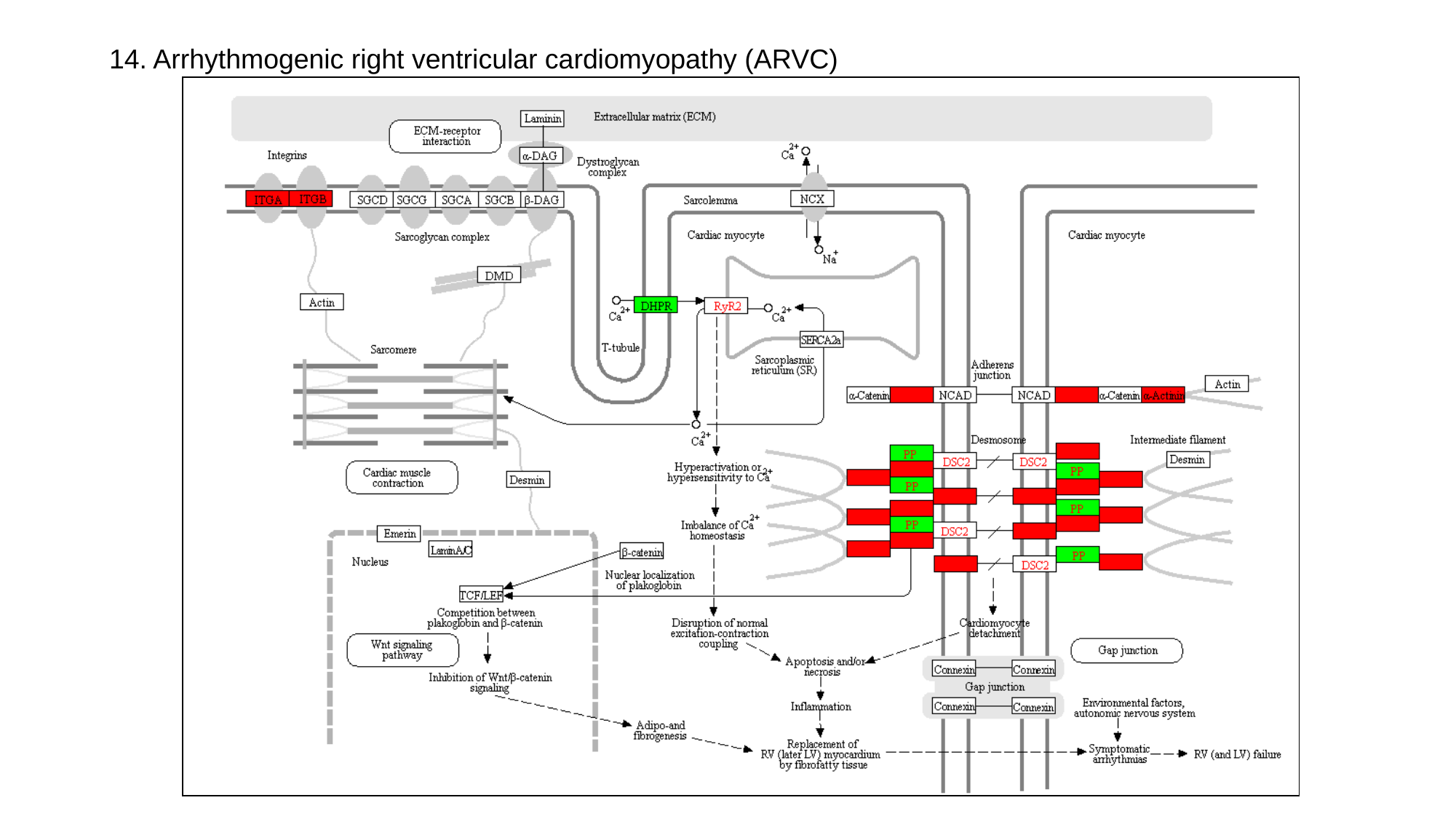

14. Arrhythmogenic right ventricular cardiomyopathy (ARVC)

## Slide 17
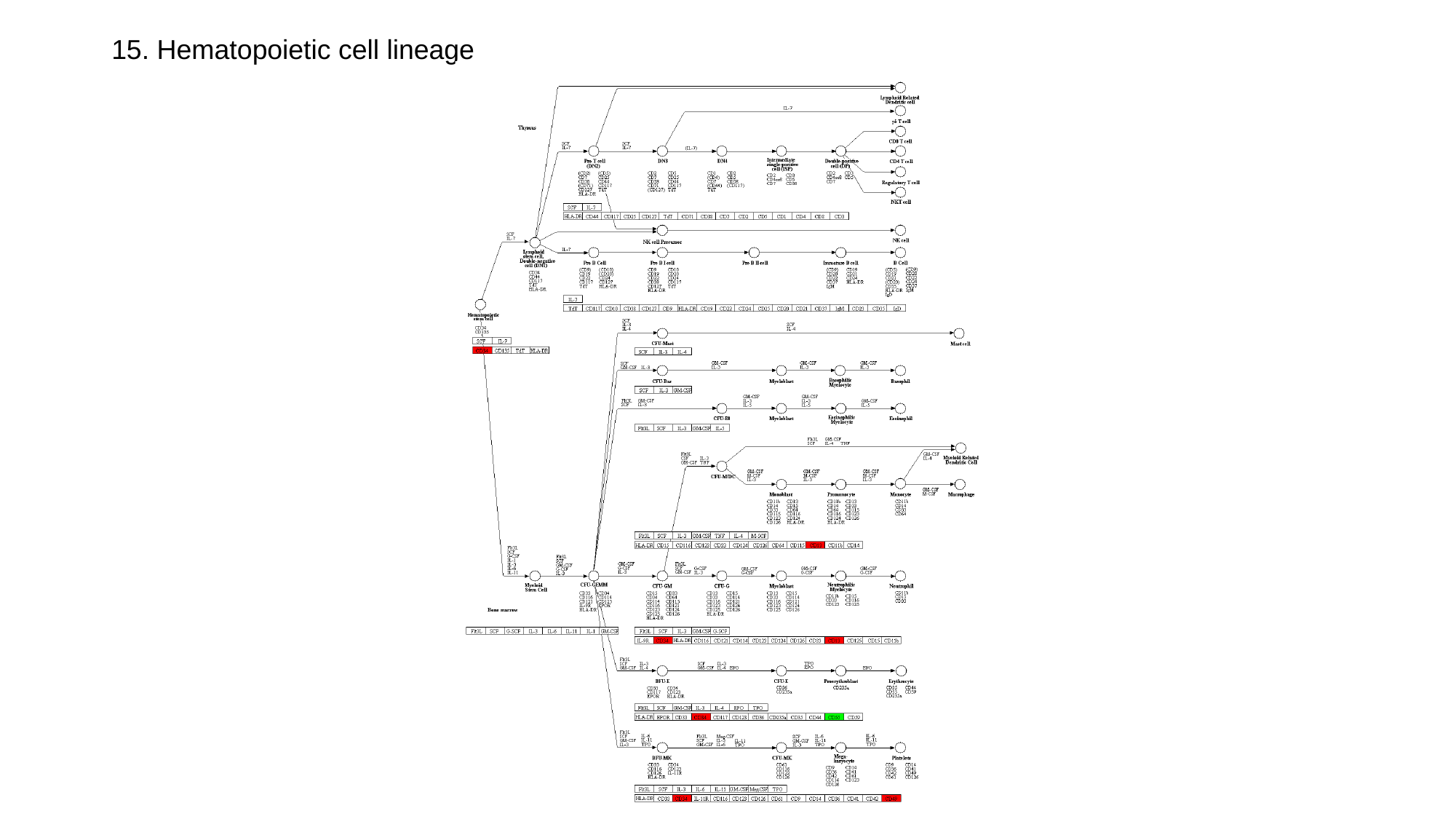

15. Hematopoietic cell lineage

## Slide 18
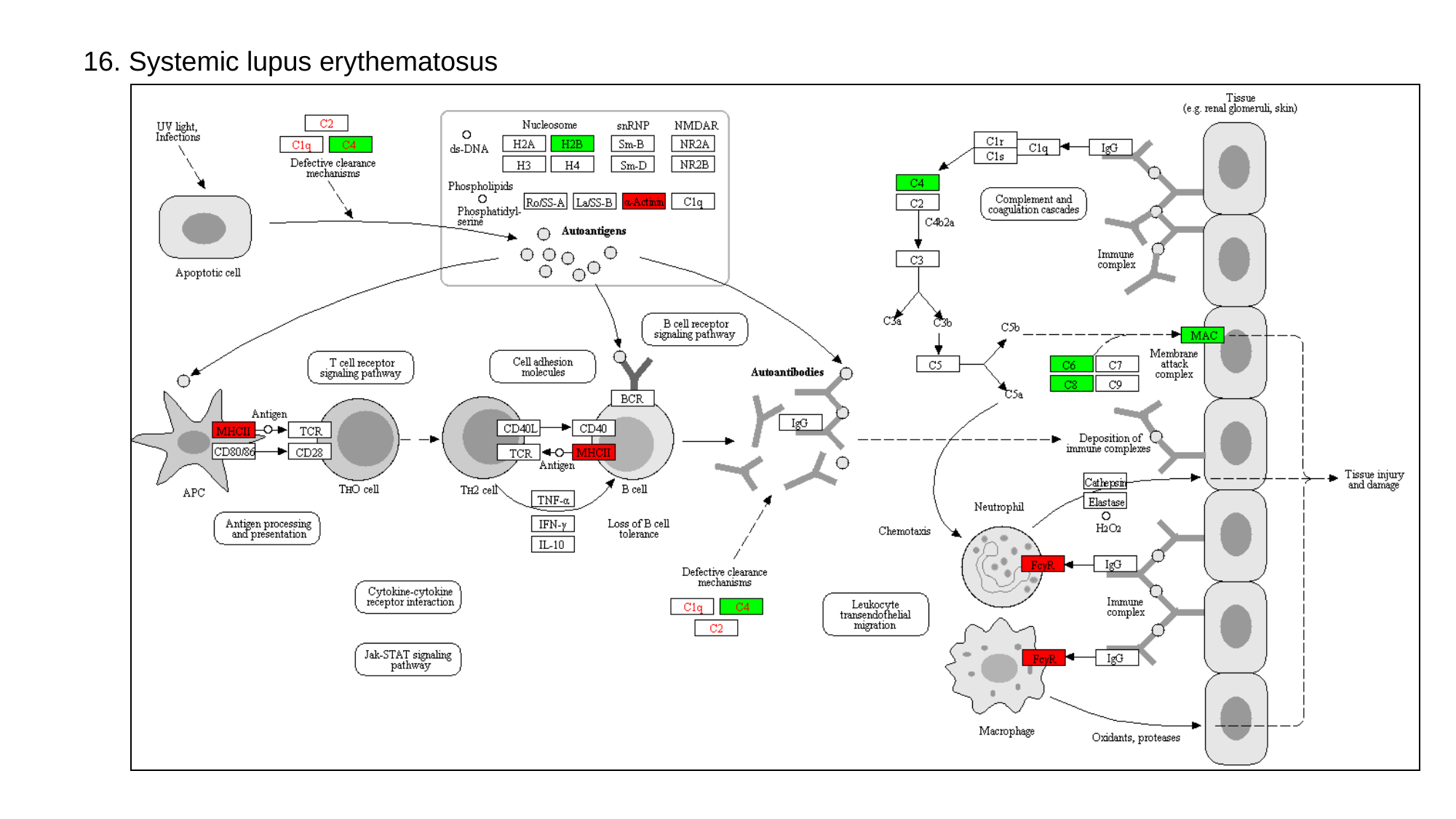

16. Systemic lupus erythematosus

## Slide 19
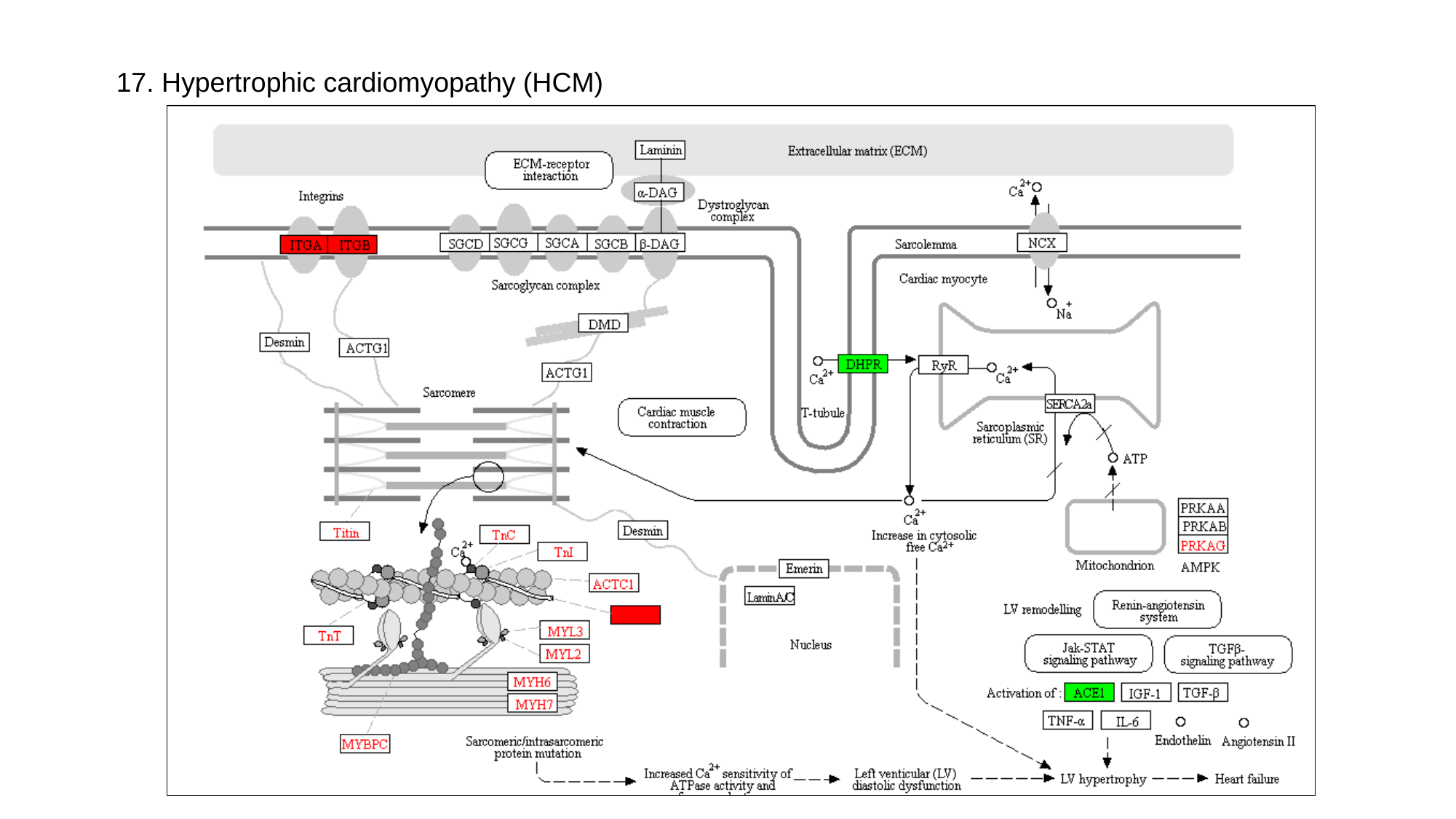

17. Hypertrophic cardiomyopathy (HCM)

## Slide 20
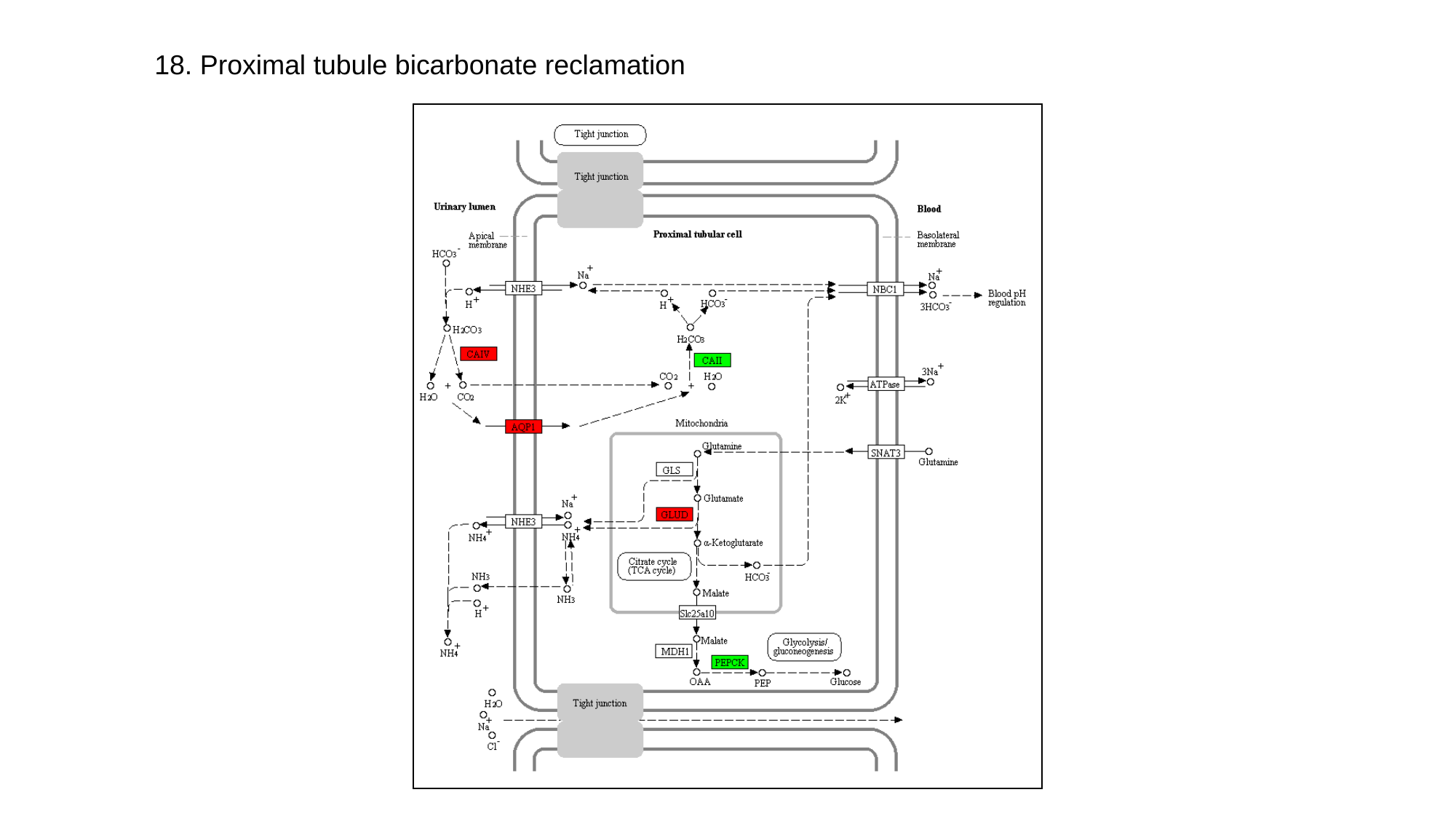

18. Proximal tubule bicarbonate reclamation

## Slide 21
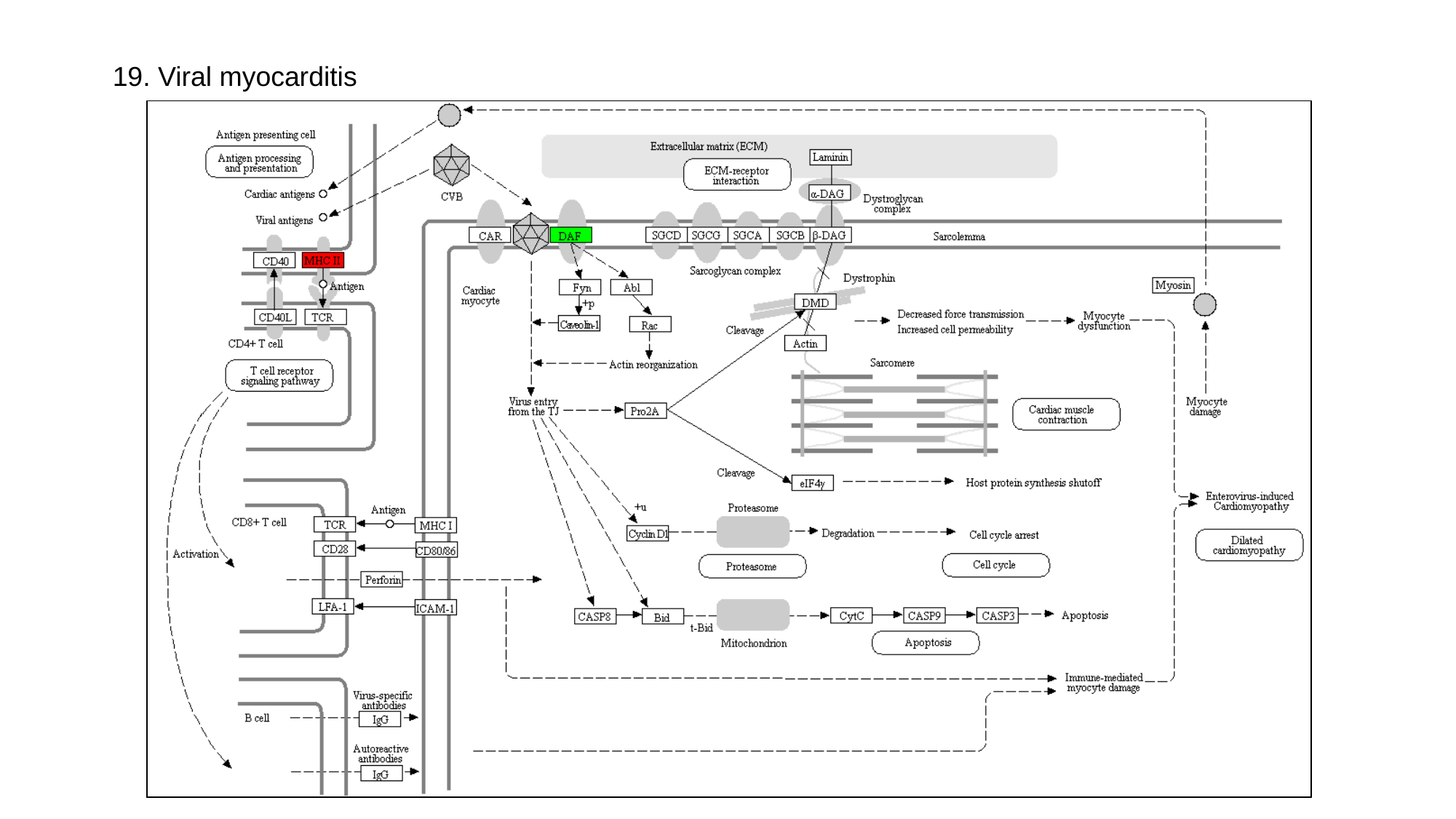

19. Viral myocarditis
