# Supplementary material for: TMT-based quantitative proteomics revealed follicle-stimulating hormone (FSH)-related molecular characterizations for potentially prognostic assessment and personalized treatment of FSH-positive non-functional pituitary adenomas
Source: EPMA J. 2019 Aug 29;10(4):395–414. doi: 10.1007/s13167-019-00187-w (PMC6882982; doi:10.1007/s13167-019-00187-w)
Supplement: Supplementary file 2 — (PPT 1366 kb) [file 13167_2019_187_MOESM2_ESM.ppt]

## Slide 1
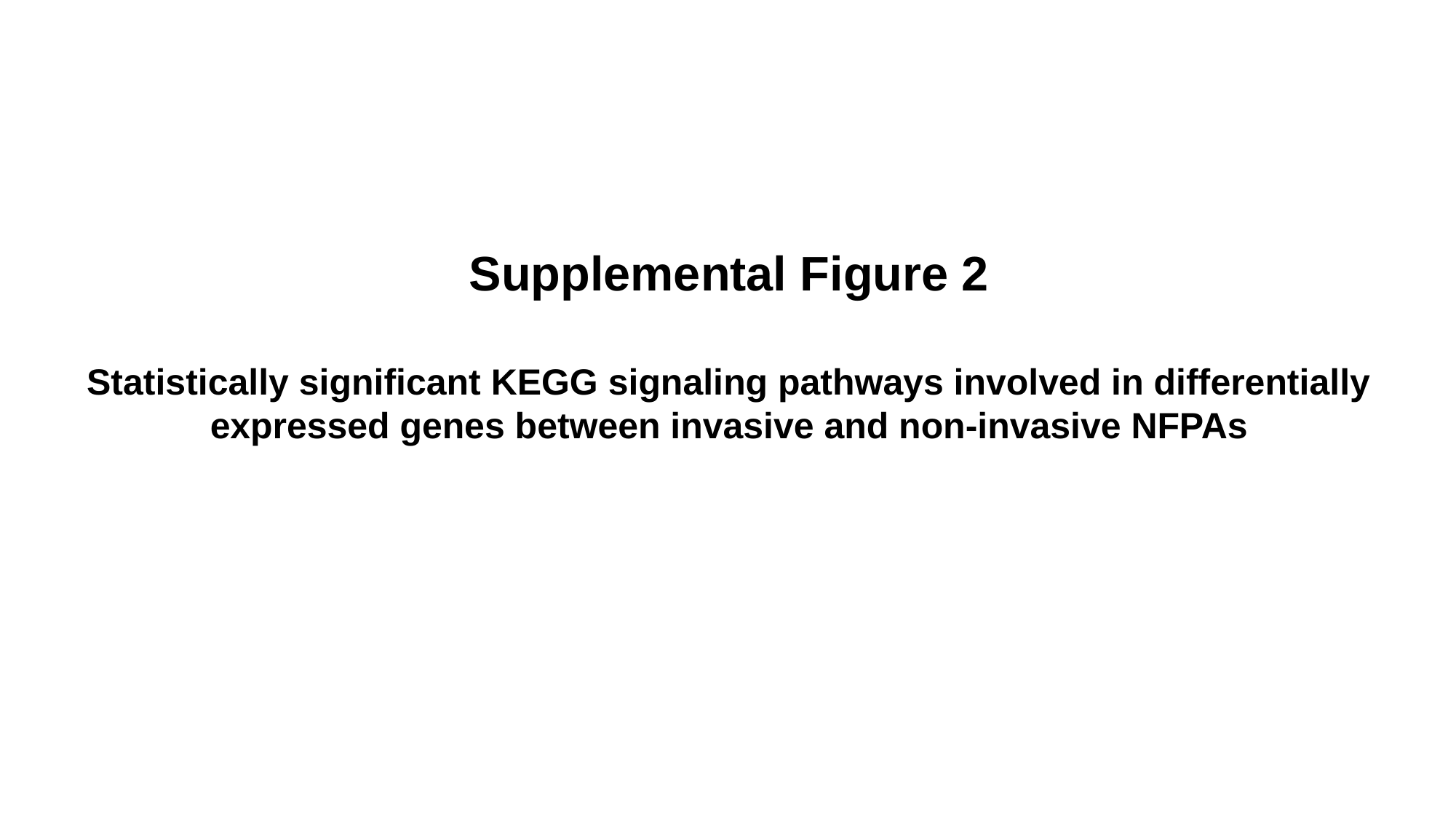

Supplemental Figure 2
Statistically significant KEGG signaling pathways involved in differentially expressed genes between invasive and non-invasive NFPAs

## Slide 2
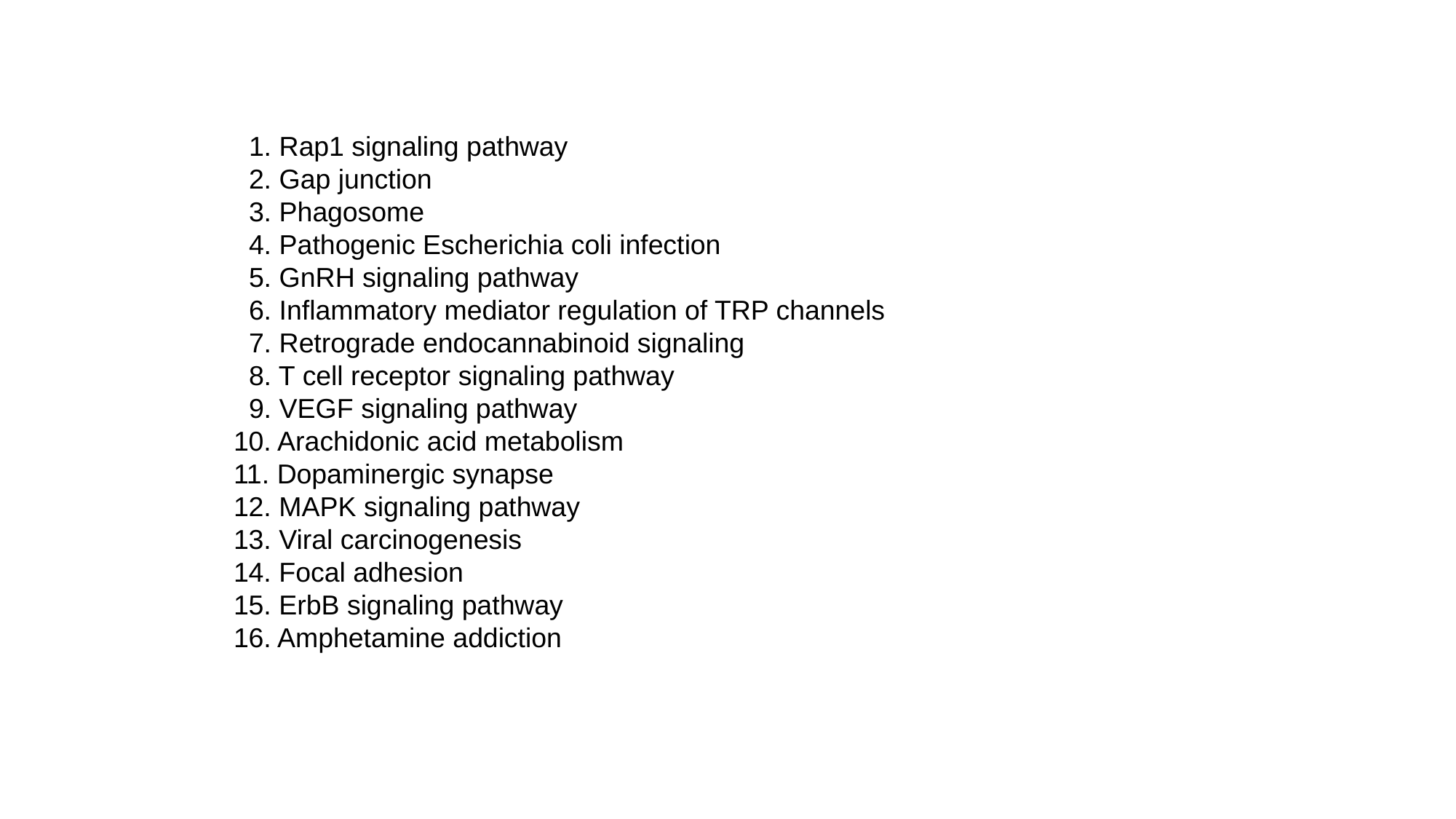

1. Rap1 signaling pathway
 2. Gap junction
 3. Phagosome
 4. Pathogenic Escherichia coli infection
 5. GnRH signaling pathway
 6. Inflammatory mediator regulation of TRP channels
 7. Retrograde endocannabinoid signaling
 8. T cell receptor signaling pathway
 9. VEGF signaling pathway
10. Arachidonic acid metabolism
11. Dopaminergic synapse
12. MAPK signaling pathway
13. Viral carcinogenesis
14. Focal adhesion
15. ErbB signaling pathway
16. Amphetamine addiction

## Slide 3
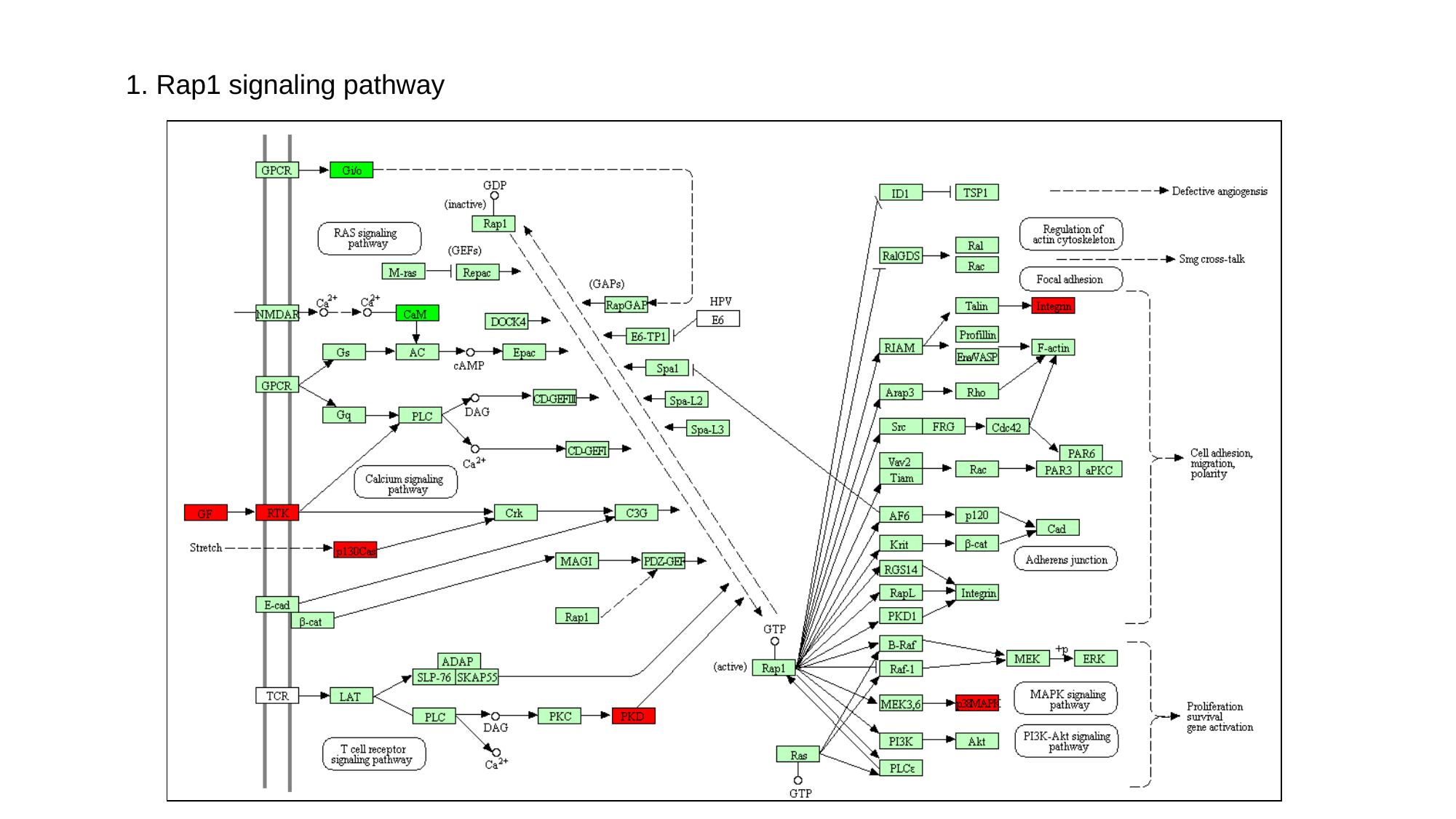

1. Rap1 signaling pathway

## Slide 4
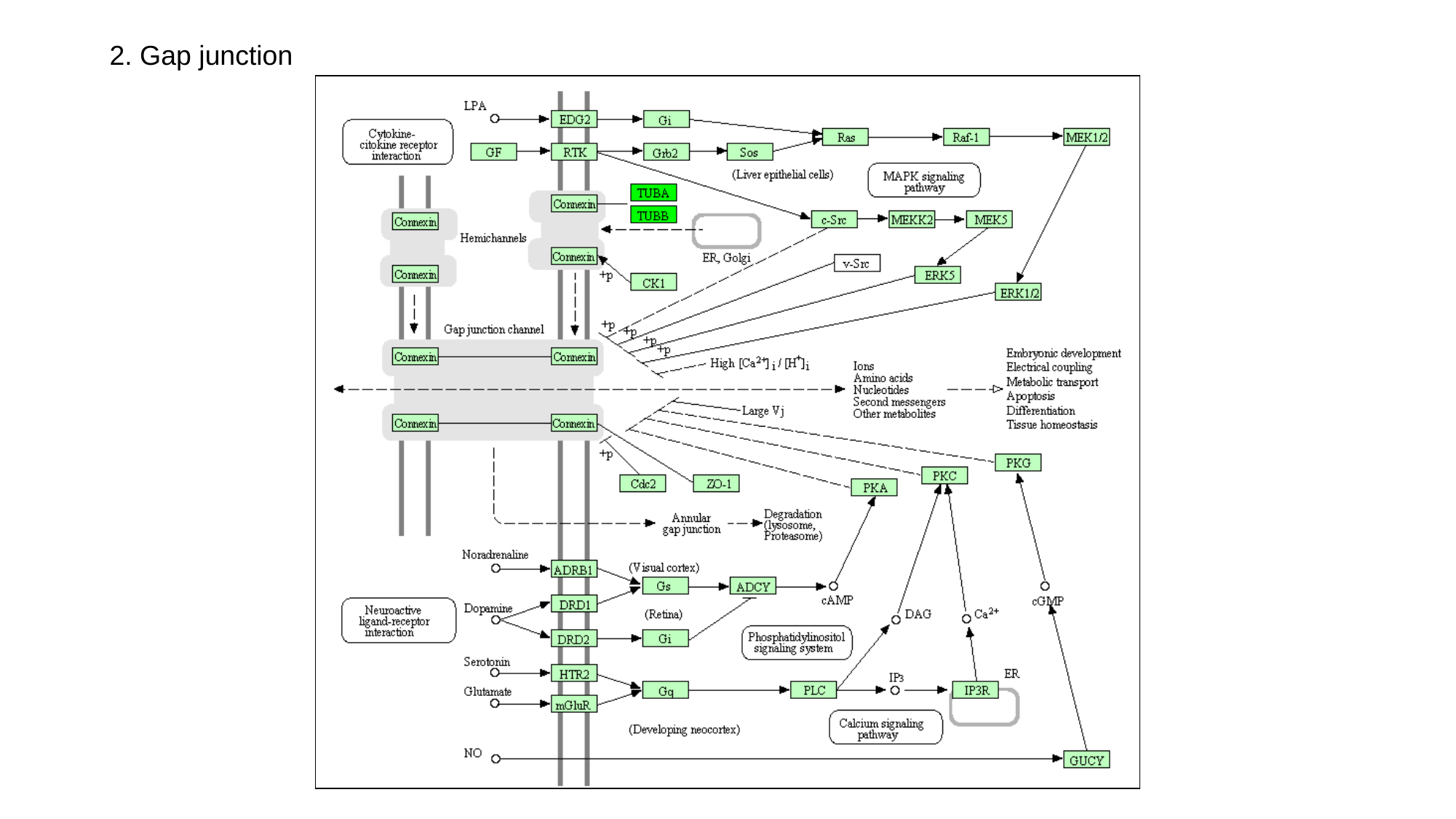

2. Gap junction

## Slide 5
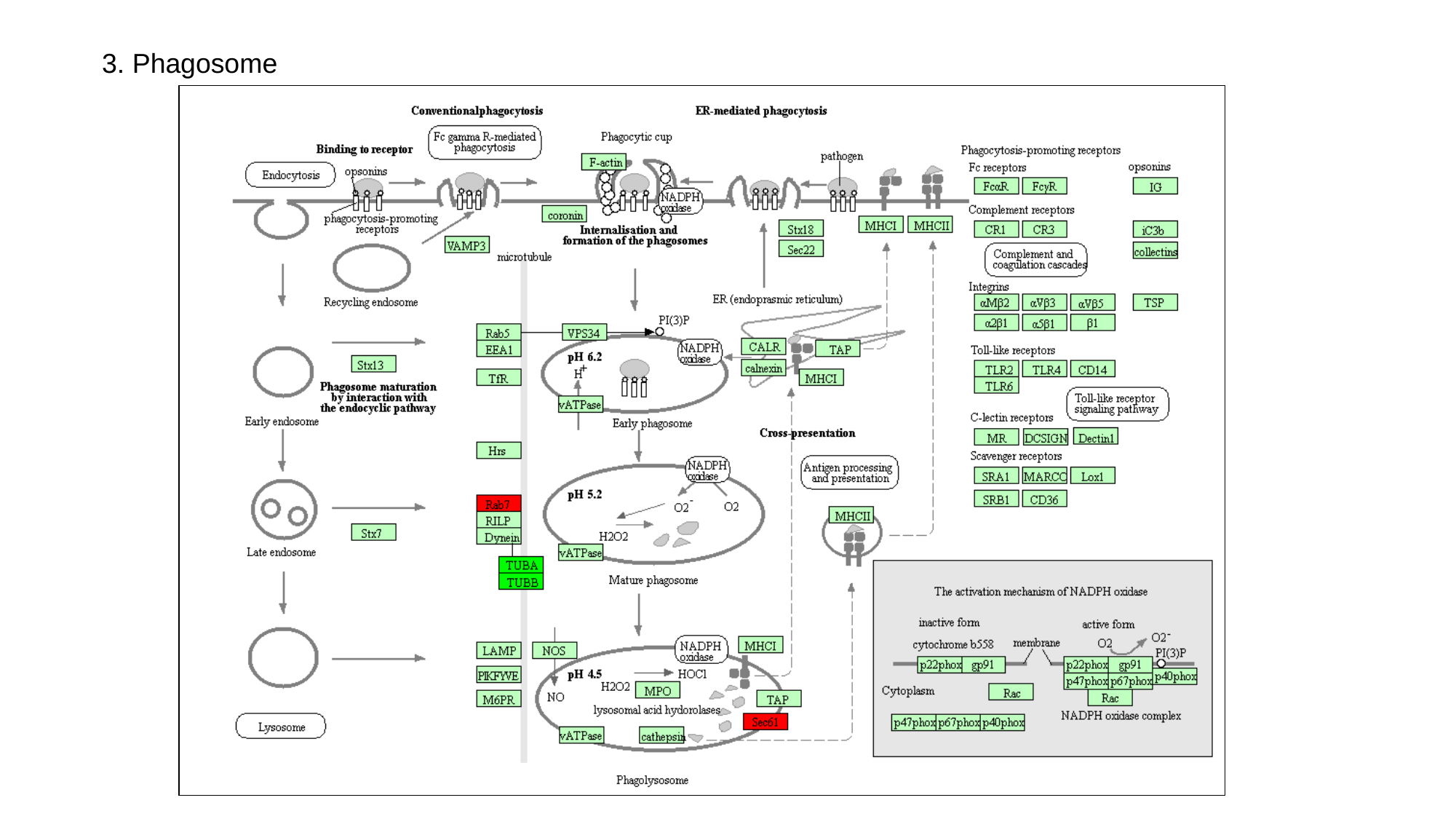

3. Phagosome

## Slide 6
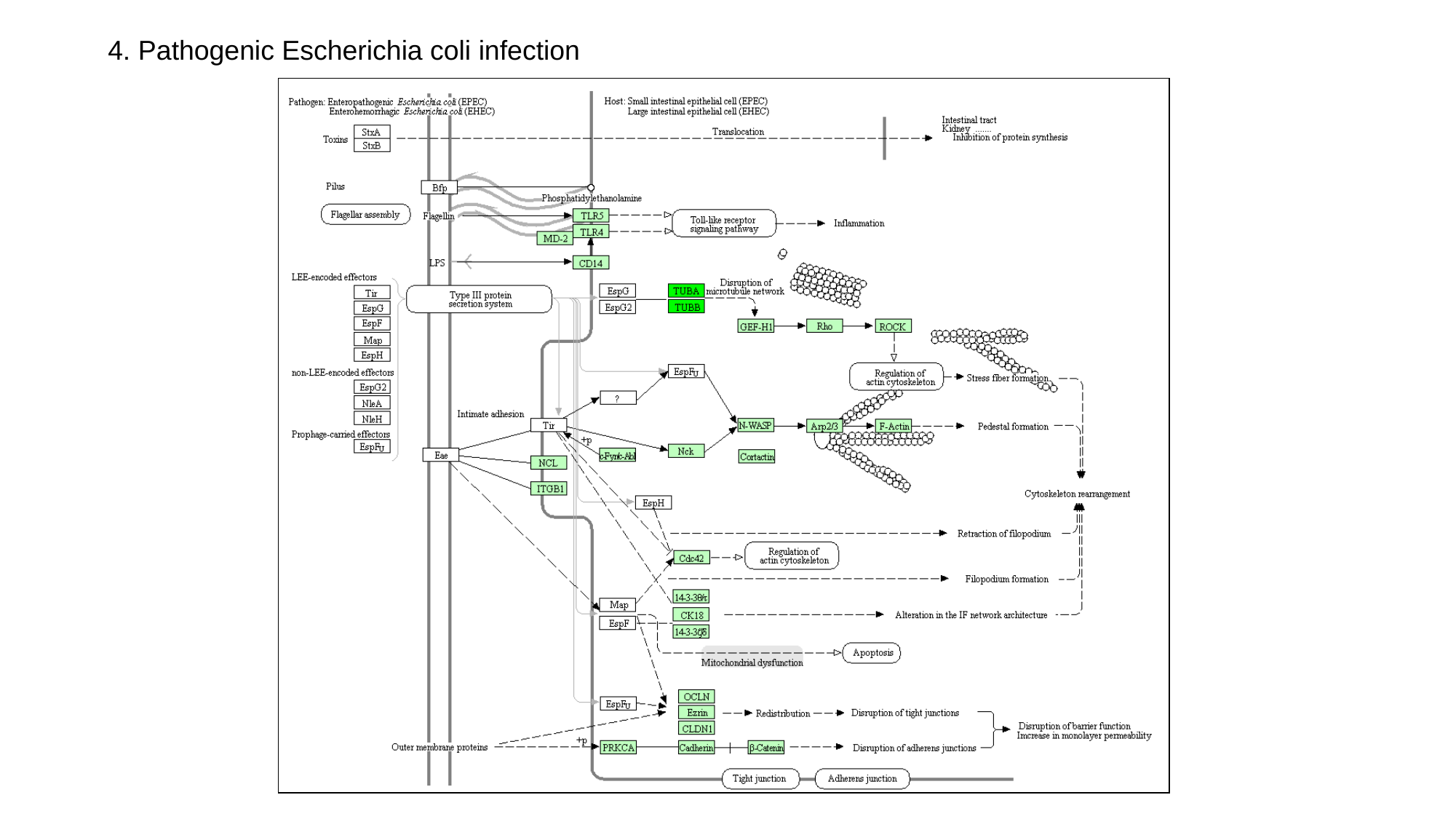

4. Pathogenic Escherichia coli infection

## Slide 7
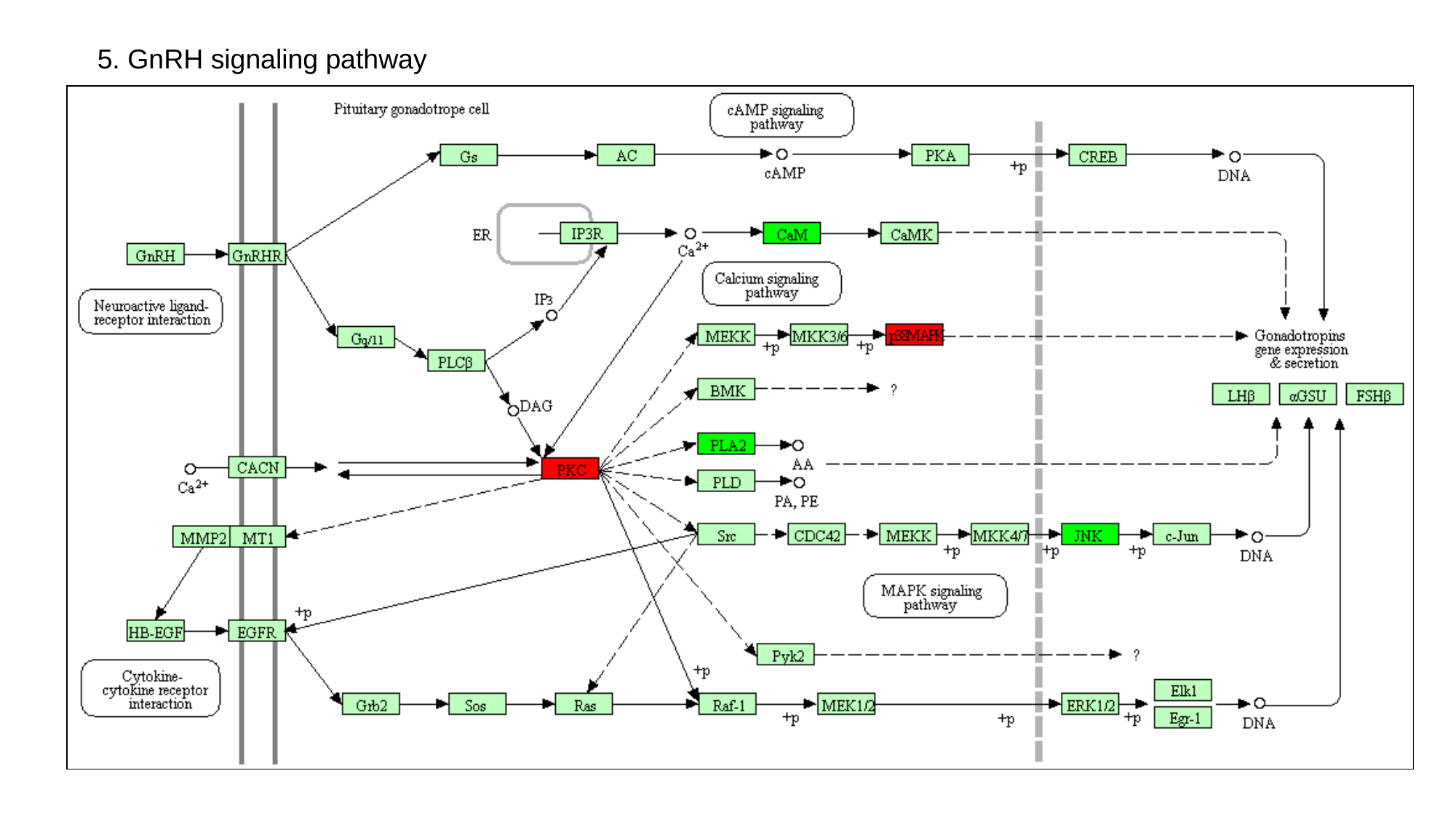

5. GnRH signaling pathway

## Slide 8
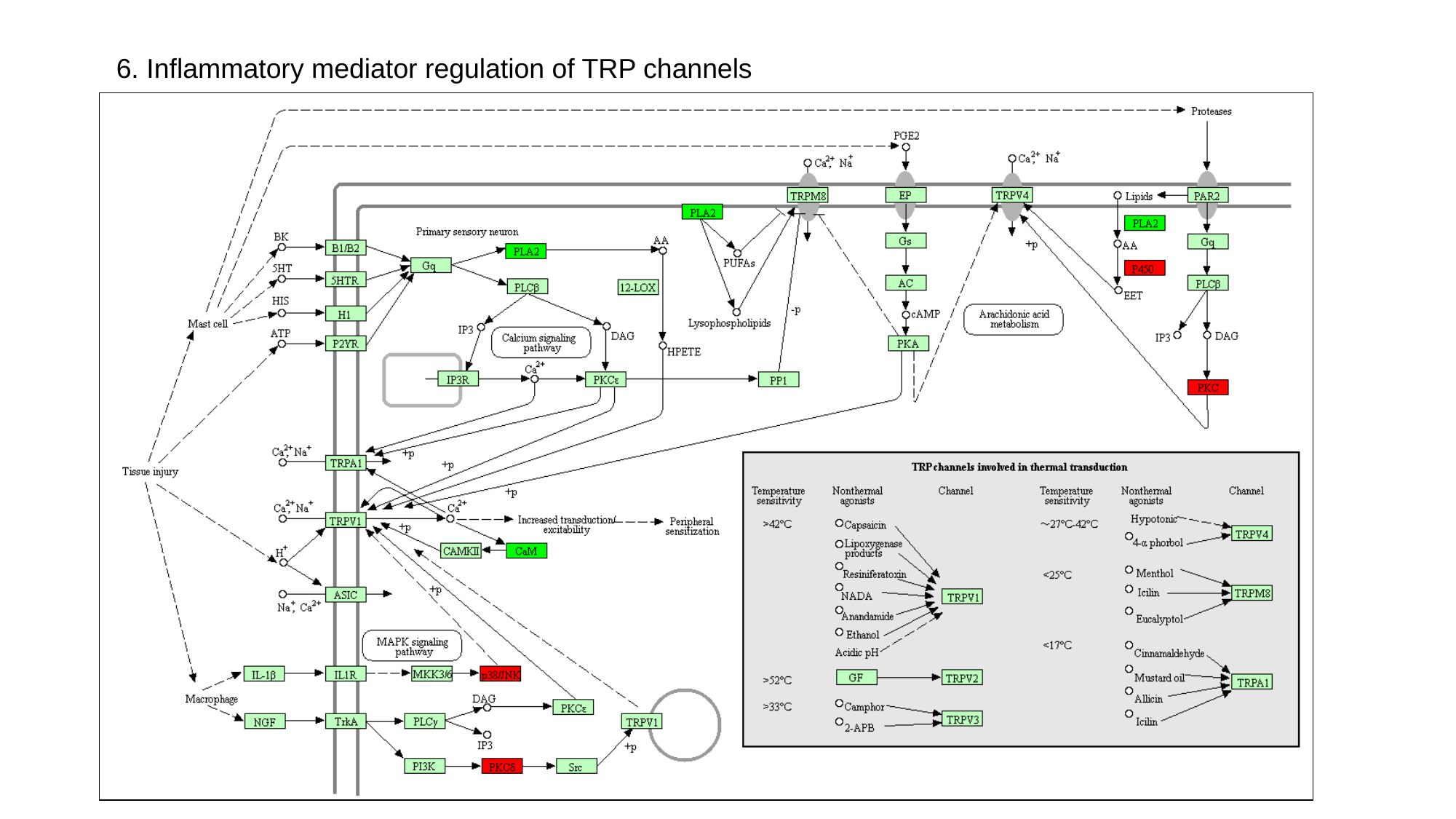

6. Inflammatory mediator regulation of TRP channels

## Slide 9
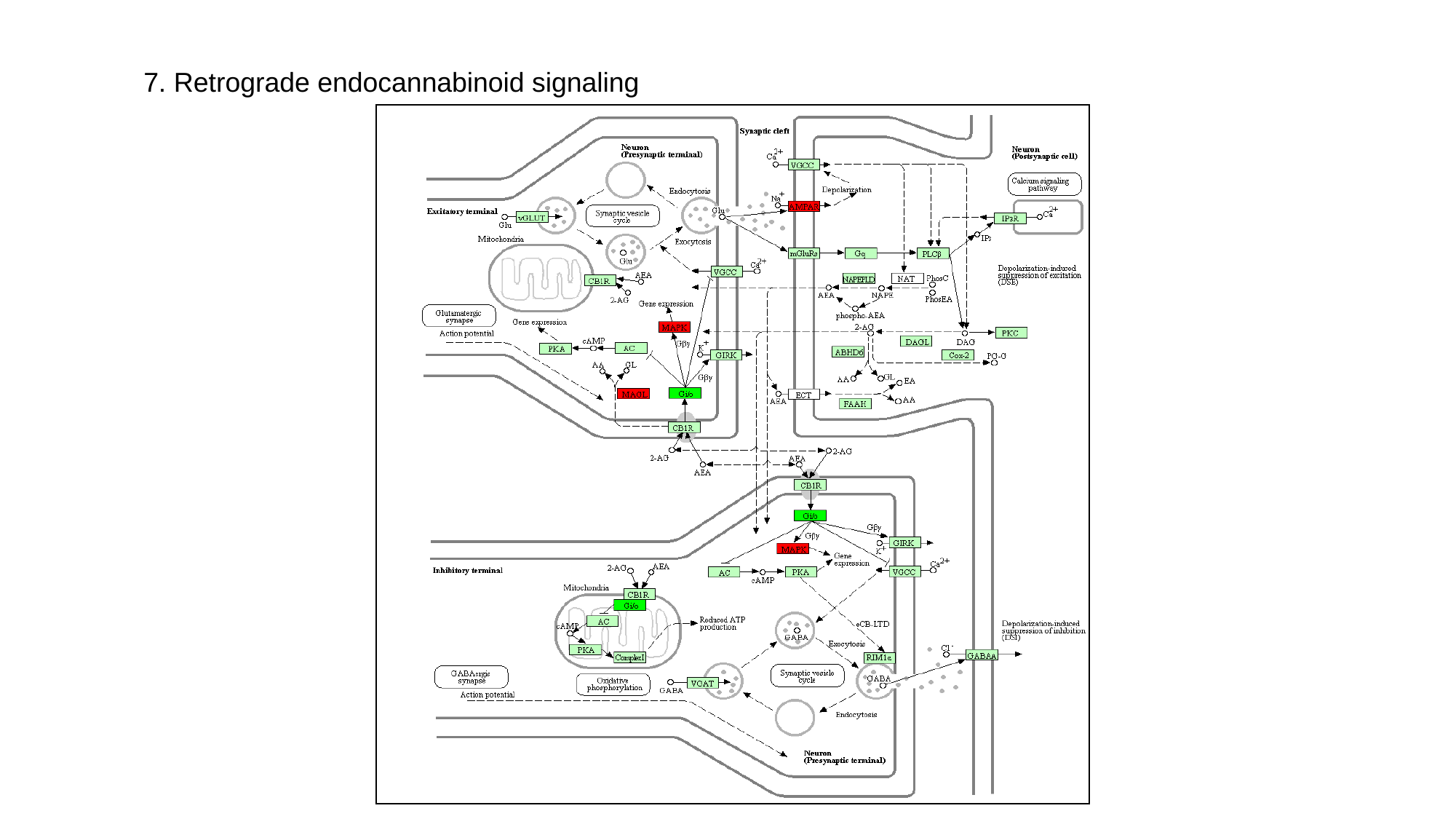

7. Retrograde endocannabinoid signaling

## Slide 10
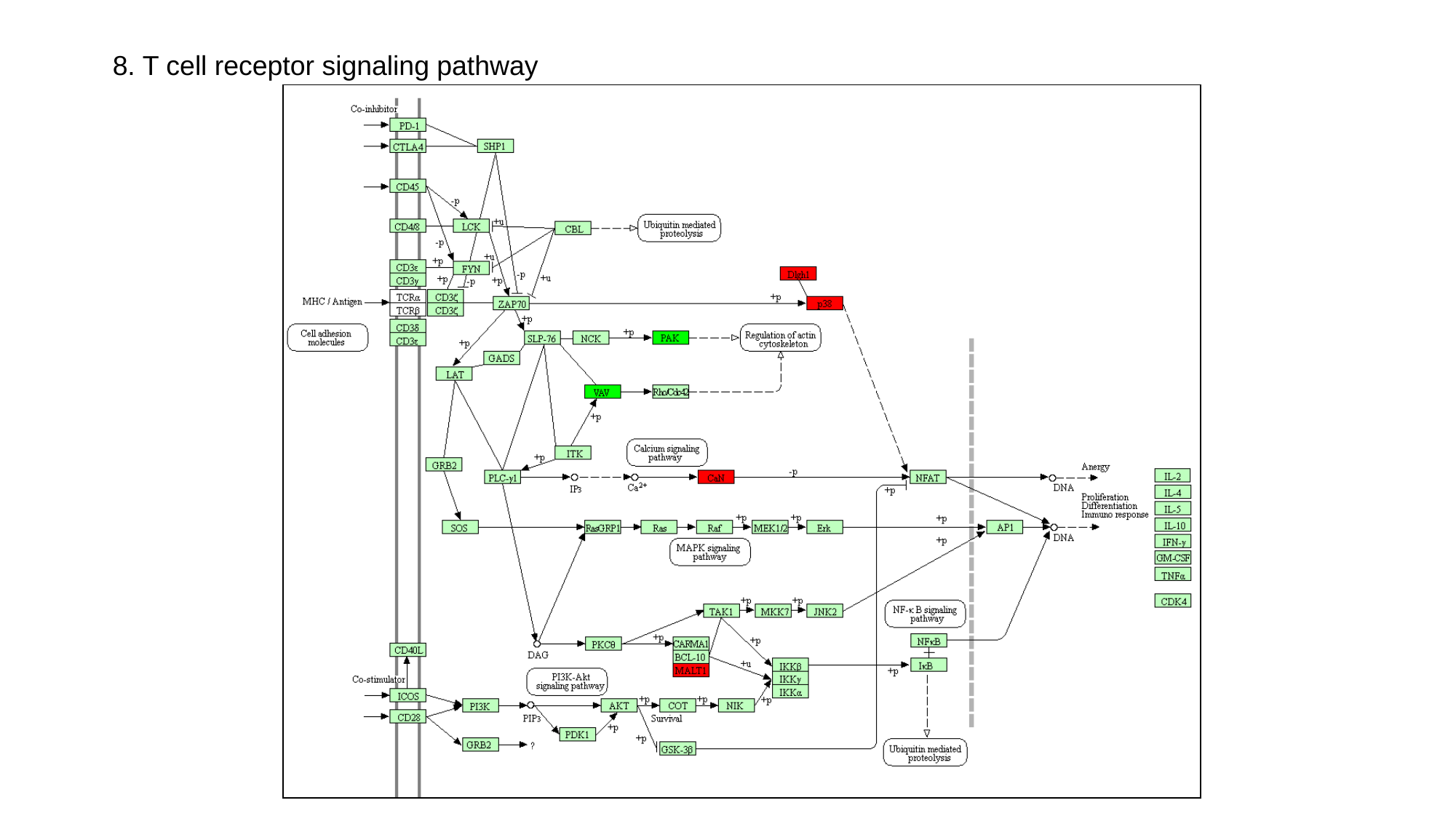

8. T cell receptor signaling pathway

## Slide 11
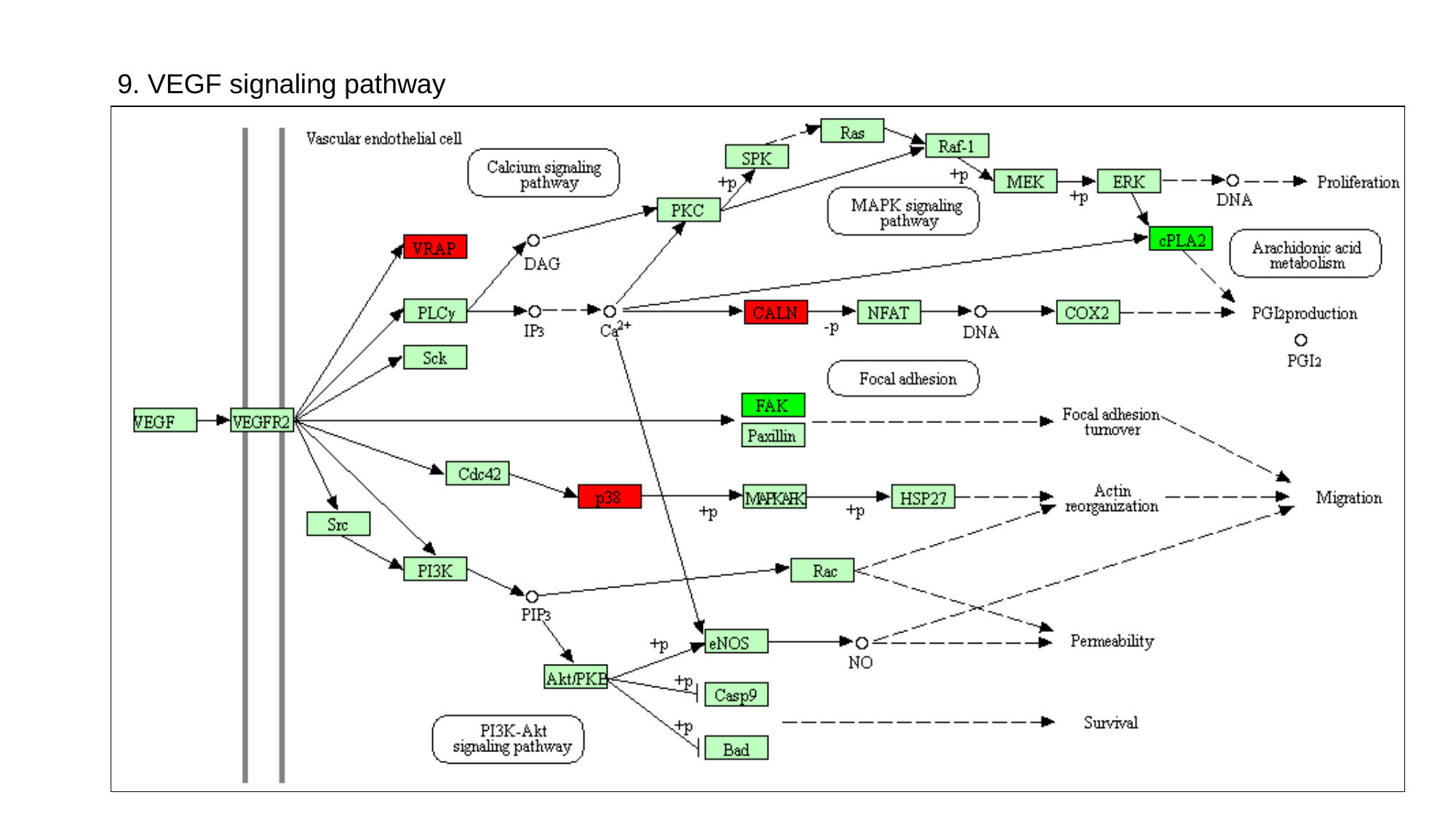

9. VEGF signaling pathway

## Slide 12
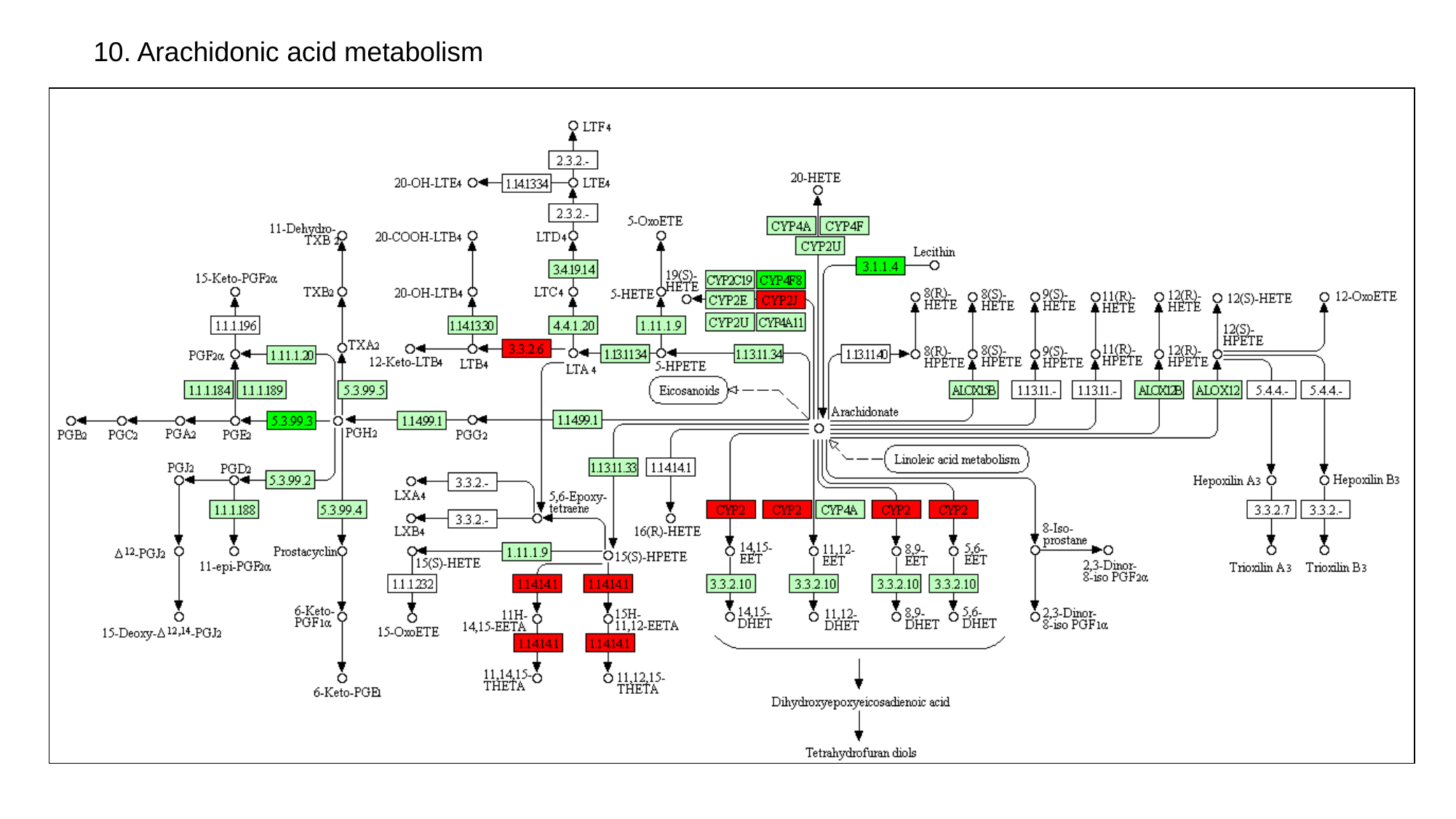

10. Arachidonic acid metabolism

## Slide 13
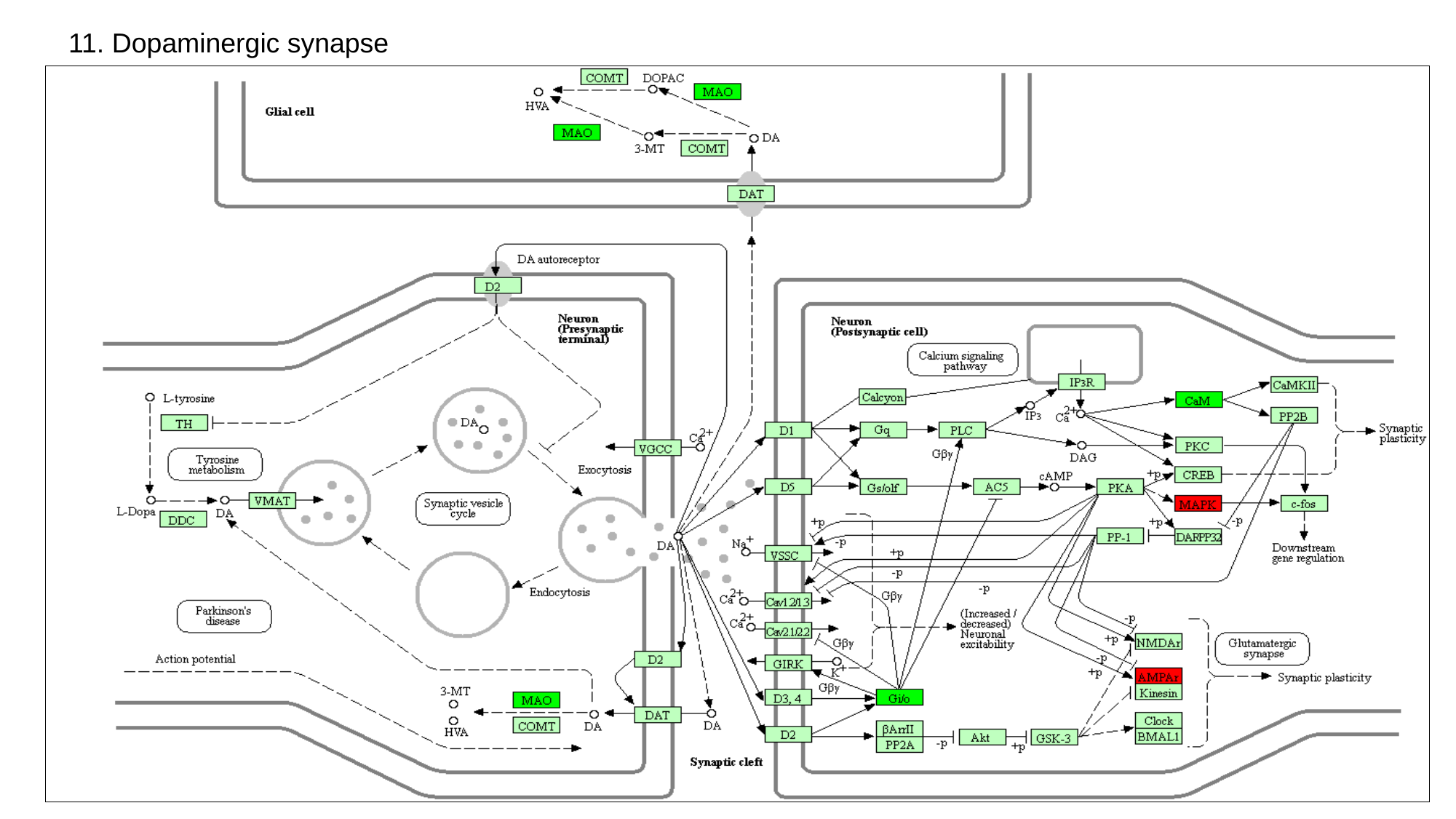

11. Dopaminergic synapse

## Slide 14
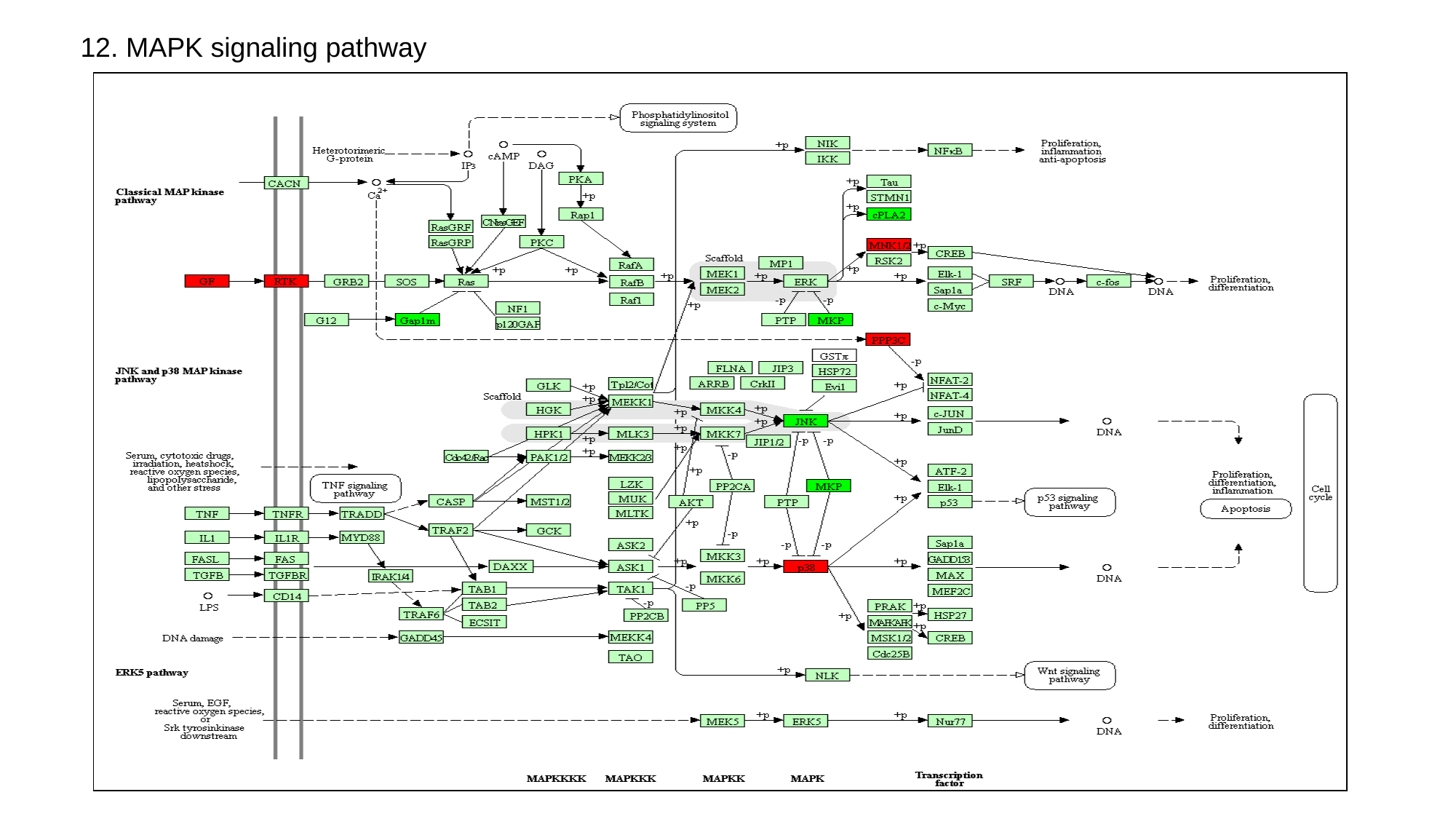

12. MAPK signaling pathway

## Slide 15
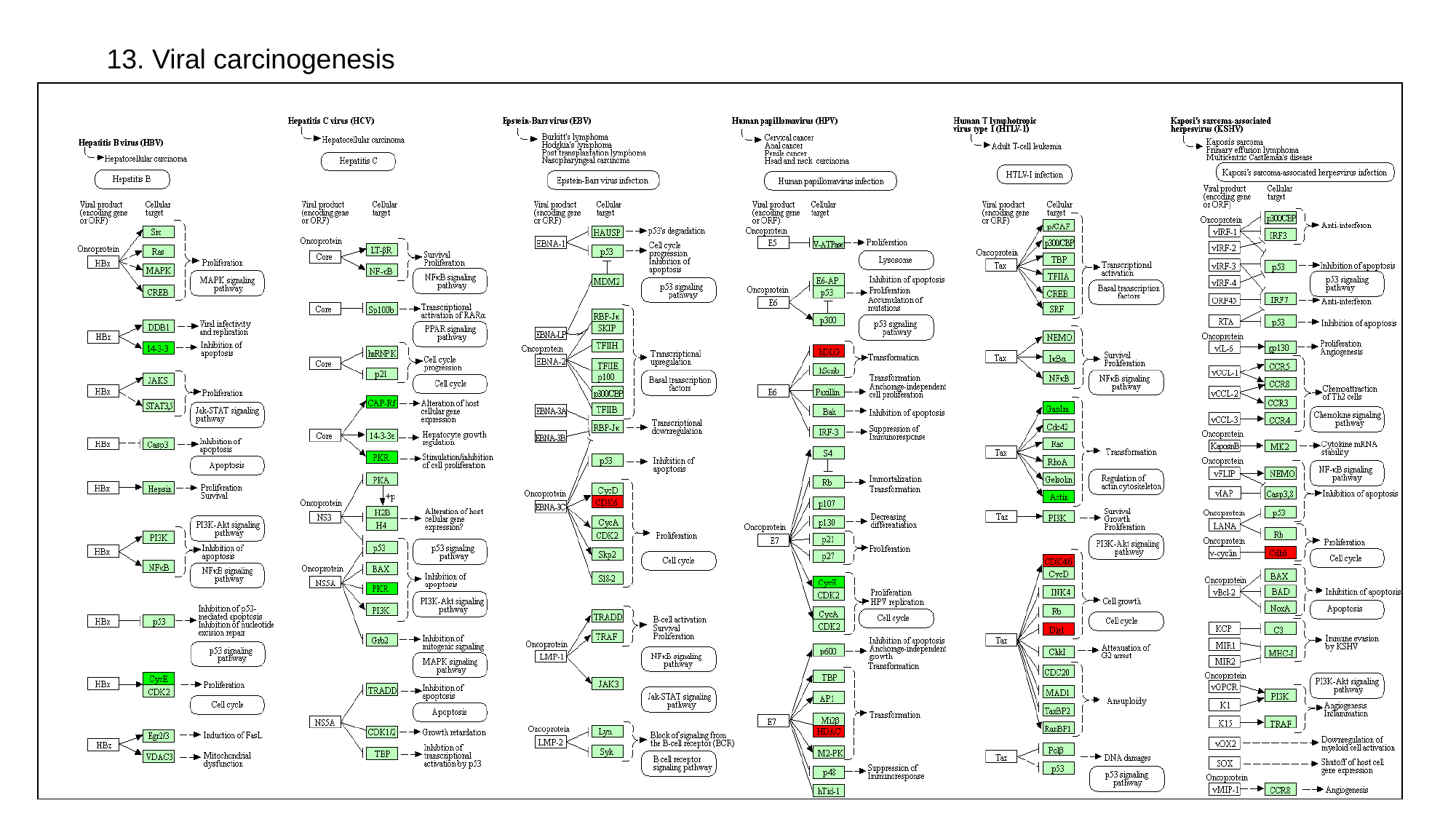

13. Viral carcinogenesis

## Slide 16
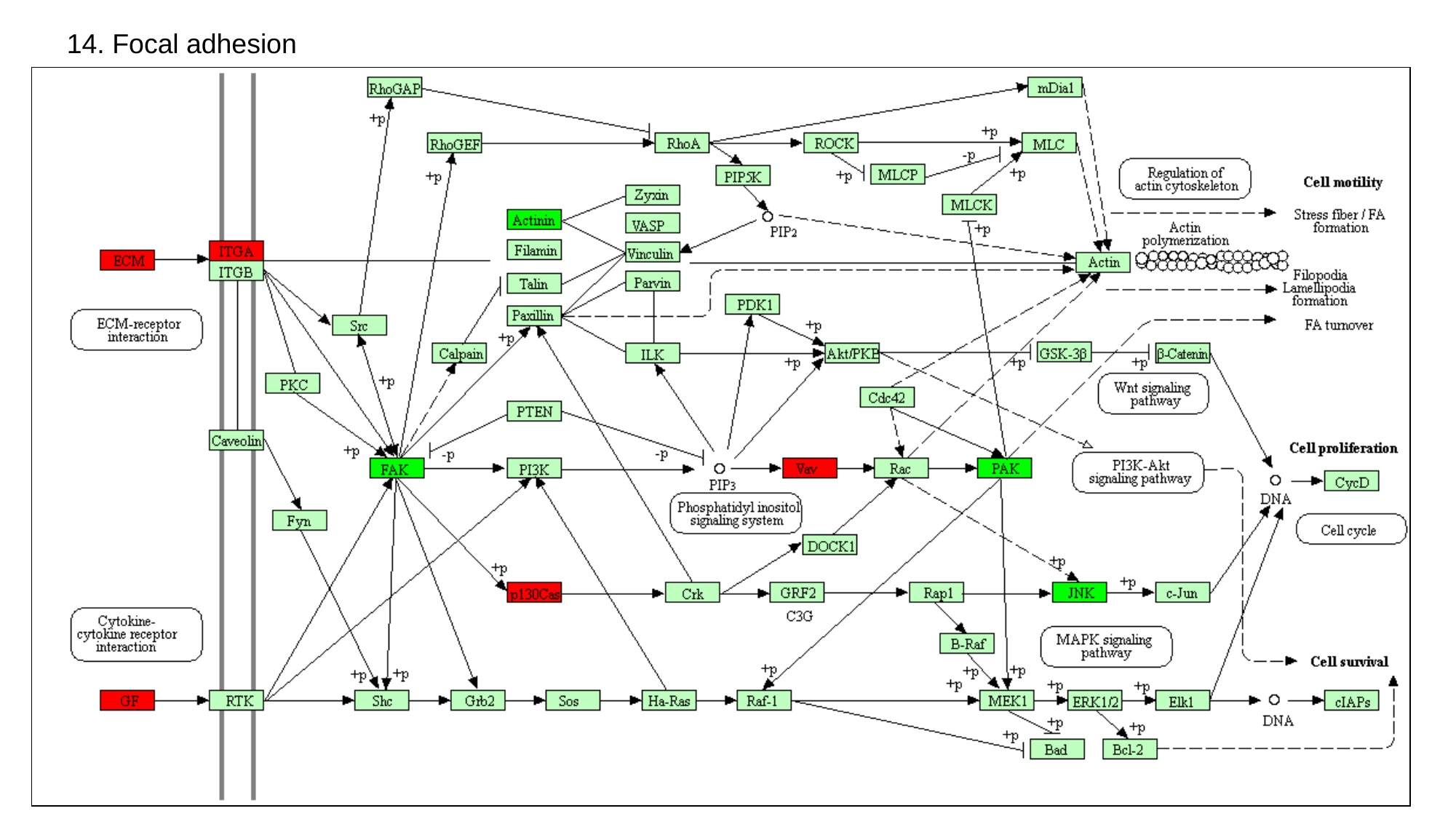

14. Focal adhesion

## Slide 17
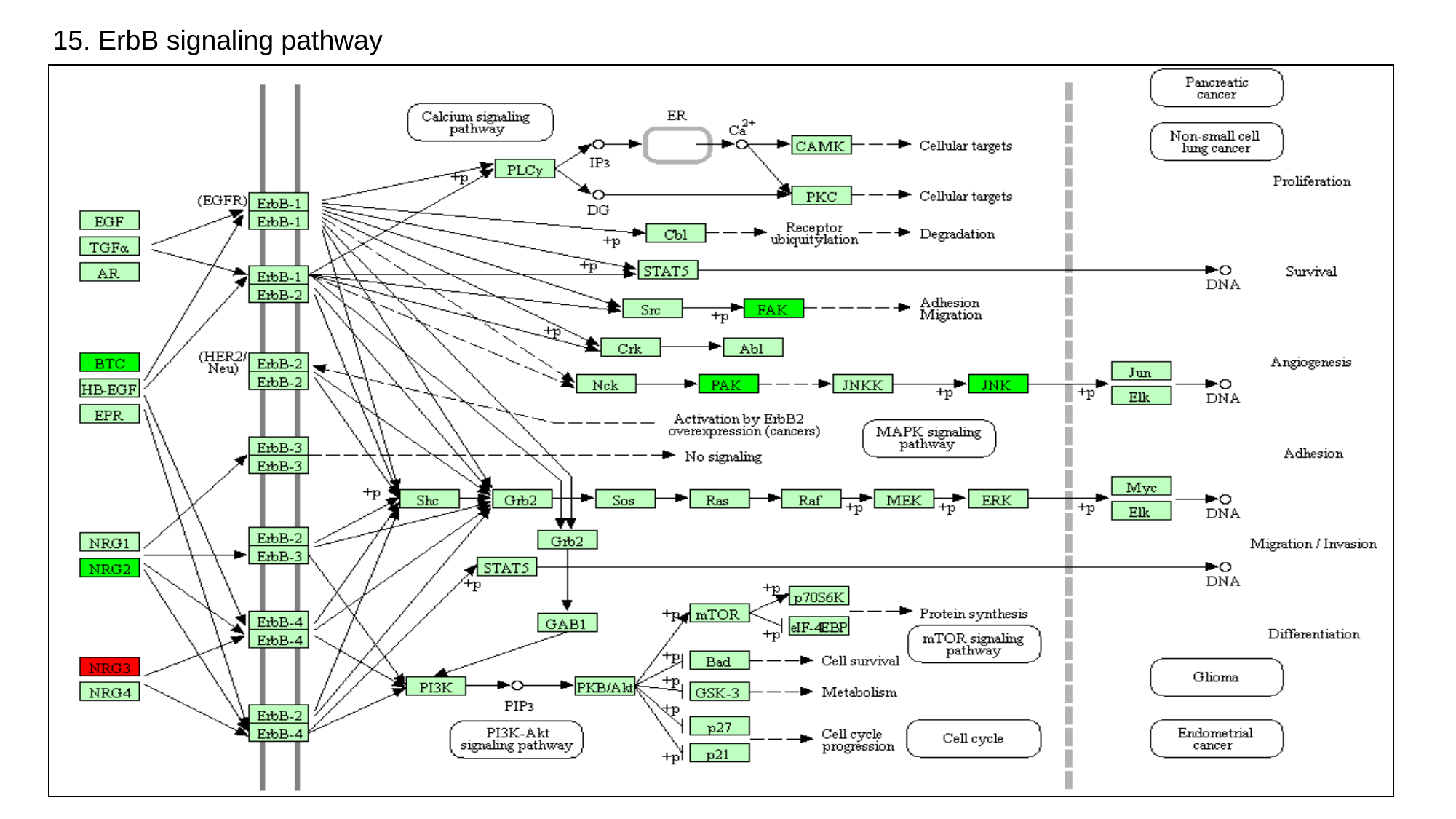

15. ErbB signaling pathway

## Slide 18
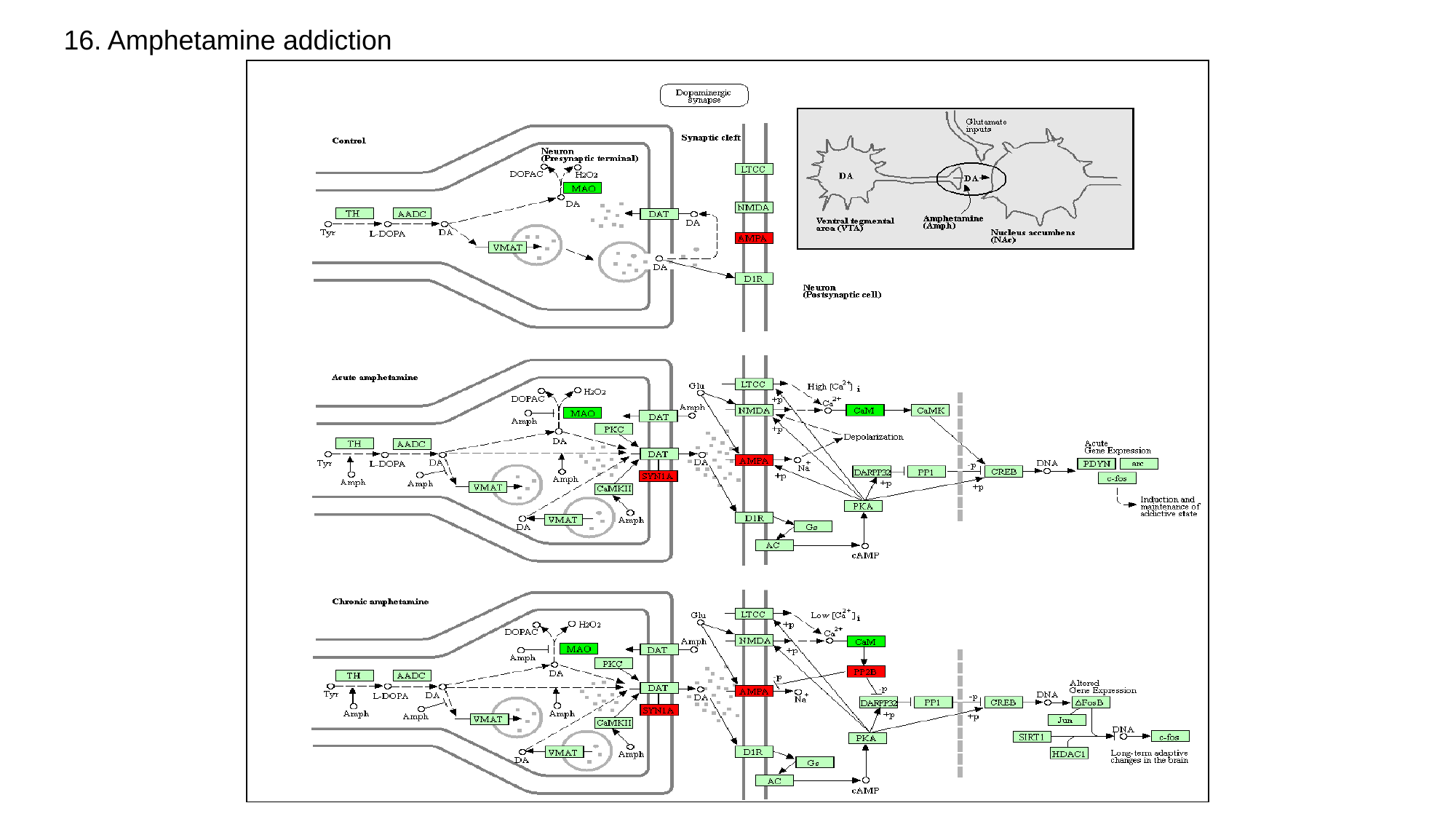

16. Amphetamine addiction
